# Improved protein structure reconstruction using secondary structures, contacts at higher distance thresholds, and non-contacts

**DOI:** 10.1186/s12859-017-1807-5

**Published:** 2017-08-29

**Authors:** Badri Adhikari, Jianlin Cheng

**Affiliations:** 1 Department of Mathematics and Computer Science, University of Missouri-St.Louis, St. Louis, MO 63121 USA; 2 Department of Electrical Engineering & Computer Science, Informatics Institute, University of Missouri, Columbia, MO 65211 USA

**Keywords:** Protein contacts, Structure reconstruction, Secondary structures, De novo structure prediction

## Abstract

**Background:**

Residue-residue contacts are key features for accurate de novo protein structure prediction. For the optimal utilization of these predicted contacts in folding proteins accurately, it is important to study the challenges of reconstructing protein structures using true contacts. Because contact-guided protein modeling approach is valuable for predicting the folds of proteins that do not have structural templates, it is necessary for reconstruction studies to focus on hard-to-predict protein structures.

**Results:**

Using a data set consisting of 496 structural domains released in recent CASP experiments and a dataset of 150 representative protein structures, in this work, we discuss three techniques to improve the reconstruction accuracy using true contacts – adding secondary structures, increasing contact distance thresholds, and adding non-contacts. We find that reconstruction using secondary structures and contacts can deliver accuracy higher than using full contact maps. Similarly, we demonstrate that non-contacts can improve reconstruction accuracy not only when the used non-contacts are true but also when they are predicted. On the dataset consisting of 150 proteins, we find that by simply using low ranked predicted contacts as non-contacts and adding them as additional restraints, can increase the reconstruction accuracy by 5% when the reconstructed models are evaluated using TM-score.

**Conclusions:**

Our findings suggest that secondary structures are invaluable companions of contacts for accurate reconstruction. Confirming some earlier findings, we also find that larger distance thresholds are useful for folding many protein structures which cannot be folded using the standard definition of contacts. Our findings also suggest that for more accurate reconstruction using predicted contacts it is useful to predict contacts at higher distance thresholds (beyond 8 Å) and predict non-contacts.

**Electronic supplementary material:**

The online version of this article (10.1186/s12859-017-1807-5) contains supplementary material, which is available to authorized users.

## Background

A major motivation for protein contact prediction and contact-guided protein structure prediction comes from the general finding that accurate contacts lead to accurate tertiary structural models. Studies like FT-COMAR [1] and Reconstruct [2] on protein structure reconstruction using true contacts have shown that in general three-dimensional protein structures can be recovered using two-dimensional contact maps. For instance, using true Cα contact maps derived with a distance threshold of 9 Å, a study reconstructed 19 proteins with accuracy of 1 Å RMSD [3]. Similarly, deriving true contacts at distance cut-offs higher than 9 Å, Vassura et al. reconstructed Cα models for 1760 proteins of different fold classes with RMSD of around 2 Å using the FT-COMAR method [1, 4]. In another study, authors have shown that the quality of 3D reconstruction is unaffected by deleting up to an average 75% of the real contacts [5]. Likewise, in a different study, it is demonstrated that the number of contacts needed for reconstruction can be decreased using a cone-peeling method and a reconstruction accuracy of ≤4 Å can be achieved with just around 20 to 30% of true contacts on a data set of 12 proteins [6]. Most recently, it is also shown that a distance cut-off of 9 Å to 11 Å delivers accurate reconstructions using Cβ atoms for defining contacts on a data set of 60 proteins [2].

These studies on reconstruction present many invaluable insights for utilizing contacts to fold proteins. However, in the context of reconstruction studies being useful for de novo protein structure prediction, they have some limitations. Firstly, these studies use complete contact maps to reconstruct protein structures, whereas, recent practice for most model building methods has been to use much lesser predicted contacts. Consequently, these reconstruction studies also do not comply with the widely-used contact definition, i.e., the Critical Assessment of Protein Structure Prediction’s (CASP) definition of contacts where 8 Å distance threshold is used with minimum sequence separation of 6 residues. Secondly, these studies cover the issues related to the reconstruction of all types of proteins, and do not focus on the proteins that demand de novo protein structure modeling. Since contact-guided protein modeling approaches are mostly useful when significant homologous templates are not found, it is important for reconstruction studies to focus on the proteins for which structural templates are hard to find. Lastly, none of these studies consider secondary structure information during reconstruction. Since secondary structure prediction has reached an accuracy higher than 80% [7, 8], it is meaningful to study how the knowledge of secondary structures can influence the quality of reconstructed models.

In this study, we investigate how accurately we can reconstruct ‘hard’ proteins (like the proteins categorized as ‘free-modeling’ in the CASP competitions) using true contacts and discuss various techniques to fold the ones whose structures cannot be accurately built in conventional ways. These techniques include, adjusting contact definitions, adding non-contacts into reconstruction, and incorporating secondary structure. Using our fragment-free de novo reconstruction method CONFOLD [9] to carry out the experiments, we show that these techniques are useful to improve contact-based protein structure reconstruction.

## Results

As the first step of testing our reconstruction pipeline, we reconstructed the 12 protein structures used by Duarte et al. [2] as benchmark dataset and compared our results with their tool Reconstruct. For the comparison, we ran the Reconstruct tool locally to generate 20 models for each protein and the CONFOLD method to generate 20 models. Then, we considered best of the 20 models, by each method, for evaluation. Table 1 shows that our method reconstructs more accurate models (20% improvement in RMSD) than Reconstruct when we compare the best models reconstructed by the two methods. Evaluation and comparison using other standard metrics like TM-score and GDT-TS score [10] also confirms that CONFOLD reconstructs better models. In summary, we observe that our method can reconstruct full atom tertiary structures of various folds with accuracy at least as good as the state-of-the art method Reconstruct.

**Table 1:** Comparison of the best of 20 models reconstructed using CONFOLD with the best of 20 models reconstructed using Reconstruct on the 12 benchmark proteins

PDB code - chain ID	SCOP class	L	Reconstruct			CONFOLD		
			TM-score	RMSD	GDT-TS	TM-score	RMSD	GDT-TS
1bkr-A	all-α	109	0.88	1.54	81.02	0.89	1.61	85.42
1odd-A	all-α	118	0.85	1.62	78.75	0.87	1.56	83.75
1cem-A	all-α	363	0.81	2.20	63.91	0.96	1.53	80.79
1pzc-A	all-β	123	0.91	1.38	85.04	0.91	1.28	84.84
1onl-A	all-β	128	0.91	1.42	83.86	0.91	1.39	84.65
1eur-A	all-β	365	0.83	2.04	68.98	0.96	1.42	83.38
1e6k-A	α/β	130	0.89	1.75	82.50	0.91	1.42	82.69
1o8w-A	α/β	146	0.90	1.65	79.72	0.91	1.50	82.52
1ede-A	α/β	310	0.95	1.61	82.26	0.96	1.40	82.58
1r9h-A	α + β	135	0.85	1.83	78.60	0.87	1.75	81.14
1ugm-A	α + β	125	0.85	1.88	77.21	0.87	1.71	80.53
1iu4-A	α + β	331	0.83	4.19	63.29	0.93	1.93	77.04
**Average**		**199**	**0.87**	**1.93**	**77.10**	**0.91**	**1.54**	**82.44**

Models are evaluated using TM-score, RMSD (in Å), and GDT-TS scores. Proteins are identified by their PDB ID followed by the chain ID. L is the length of the protein chain

### Reconstruction of CASP 8, 9, 10 and 11 domains using contacts

We reconstructed the structures for a total of 496 structural domains of the proteins released as regular targets in CASP 8, 9, 10 and 11 experiments using CONFOLD method with the true contacts derived from their native structures. The accuracy of reconstructing these structural domains, summarized in Table 2, shows that the mean TM-score [10] and RMSD of the reconstructed models is 0.78 and 3.2 Å. Our mean RMSD (3.2 Å) appears much higher than the expected mean RMSD of 2 Å as suggested in [4] because we did not consider local contacts (residue pairs closer than 6 residues in sequence) in order to comply with the currently widely accepted CASP’s definition of contacts. CASP defines that residues must be separated by at least 6 residues to be in contact. In other words, we used all short-, medium-, and long-range contacts but not the complete contact map. To validate our assumption that the decrease in accuracy is because of the exclusion of the local contacts, we repeated our reconstruction experiments by including the contacts with sequence separation less than 6 residues and obtained mean TM-score and RMSD of 0.86 and 2.2 Å respectively. In addition, for each of the 496 domains, we also reconstructed 20 models using another reconstruction method FT-COMAR [1]. FT-COMAR’s average reconstruction accuracy for these domains is 4.9 Å when measured using RMSD and 0.68 when measured using TM-score, when best of 20 models are evaluated, much lower than the accuracy of CONFOLD’s models (see Additional file 1: Table S1 for complete results and detailed comparison). These results confirm existing findings that in general, local contacts are useful for reconstructing high-resolution models.

**Table 2:** Reconstruction accuracy of 496 free-modeling (FM), template-based modeling (TBM), and hard template-based modeling (TBM-HA) domains in CASP 8, 9, 10 and 11 as measured by TM-score and RMSD

Group	Domain Count	TM-score	RMSD
FM	72	0.69	4.57
TBM-HA	71	0.78	3.24
TBM	350	0.80	2.88
Other	3	0.87	2.33
**All**	**496**	**0.78**	**3.18**

Three domains in CASP11, which are not classified into any of the three groups are categorized in the ‘Other’ group

**Fig. 1:**
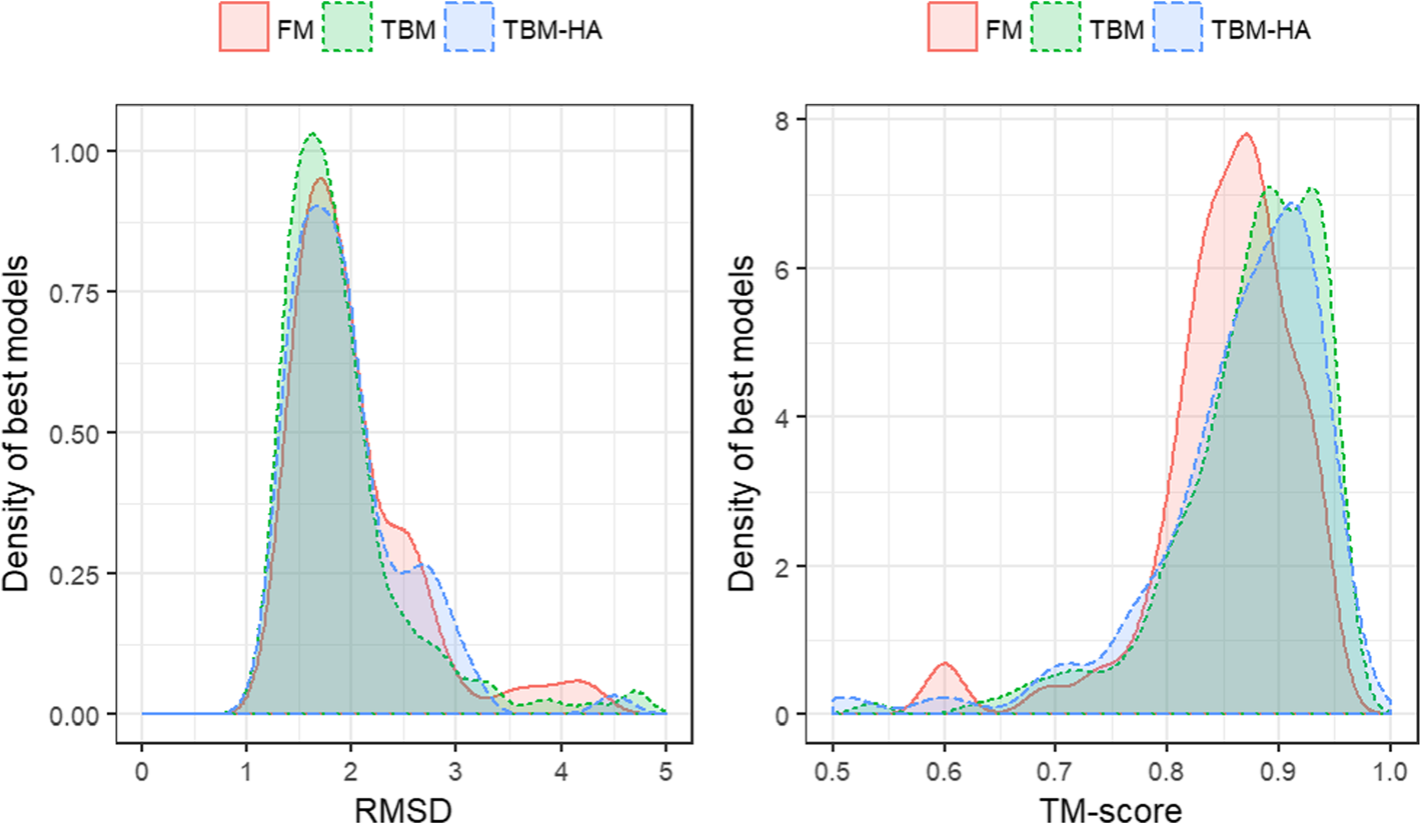
Distribution of the RMSD (**a**) and TM-score (**b**) of the best reconstructed models for the free-modeling (FM), template-based modeling hard (TBM-HA), and template-based modeling (TBM) domains in CASP 8, 9, 10, 11

From our reconstruction using the standard CASP’s definition of contacts, we find that the mean reconstruction accuracy for free-modeling (FM) targets is much lower than their template-based modeling (TBM) counterparts (see Table 2 and Fig. 1), indicating that the structures of hard targets are more difficult to reconstruct than easy targets. We also find that 28 out of the 496 domains were reconstructed with less than 0.5 TM-score, i.e. incorrect topology. In Table 3 we list these ‘hard-to-reconstruct’ domains. To ensure that the low TM-score for these domains is not due to the method’s ability to satisfy contacts, we calculated the sum of deviation (error) for all input contacts for each of the best model and found that in all cases this deviation is either zero or close to zero. This shows that the contacts restraints have been satisfied well and the low accuracy is due to the insufficiency of the input information. Almost all of these proteins are primarily helical, having 51% helix residues for the 13 FM domains and 65% for the 15 TBM domains, on average. This suggests that contact information alone (including all short-, medium-, and long-range contacts) cannot accurately guide the assembly of helices in many protein structures, and that knowing secondary structure (particularly helices) may improve the reconstruction accuracy. In the next section, we discuss the reconstruction results when secondary structures are included.

**Table 3:** List of all domains with reconstruction accuracy below 0.5 TM-score

CASP	Domain	L	Type	H	E	N_c_	TM-score	RMSD	GDT-TS	Energy
8	T0393-D2	99	TBM	74	0	50	0.29	10.6	27.8	0.0
8	T0405-D1	72	FM	58	0	67	0.42	7.2	45.5	0.0
8	T0443-D1	66	FM	41	0	42	0.45	6.6	50.0	0.0
8	T0443-D3	66	TBM	35	6	67	0.41	5.9	48.1	0.5
8	T0454-D2	140	TBM	94	0	141	0.49	6.6	40.0	1.0
8	T0470-D2	77	TBM	45	0	71	0.34	7.7	35.7	0.0
8	T0482-D1	67	FM	17	32	119	0.40	8.0	44.0	5.9
9	T0548-D2	60	TBM	43	0	45	0.42	7.4	49.6	0.2
9	T0553-D2	71	FM	46	0	59	0.49	4.6	52.8	0.0
9	T0575-D2	127	TBM	100	0	128	0.45	6.4	37.0	2.5
9	T0589-D2	82	TBM	58	0	74	0.48	5.2	48.8	0.0
9	T0598-D1	127	TBM	64	11	141	0.48	6.5	41.9	1.0
9	T0616-D1	97	FM	41	0	84	0.32	12.3	28.6	0.7
9	T0617-D1	136	TBM	96	8	143	0.49	11.8	43.2	8.1
9	T0629-D2	159	FM	0	4	31	0.16	25.2	12.1	0.0
9	T0637-D1	135	FM	109	0	75	0.33	16.1	24.4	0.3
9	T0639-D1	124	FM	76	4	133	0.36	8.3	30.9	2.7
10	T0680-D1	96	TBM	79	0	108	0.36	7.2	33.9	7.7
10	T0685-D1	72	TBM	54	0	42	0.27	8.5	31.3	0.0
10	T0693-D1	100	FM	47	12	101	0.38	14.7	34.5	1.3
10	T0724-D1	119	TBM	38	40	133	0.30	13.3	26.3	1.4
10	T0732-D2	91	TBM	48	0	91	0.44	5.8	46.2	1.5
10	T0741-D1	125	FM	0	73	218	0.45	17.1	39.0	5.8
10	T0756-D2	86	FM	45	0	15	0.25	12.0	25.9	0.0
11	T0820-D1	90	FM	65	0	72	0.40	7.3	41.9	0.0
11	T0821-D1	255	TBM	195	0	378	0.46	8.6	26.9	35.6
11	T0831-D1	155	TBM	114	0	141	0.44	15.8	34.8	1.5
11	T0836-D1	204	FM	157	0	198	0.38	12.9	22.8	7.2

The models were reconstructed with contacts only. L, H, E, and N_c_ refer to length of the protein, number of helical residues, strand residues, and number of native contacts in the native structures, respectively. TM-score, RMSD, and GDT-TS of the best-of-20 models for each domain are presented. The last column (Energy) is the sum of the distance deviation from 8 Å for all the contacts supplied as distance restraints

### Reconstruction using contacts and secondary structures

In addition to reconstruction using contacts only, we reran our experiments by adding true 3-state secondary structures restraints (coil, helix and strand). On the same data set of 496 CASP structural domains, we obtained a mean TM-score of 0.88 and RMSD of 2.0 Å (see Additional file 1: Table S1 for complete results). This accuracy is slightly higher than the accuracy (TM-score = 0.86 and RMSD = 2.2 Å) when using complete contact maps (i.e., including contact pairs closer than 6 residues). The slightly higher TM-score and lower RMSD due to the use of secondary structure information suggests that aiding contacts with secondary structures is more useful than including the local contacts without secondary structure information. The improvement from using secondary structures and true contacts is significant according to paired t-test of TM-scores between the models reconstructed with contacts and secondary structures and the models reconstructed using the whole contact map without secondary structures (*p*-value = 2.2 × 10^−16^). We also observed that out of the 28 protein domains that had less than 0.5 TM-score when reconstructed with contacts only, 24 of them have TM-score higher than 0.5 after adding secondary structures. The remaining 4 domains (out of 28) listed in Table 4 could not be reconstructed accurately (with TM-score > 0.5) using true contacts despite being supplemented by true secondary structures. Among these domains, T0629-D2 is a domain in a long tail needle-shaped receptor-binding tip protein 2XGF, T0693-D1 is a small helical region in the alpha-beta protein 4P7C, T0741-D1 is a V-shaped protein with two long beta hair-pins, and T0756-D2 is a helix bundle domain in the alpha-beta protein 4G6Q.

**Table 4:** List of CASP domains for which reconstruction could not recover the fold (a) using contacts only or (b) using contacts and secondary structures

CASP	Domain	L	H	E	Without SS			With SS		
					TM-score	RMSD	GDT-TS	TM-score	RMSD	GDT-TS
9	T0629-D2	159	0	4	0.16	25.2	12.1	0.16	21.4	12.4
10	T0693-D1	100	76	4	0.38	14.7	34.5	0.44	12.0	41.8
10	T0741-D1	125	0	73	0.45	17.1	39.0	0.39	13.1	32.8
10	T0756-D2	86	45	0	0.25	12.0	25.9	0.38	15.4	39.5

TM-score, RMSD, and GDT-TS of the best-of-20 models for each domain are presented. L, H, and E, refer to the length of the protein, number of helical residues, and number of strand residues, respectively

**Fig. 2:**
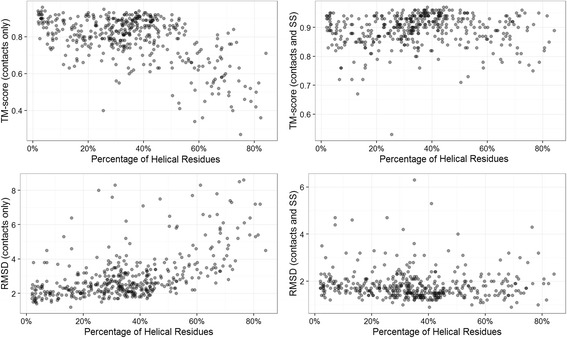
Analysis of the impact of the presence and absence of helix information on reconstruction. TM-score (plots in top row) and RMSD (plots in bottom row) of the best models when reconstructed without secondary structures (left two plots) and with secondary structures (right two plots)

To investigate why helical proteins have much higher reconstruction accuracy with secondary structure input, we calculated the correlation between the percentage of helical residues in the proteins and reconstruction accuracies. For this, we selected all structural domains having at least one helix residue and computed the correlation between the percentage of helical residues in the proteins against the RMSD of the best models reconstructed with and without secondary structure input. When the reconstruction was carried out without secondary structures, we observed a Spearman’s rank correlation coefficient of 0.58, between the percentage of helical residues and RMSD, suggesting that having more helical residues in a structure is likely to make the reconstruction more difficult. Then, we re-computed the correlations by adding secondary structures. When the reconstructions were aided by secondary structures, the Spearman’s rank correlation coefficient dropped to −0.14 (see Fig. 2). This suggests that adding secondary structure information makes reconstruction accuracy nearly independent of the composition of helices in a protein. To check if a similar pattern is observed in beta proteins, we selected all domains having at least one beta strand, and calculated the Spearman’s rank correlation coefficient between the best models’ RMSD and the percentage of beta strand residues. In case of the beta proteins we found the correlation coefficient to be 0.15 when no secondary structures are used, suggesting no such correlation between difficulty of reconstruction and the number of strand residues in structures.

### Reconstruction at higher distance thresholds for defining contacts

**Fig. 3:**
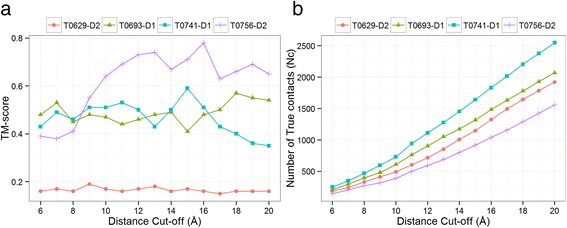
Improvement in reconstruction of ‘hard to reconstruct’ protein domains in CASP versus the increase distance cut-off thresholds (**a**) and the increase in number of contacts versus the increase of distance thresholds (**b**)

It is known that some structures are difficult to fold with some distance thresholds of defining contact. For instance, Human Myeloperoxidase Isoform C (1cxp chain B, 104 residues, all-alpha) could only be folded at a distance threshold of 16 Å instead of the more widely used 8 Å threshold [4]. For this protein structure, the authors showed that the RMSD drops from 41 Å to 4.9 Å when the contact distance threshold is increased from 7 Å to 16 Å. Similarly, in another work, authors found 14 Å distance threshold useful and reconstructed 87 protein chains using the same definition [11]. In this spirit, we tried to reconstruct the four ‘hard-to-reconstruct’ domains (T0629-D2, T0693-D1, T0741-D1, and T0756-D2) using various distance thresholds ranging from 8 Å to 20 Å. By testing these various distance thresholds along with secondary structure restraints, 3 out of the 4 structure domains could be correctly folded (TM-score > 0.5) with at least one of the distance thresholds (see Fig. 3a **)**. These observations lead us to conclude that the reconstruction at higher distance thresholds can be useful for at least some structural folds. We find that the primary reason for more accurate reconstruction at the higher distance thresholds, is that increasing distance thresholds increases the number of contact restraints (see Fig. 3b), thereby increasing the coverage of contacts and being particularly useful for many structural folds. The challenge, however, is that not all structures can be equally accurately folded at one distance threshold.

**Fig. 4:**
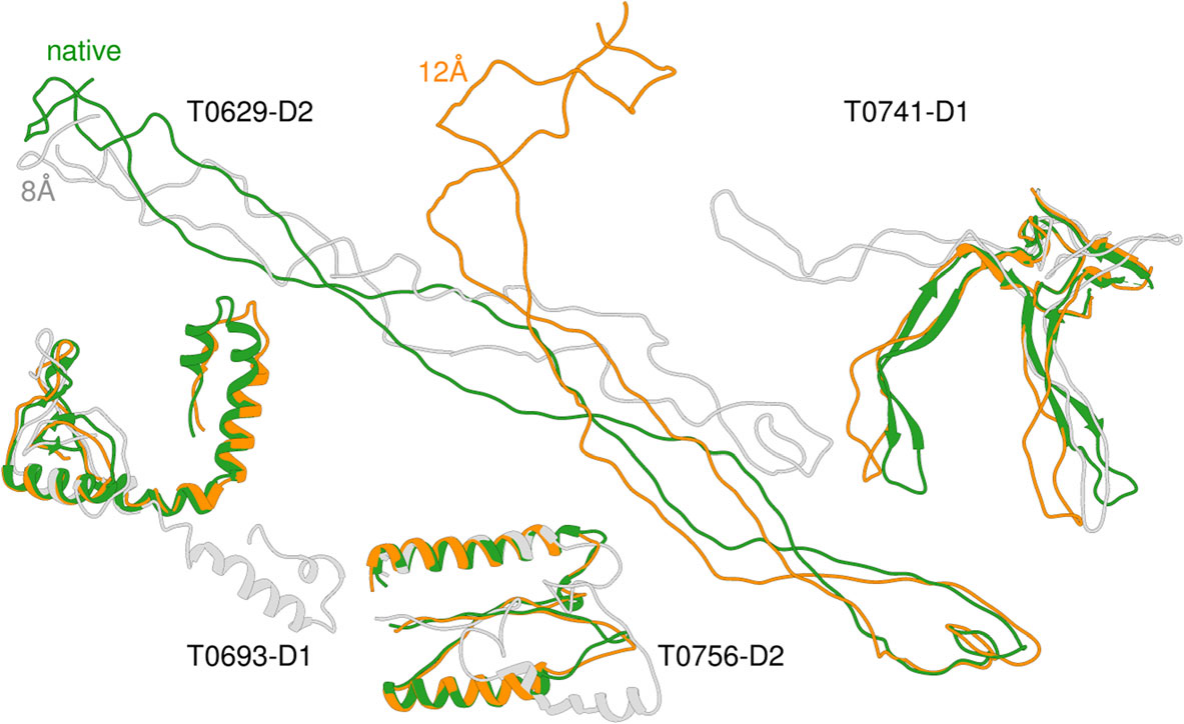
The true (native) structures of the domains T0629-D2, T0693-D1, T0741-D1, and T0756-D2 shown in green superimposed with structures reconstructed at distance cut-off of 8 Å (shown in grey), and at 12 Å (shown in orange)

Absence of secondary structure elements in the structure, we find, is one reason for low reconstruction accuracy for these hard-to-fold proteins. One of these four structures, 159-residue domain T0629-D2, was the most difficult to reconstruct primarily because of its lack of secondary structure. In fact, among all 496 CASP domains, this domain has the minimum percentage of secondary structure elements, i.e. 3%. Among the domains having minimum percentage of secondary structure elements, the next one is T0650-D1 with 20% of the residues forming secondary structures. The best model for this domain has GDT-TS of 0.5. Figure 4 visualizes these four proteins showing how their non-globular structures impose challenges on reconstruction.

### Reconstruction with non-contacts

**Fig. 5:**
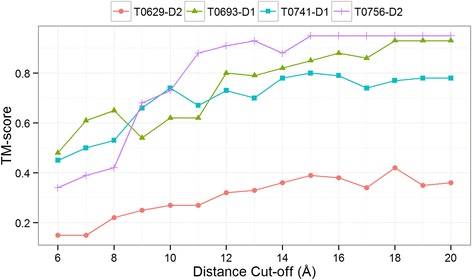
Reconstruction of the four hard-to-reconstruct CASP domains T0629-D2, T0693-D1, T0741-D1, and T0756-D2 using contacts and non-contacts at various contact thresholds

Different from all existing methods that use only contact information for reconstruction, we tested if adding non-contact information (a pair of residues whose distance is greater than a defined distance threshold) can increase the accuracy of reconstruction. To begin, we selected the same four hard-to-reconstruct proteins and reconstructed their models using both contacts and non-contact as restraints at various distance thresholds. Figure 5 shows that at higher distance thresholds, non-contact information is surprisingly informative for reconstructing high-quality structures for three out of these four proteins. For at least one of the many distance thresholds, two of the four domains (T0693-D1 and T0756-D2) were reconstructed with around 1 Å RMSD and the third one (T0741-D1) with 2 Å RMSD. The hardest structure, T0629-D2, although showing some improvement with non-contacts, still could not be folded, suggesting, again, that (a) some folds are hard to reconstruct, and (b) structures without secondary structure elements are among the most challenging structures to be reconstructed. For this domain (T0629-D2), to test if the knowledge of the quaternary structure of the domain could be useful for the reconstruction of the domain, we reconstructed the whole protein, with PDB ID 2XGF having 648 residues. Best-of-20 model, from such a reconstruction, had a TM-score of 0.32, suggesting that the knowledge of quaternary structure could not recover the fold of the domain.

**Fig. 6:**
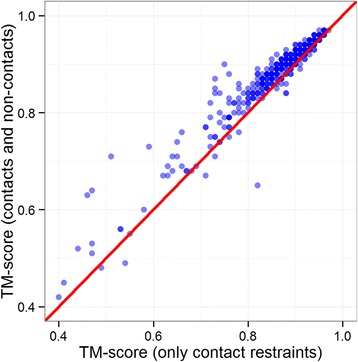
Improvement of adding non-contacts as restraints for CASP 8, 9, 10 and 11 target domains. (a) using contacts and secondary structure, and (b) using contacts and non-contacts together with secondary structures

For a more rigorous testing, we repeated our reconstruction tasks for all the 496 CASP domains using contacts defined at 8 Å threshold and the corresponding non-contacts. Specifically, we supplied the residue pairs not defined in true contacts list as non-contact restraints to CONFOLD, and observed around 2.5% improvement in TM-score on average. Figure 6 shows that for 479 out of 496 structures, the accuracy either stays same or improves, suggesting that adding non-contact restraints improves the model reconstruction accuracy in most cases. This improvement from the addition of non-contacts is significant according to paired t-test of TM-scores between the models reconstructed with contacts and non-contacts and the models reconstructed using contacts only (*p*-value = 2.2 × 10^−16^) (see Additional file 1: Table S1 for detailed results).

### Shape of the structures and reconstruction difficulty

Using our largest dataset of 1901 proteins in the SCOP classification dataset, we reconstructed the structures using true contacts derived from the structures, to investigate the difficulty of reconstruction across various SCOP classes, and how this difficulty varies after inclusion of non-contacts. Our reconstruction results summarized in Table 5, which agree with the findings of [1], show that the average TM-score of the reconstructed models for class C proteins (alpha and beta (a/b) proteins) is 0.923 and are the easiest to reconstruct, followed by the class A (all alpha), B (all beta), and D (alpha and beta a + b). Similarly, the average TM-scores for membrane and cell surface proteins (class F) is 0.72, suggesting that the class is hardest to reconstruct. The smaller average TM-score of 0.68 for small proteins (class G) does not necessarily suggest that they are hardest proteins to reconstruct because the TM-score evaluation is not expected to perform well for short proteins [10]. This conclusion is supported by our observation that the average RMSD for the small proteins (3 Å) is much lower than the average RMSD for membrane and cell surface proteins (4.5 Å).

**Table 5:** Reconstruction summary of the 1901 structural domains in SCOP dataset showing the reconstruction accuracy when only contacts are used and when non-contacts are added along with contacts

SCOPe Class	Class Description	Number of Domains	Using Contacts Only		Using Contacts and Non-Contacts	
			TM-score	RMSD	TM-score	RMSD
A	All alpha proteins	500	0.829	2.74	0.854	2.46
B	All beta proteins	349	0.851	2.43	0.873	2.19
C	Alpha and beta proteins (a/b)	232	0.923	1.84	0.932	1.68
D	Alpha and beta proteins (a + b)	538	0.856	2.46	0.878	2.22
E	Multi-domain proteins (alpha and beta)	49	0.853	3.47	0.878	3.14
F	Membrane and cell surface proteins	102	0.719	4.54	0.745	4.08
G	Small proteins	131	0.680	3.02	0.717	2.50
**Total/Average**		**1901**	**0.816**	**2.928**	**0.840**	**2.610**

Best of 20 reconstructed models are reported

**Fig. 7:**
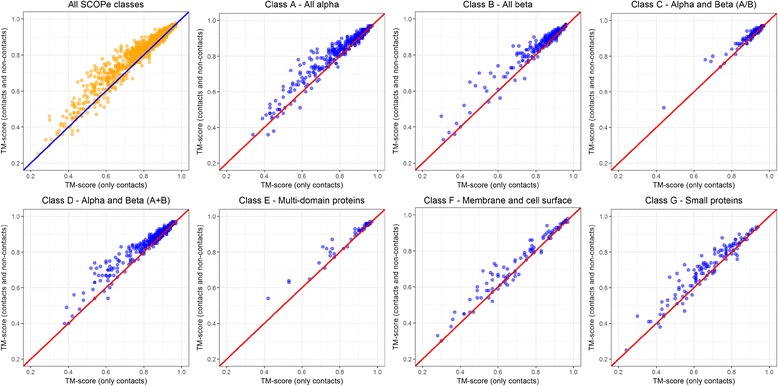
Improvement in reconstruction accuracy by using non-contacts together with the true contacts for all the 1901 proteins in the SCOP dataset and the seven classes (subsets). TM-scores of the best models reconstructed with contacts only are plotted against the TM-scores of the best models reconstructed with contacts and non-contacts

Furthermore, as shown in Table 5, on this large dataset, adding non-contacts improves the average TM-score of the reconstructed models to 0.84 from 0.816. Figure 7 shows that the improvement from adding non-contacts is observed in all fold classes – all alpha proteins (class A), all beta proteins (class B), alpha and beta proteins (class C), alpha and beta proteins (class D), multi-domain proteins (class E), membrane and cell surface proteins (class F), and small proteins (class G). The addition of non-contacts, on average, improves the reconstruction accuracy for all protein classes but does not alter the relative difficulty of the classes.

### Reconstruction at various sequence separation thresholds

**Fig. 8:**
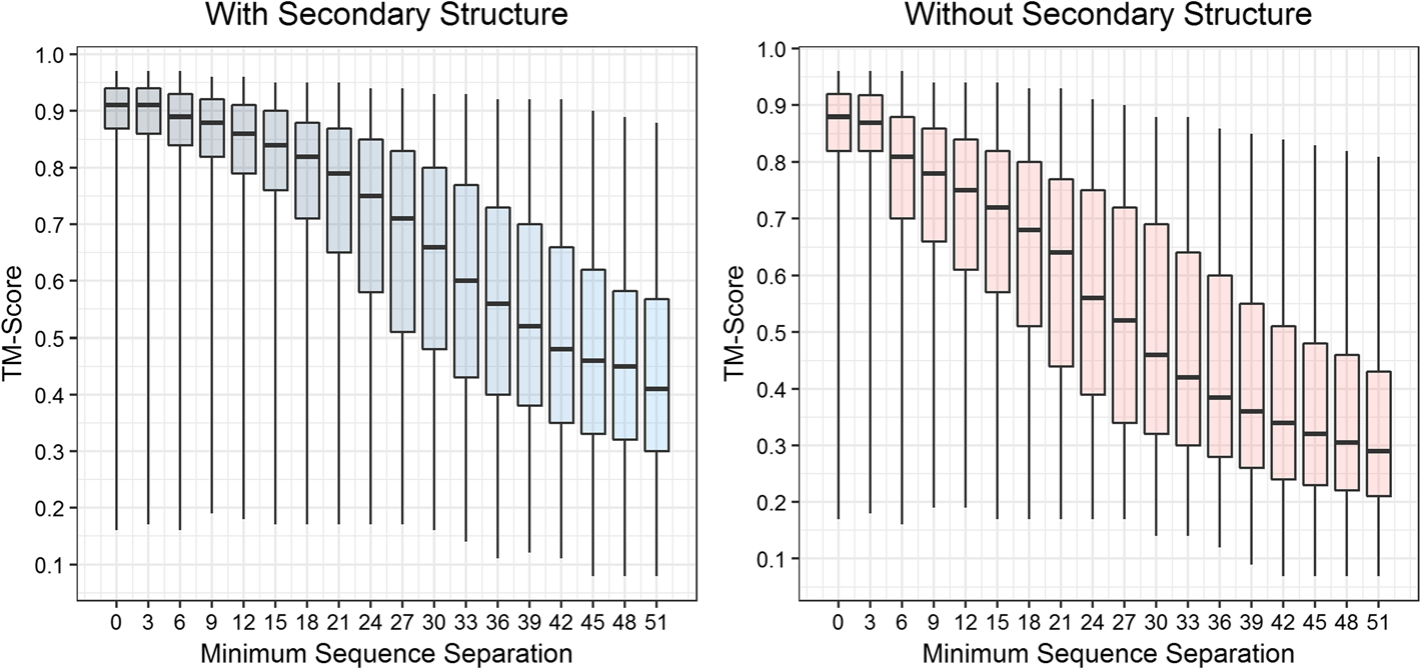
Reconstruction accuracy against various thresholds for sequence separation (for selecting contacts) on the 496 proteins in the CASP dataset

It is widely understood that long range contacts (sequence separation of at least 24 residues) are the most important of the three contact types – short-, medium-, and long-range. To study how sequence separation affects the reconstruction accuracy of proteins, we reconstructed all the 496 CASP domains by removing contacts at various sequence separation thresholds, with and without the knowledge of secondary structure. Specifically, for each CASP structural domain, we removed all contacts closer than x residues in the corresponding sequence, where x = {0, 3, 6, …, 51}, and reconstructed models using CONFOLD, with and without three-state secondary structure information. Figure 8 shows that when secondary structures are used in reconstruction, the gain in accuracy from the use of local contacts (with sequence separation less than 6) is much lower. On average, when models are reconstructed using contacts, the mean reconstruction TM-scores at minimum sequence separation threshold of 6, 12, and 24 residues are 0.78, 0.74, and 0.55, respectively. Similarly, when secondary structures are added, the mean reconstruction TM-scores at minimum sequence separation threshold of 6, 12, and 24 residues are 0.88, 0.85, and 0.75, respectively. Setting sequence separation thresholds to 6, 12, and 24 correspond to removing local contacts, short-range contacts, and medium-range contacts, respectively. The relatively large drop in the accuracy at the sequence separation threshold of 24 residues suggests that compared to local contacts and short-range contacts, medium-range contacts are very important for reconstruction.

## Discussion

Realizing the importance of contact definition at higher distance thresholds, tools like NNcon [12] predict contacts at both distance thresholds – 8 Å and 12 Å. There are, however, challenges in predicting contacts at higher distance thresholds and utilizing them to build models. The first challenge is that the number of contacts increases rapidly as the distance threshold increases, making it harder for reconstruction methods to decide the number of contacts to consider for modeling. The second challenge is deciding the threshold that works for all proteins. Although the threshold of 8 Å between Cβ atoms is widely used, many studies demonstrate otherwise. For instance, Vassura et al., using a large data set of 1760 proteins, found that increasing the distance threshold up to 18 Å improves the reconstruction accuracy monotonically. Similarly, Duarte et al., using a data set of 60 proteins, found that the best reconstruction accuracies were obtained with distance thresholds between 9 and 11 Å. Although these studies do not agree on the optimal cut-off distance, all of them demonstrate that contact restraints at higher distance thresholds are useful.

**Fig. 9:**
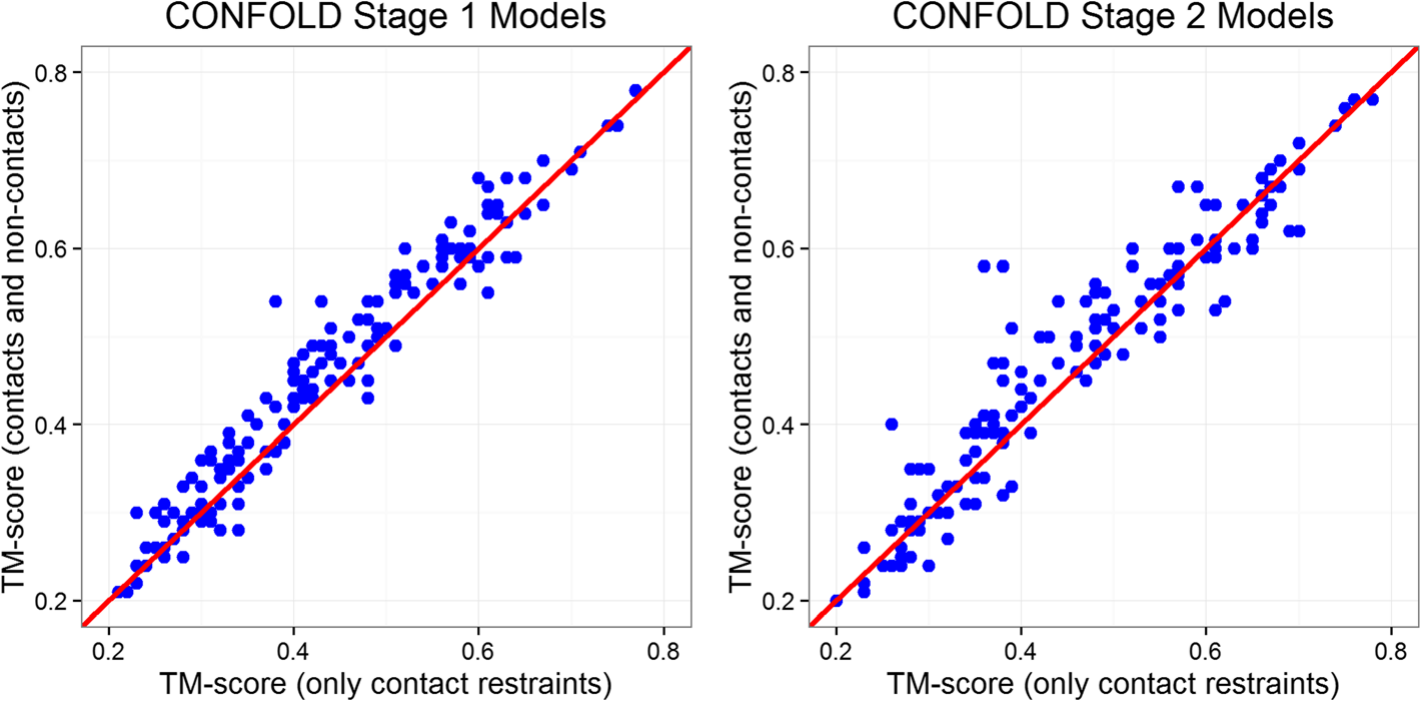
Improvement in reconstruction accuracy by using predicted non-contacts together with the predicted contacts for the 150 proteins in the PSICOV dataset in reconstruction stage 1 (left) and reconstruction stage 2 (right) of CONFOLD

Following our finding that true non-contacts can help structure reconstruction, as the next step, we studied if predicted non-contact information can improve de novo contact-guided modeling. For this we chose the contacts predicted by PSICOV for the 150 proteins [13] and built models with predicted contacts and compared with the models built using predicted contacts as well as predicted non-contacts. For predicting non-contact information, we did not use any additional method. Instead, in the same set of contacts predicted by PSICOV, we considered the contacts predicted with lowest confidence score (those having negative confidence values) as predicted non-contacts. Specifically, we selected top L predicted pairs as contacts and selected all pairs with predicted confidence less than −1 as predicted non-contacts. While the predicted contacts were translated into distance restraints of 3.5 Å to 8 Å between corresponding Cβ atoms, non-contacts were translated to distance restraints of 10 Å to 200 Å between corresponding Cβ atoms. We found that setting a slightly higher distance threshold of 10 Å instead of 8 Å yields better reconstruction accuracy. With these contacts and non-contacts, we reconstructed 20 models using CONFOLD and selected best model generated at reconstruction stages 1 and 2 for analysis. Figure 9 shows that adding non-contact information improves the accuracy of the best reconstructed models for most proteins. When we selected residue pairs with confidence less than −1 as non-contacts, we observed 5% improvement in the TM-score on average; and 1.5% improvement with −2 as the threshold. This improvement from adding non-contacts is significant according to the paired t-test of TM-scores between the models in the second stage reconstructed with both contacts and non-contacts (selected with contact prediction confidence less than −1) and the models in the second stage reconstructed with contacts only (*p*-value = 4 × 10^−5^). Similar significant difference was observed when we compared the models in the first stage (*p*-value = 7 × 10^−14^) (see Additional file 2: Table S2 for details). We believe that better non-contact selection techniques can improve the reconstruction accuracy to much higher ranges.

**Fig. 10:**
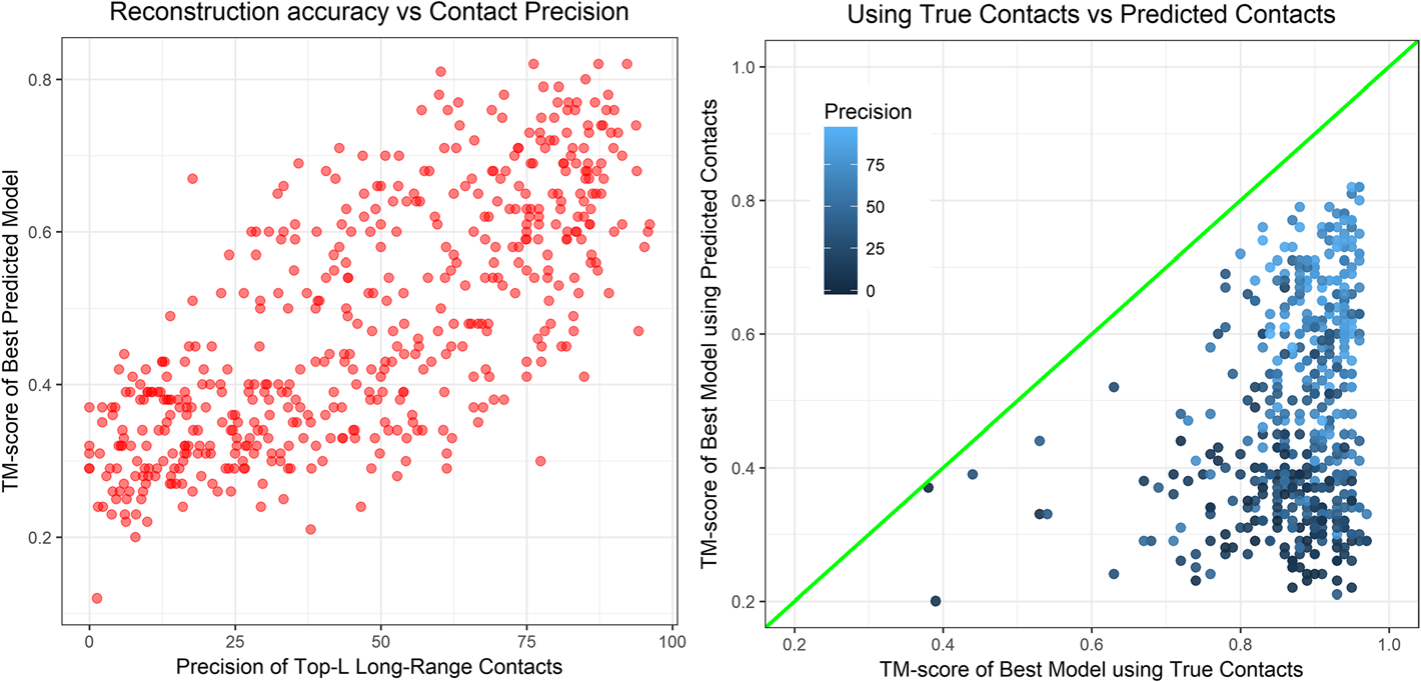
TM-scores of CONFOLD’s best predicted model plotted against the precisions of top-L long-range contacts (left) and TM-scores of the best models reconstructed using true contacts plotted against the TM-scores of the best model reconstructed using predicted contacts (right) on the CASP domains dataset

Finally, using the contacts predicted by MetaPSICOV [14] for the 496 structural domains in the CASP dataset, for each input sequence, we built models using CONFOLD. Our results, summarized in Fig. 10, show that the accuracy of the reconstructed model (model having highest TM-score) is highly correlated to the precision of the predicted contacts, and the Pearson’s correlation coefficient between the TM-score of the best predicted model and the precision of top L long-range contacts is 0.74. Compared to the average TM-score of 0.69, 0.78, and 0.80 for free-modeling (FM), template-based modeling hard (TBM-HA), and template-based modeling (TBM) domains when true contacts and secondary structures are used, when predicted contacts and secondary structures were used, we obtained average TM-scores of 0.40, 0.48, and 0.50 for FM, TBM-HA, and TBM domains, respectively. As expected, the relative difficulty of reconstruction between free-modeling domains and template-based domains is also pronounced when predicted contacts are used (see Additional file 3: Table S3 for detailed head-to-head comparison).

## Conclusions

In this study, we revisited the problem of protein structure reconstruction using true contacts focusing on the proteins whose structures are hard to predict. We show that increasing the distance threshold for defining contacts, using secondary structures, and adding non-contacts can improve the reconstruction accuracy of protein structures, particularly the ones that are hard to fold. Our findings provide useful insights to improve existing contact prediction and structure reconstruction/folding methods.

## Methods

### Contact definition

In this work, we define a pair of residues to be in contact if the distance between their Cβ atoms (Cα in glycine) is less than 8 Å. Contacts separated by 6 to 11 residues in the corresponding sequence are categorized as short-range, contacts separated by 12 to 23 residues are categorized as medium-range, and those separated by 24 or more residues are defined as long-range contacts. In addition, we define contacting pairs, which are closer than 6 residues in the sequence as ‘local’ contacts. Local, short-range, medium-range, and long-range contacts all together make the complete contact map of a protein.

### Data sets

For comparison with Reconstruct [2], we used the data set of 12 proteins used to benchmark it (see Table 2 for the list of proteins). Similarly, for our analysis involving CASP’s data sets, we considered all regular target domains released in CASP 8, 9, 10 and 11 having at least 60 residues. Domains like T0605-D1 that have no native contacts were also excluded from our data set. Our final data set consisted of 496 structural domains consisting of 72 free-modeling (FM) domains, 71 hard template-based modeling (TBM-HA) domains, 350 template-based (TBM) domains, and 3 ‘other’ domains (see Table 6).

**Table 6:** Number of free-modeling (FM) and template-based modeling (TBM) domains in CASP 8, 9, 10 and 11 competitions

	FM	TBM-HA	TBM	Other	Total
CASP-8	8	48	93	0	149
CASP-9	23	3	106	0	132
CASP-10	11	12	89	0	112
CASP-11	30	8	62	3	103
**Total**	**72**	**71**	**350**	**3**	**496**

In addition to the two datasets, for studying the reconstruction difficulty of various protein shapes (fold classes), we curated a structure dataset by selecting one protein from each superfamily within each fold of the seven classes (class A through G) of SCOP 2.04 database [15]. Since some of the proteins have many domains and are relatively very long, we removed all the proteins longer than 450 residues from our set. Our final set consisting of total 1901 proteins, has 500 all alpha proteins (class A), 349 all beta proteins (class B), 232 alpha and beta proteins (a/b) (class C), 538 alpha and beta proteins (a + b) (class D), 49 multi-domain proteins (class E), 102 membrane and cell surface proteins (class F), and 131 small proteins (class G).

### Reconstruction using true contacts

In order for our study not to be influenced by additional information (like information about structural fragments), we used our CONFOLD [9, 16] method to build models, which uses purely contacts (and secondary structure information when supplied) to build models. For reconstruction tests that involve using contacts only, we obtained contacts from the native structures/domains, and used them as input to CONFOLD to build 20 models. For evaluating the reconstructed models we use Template-Modeling score (TM-score), RMSD, and Global Distance Test (GDT-TS) score [10] and used the best of the 20 models for each target for assessment.

Following this protocol, we reconstructed the structural models of 12 proteins in the Reconstruct [2] dataset, as a benchmark for our reconstruction pipeline. Then we reconstructed models for the 496 proteins in the CASP 8, 9, 10, and 11 datasets using true contacts derived from the native structure. In addition, to study the relationship between the shape of the proteins and the difficulty of reconstruction, we reconstructed models for the 1901 proteins from the SCOP 2.04 [15] classification belonging to the seven classes (class A through G).

### Reconstruction using contacts and secondary structures

In all the reconstruction experiments where we use true contacts and secondary structures, we derived secondary structures from the corresponding native structure using DSSP [17]. From the various DSSP assignments to each residue (strand, turn, alpha-helix, etc.), we translate all assignments except stand (E) and alpha-helix (H) to coil (C), such that our true secondary structures are in the same 3-state format as predicted contacts. For reconstruction, CONFOLD translates the input contacts into distance restraints, and secondary structures into distance restraints, dihedral angle restraints, and hydrogen-bond restraints (see the CONFOLD paper [9] for details). Following this protocol, we derived true contacts and secondary structures for two datasets (a) 496 proteins in the CASP dataset, and (b) 1901 proteins in the SCOP dataset. We generated 20 models for each protein and used the best model for our analysis and comparison with the models reconstructed using contacts only (without secondary structures).

### Reconstruction using non-contacts and contacts at higher distance thresholds

From the dataset of 496 CASP structural domains, for the domains whose fold could not be recovered from reconstruction (i.e. TM-score of the best model is less than 0.5), we considered (a) increasing the threshold to define contacts, and (b) adding non-contacts along with contacts as restraints. Specifically, for each domain, we derived contacts between the carbon-atoms (Cβ) of the residues from the native structure with minimum distance thresholds ranging from 8 Å to 20 Å and reconstructed models using these contacts. In addition, for such proteins, we also tested by providing non-contacts as an additional information (along with contacts) for reconstruction.

### Contact prediction and reconstruction

In addition to the reconstructions using true contacts, for all the 496 CASP structural domains, instead of using true contacts and secondary structures, using the domains’ sequence as input we predicted contacts and secondary structures and built models, to study the relationship between the models built using predicted and true contacts, and to study the relationship between predicted contact precision and reconstruction accuracy. For this, we predicted contacts using the state-of-the-art contact prediction method MetaPSICOV [14] and 3-state secondary structures using PSIPRED [18]. Many of the features needed by MetaPSICOV rely on the quality of multiple sequence alignments generated from the input sequence. For generating input multiple sequence alignments we used HHblits [19] and JackHMMER [20] as discussed in [21]. Using MetaPSICOV’s second stage contact predictions as input, we build 5 models with top xL contacts as input to CONFOLD, where x = {0.1, 0.2, 0.3, …, 4.0} generating a total of 200 models for each protein. For our evaluation, we considered the best of these 200 predicted models.

## Additional files

Additional file 1 **Table S1.** Best of 20 models reconstructed for CASP 8, 9, 10 and 11 target domains (a) without secondary structure information and local contacts with sequence separation less than six removed (column ‘without SS (sep = 6)’), (b) without secondary structure information and no sequence separation threshold (column ‘without SS (sep = 1)’), (c) with secondary structure information and sequence separation threshold of six residues (column ‘with SS (sep = 6)’), (d) with secondary structures and non-contacts (column ‘with SS & NonC’), and (e) using FT-COMAR. The number of Helix (H) and Strand (E) residues calculated using DSSP is included along with the number of contacts in the native structure (Nc). TM-score, and RMSD of the best of 20 generated models are reported. L is the length of the domain structure and column ‘Type’ specifies template-based domain (TBM), free-modeling domain (FM), or hard template-based modeling domain (TBM-HA). For some large structures, where reconstruction tasks with non-contact restraints failed because of having too many restraints, are indicated with a dash (−). (DOCX 90 kb)

Additional file 2 **Table S2.** Accuracy of models reconstructed using CONFOLD for the 150 proteins in the PSICOV data set using (a) predicted contacts and (b) contacts and non-contacts and non-contacts. Top-L predicted contacts were considered for all tasks and best-of-20 models are evaluated. For selecting non-contacts confidence thresholds of −2 and −1 were used (results presented in separate columns). (DOCX 27 kb)

Additional file 3 **Table S3.** Comparison of reconstruction using true contacts and secondary structures vs predicted contacts and secondary structures for the 496 CASP structural domains. The columns L, H, E, and N_c_ refer to the length of the domain, number of helical residues in the native structure, number of strand residues in the native structure, and the number of contacts in the native structure, respectively. TM-score and RMSD for the best of 20 model reconstructed using true contacts and best of 200 models predicted using predicted contacts are reported. Precision of top L/5, L/2, L, and 2 L contacts are reported when all contacts are evaluated and when only long-range contacts are evaluated. (DOCX 99 kb)

## Abbreviations

CASP: critical assessment of techniques for protein structure prediction
FM: template-free modeling
PDB: protein data bank
RMSD: root mean square deviation
SCOP: structure class of protein database
TBM: template-based modeling
