# Supplementary material for: Improved protein structure reconstruction using secondary structures, contacts at higher distance thresholds, and non-contacts
Source: BMC Bioinformatics. 2017 Aug 29;18:380. doi: 10.1186/s12859-017-1807-5 (PMC5576353; doi:10.1186/s12859-017-1807-5)
Supplement: Supplementary file 2 — Table S2. Accuracy of models reconstructed using CONFOLD for the 150 proteins in the PSICOV data set using (a) predicted contacts and (b) contacts and non-contacts and non-contacts. Top-L predicted contacts were considered for all tasks and best-of-20 models are evaluated. For selecting non-contacts confidence thresholds of −2 and −1 were used (results presented in separate columns). (DOCX 27 kb) [file 12859_2017_1807_MOESM2_ESM.docx]

**Table S2**. Accuracy of models reconstructed using CONFOLD for the 150 proteins in the PSICOV data set using (a) predicted contacts and (b) contacts and non-contacts and non-contacts. Top-L predicted contacts were considered for all tasks and best-of-20 models are evaluated. For selecting non-contacts confidence thresholds of -2 and -1 were used (results presented in separate columns).

| **PDB** | **L** | **Fold** | **Contacts Only** | | **Non-contacts at -2 Threshold** | | **Non-contacts  at -1 Threshold** | |
| --- | --- | --- | --- | --- | --- | --- | --- | --- |
|  |  |  | **TM-score** | **RMSD** | **TM-score** | **RMSD** | **TM-score** | **RMSD** |
| 1a3aA | 145 | a+b | 0.51 | 6.6 | 0.52 | 6.3 | 0.56 | 6.4 |
| 1a6mA | 151 | a | 0.42 | 8.1 | 0.47 | 7.7 | 0.49 | 6.9 |
| 1a70A | 97 | a+b | 0.38 | 7.7 | 0.38 | 8.4 | 0.42 | 6.9 |
| 1aapA | 56 | small protein | 0.30 | 6.3 | 0.31 | 6.5 | 0.36 | 6.6 |
| 1abaA | 87 | a/b | 0.49 | 5.1 | 0.47 | 5.3 | 0.54 | 4.7 |
| 1ag6A | 99 | b | 0.33 | 8.1 | 0.36 | 7.2 | 0.38 | 7.6 |
| 1aoeA | 192 | a/b | 0.43 | 9.9 | 0.48 | 9.0 | 0.54 | 8.0 |
| 1atlA | 200 | a+b | 0.27 | 14.0 | 0.28 | 14.2 | 0.27 | 14.0 |
| 1atzA | 75 | a/b | 0.34 | 8.3 | 0.34 | 8.4 | 0.28 | 11.4 |
| 1avsA | 81 | a | 0.50 | 5.9 | 0.51 | 4.9 | 0.51 | 5.0 |
| 1bdoA | 80 | b | 0.47 | 6.1 | 0.47 | 6.3 | 0.47 | 5.9 |
| 1bebA | 156 | b | 0.26 | 12.7 | 0.25 | 15.5 | 0.26 | 13.0 |
| 1behA | 184 | b (cath: a+b) | 0.28 | 12.5 | 0.30 | 12.4 | 0.33 | 11.5 |
| 1bkrA | 108 | a | 0.43 | 7.2 | 0.47 | 6.4 | 0.49 | 5.2 |
| 1brfA | 53 | b | 0.32 | 5.6 | 0.34 | 5.3 | 0.28 | 6.0 |
| 1bsgA | 266 | a+b | 0.59 | 7.3 | 0.63 | 7.0 | 0.60 | 6.7 |
| 1c44A | 123 | a+b | 0.30 | 11.0 | 0.26 | 13.5 | 0.30 | 9.2 |
| 1c52A | 131 | a | 0.48 | 11.7 | 0.44 | 13.0 | 0.45 | 10.3 |
| 1c9oA | 66 | b | 0.39 | 5.7 | 0.37 | 5.0 | 0.40 | 4.9 |
| 1cc8A | 72 | a+b | 0.62 | 5.0 | 0.62 | 4.4 | 0.64 | 5.2 |
| 1chdA | 198 | a/b | 0.61 | 5.5 | 0.62 | 5.9 | 0.64 | 5.5 |
| 1cjwA | 166 | a+b | 0.58 | 9.5 | 0.59 | 9.3 | 0.59 | 9.5 |
| 1ckeA | 212 | a/b | 0.49 | 7.8 | 0.51 | 7.7 | 0.54 | 7.3 |
| 1ctfA | 68 | a+b | 0.40 | 5.4 | 0.38 | 5.5 | 0.43 | 5.1 |
| 1cxyA | 81 | a+b | 0.40 | 7.2 | 0.40 | 7.2 | 0.42 | 6.7 |
| 1cznA | 169 | a/b | 0.45 | 9.4 | 0.46 | 9.7 | 0.47 | 8.8 |
| 1d0qA | 102 | a+b | 0.38 | 8.8 | 0.46 | 6.8 | 0.54 | 6.3 |
| 1d1qA | 159 | a/b | 0.61 | 5.5 | 0.63 | 5.5 | 0.67 | 4.4 |
| 1d4oA | 177 | a/b | 0.30 | 13.6 | 0.29 | 12.2 | 0.29 | 12.4 |
| 1dbxA | 152 | a+b | 0.41 | 7.9 | 0.40 | 8.5 | 0.45 | 8.2 |
| 1dixA | 208 | a+b | 0.27 | 13.2 | 0.30 | 12.7 | 0.30 | 13.1 |
| 1dlwA | 116 | a | 0.41 | 7.2 | 0.42 | 7.8 | 0.48 | 6.2 |
| 1dmgA | 172 | a/b | 0.26 | 12.6 | 0.27 | 17.4 | 0.29 | 14.5 |
| 1dqgA | 134 | b | 0.23 | 13.4 | 0.23 | 15.6 | 0.22 | 16.2 |
| 1dsxA | 87 | a+b | 0.29 | 8.2 | 0.30 | 8.2 | 0.30 | 8.2 |
| 1eazA | 103 | b | 0.65 | 3.7 | 0.67 | 3.5 | 0.64 | 3.8 |
| 1ej0A | 180 | a/b | 0.40 | 8.1 | 0.42 | 7.9 | 0.43 | 8.2 |
| 1ej8A | 140 | b | 0.23 | 18.8 | 0.23 | 21.1 | 0.24 | 20.2 |
| 1ek0A | 168 | a/b | 0.59 | 6.2 | 0.59 | 5.8 | 0.59 | 5.8 |
| 1f6bA | 176 | a/b | 0.51 | 9.4 | 0.52 | 10.0 | 0.55 | 10.6 |
| 1fcyA | 236 | a | 0.23 | 21.8 | 0.26 | 19.1 | 0.30 | 20.2 |
| 1fk5A | 93 | a | 0.25 | 11.0 | 0.24 | 10.1 | 0.26 | 11.1 |
| 1fl0A | 164 | b | 0.30 | 17.5 | 0.29 | 18.1 | 0.31 | 20.1 |
| 1fnaA | 91 | b | 0.52 | 6.4 | 0.56 | 5.6 | 0.56 | 5.5 |
| 1fqtA | 109 | b | 0.63 | 4.7 | 0.59 | 6.0 | 0.59 | 5.7 |
| 1fvgA | 192 | a+b | 0.52 | 16.1 | 0.54 | 16.7 | 0.60 | 13.6 |
| 1fvkA | 188 | a/b | 0.29 | 17.9 | 0.29 | 18.3 | 0.30 | 17.5 |
| 1fx2A | 112 | a+b | 0.31 | 10.8 | 0.36 | 10.7 | 0.37 | 13.3 |
| 1g2rA | 94 | a+b | 0.40 | 7.5 | 0.38 | 7.1 | 0.46 | 6.5 |
| 1g9oA | 91 | b | 0.51 | 5.5 | 0.49 | 5.8 | 0.49 | 6.3 |
| 1gbsA | 185 | a+b | 0.41 | 19.6 | 0.40 | 20.9 | 0.44 | 21.9 |
| 1gmiA | 135 | b | 0.35 | 12.5 | 0.34 | 12.6 | 0.38 | 12.2 |
| 1gmxA | 107 | a/b | 0.58 | 6.9 | 0.58 | 5.2 | 0.60 | 6.8 |
| 1guuA | 50 | a | 0.56 | 4.1 | 0.57 | 3.5 | 0.61 | 3.5 |
| 1gz2A | 138 | a+b | 0.56 | 6.5 | 0.56 | 6.1 | 0.60 | 5.5 |
| 1gzcA | 239 | b | 0.34 | 12.8 | 0.35 | 13.5 | 0.36 | 12.6 |
| 1h0pA | 182 | b | 0.67 | 10.5 | 0.67 | 10.8 | 0.65 | 9.9 |
| 1h2eA | 207 | a/b | 0.71 | 4.8 | 0.71 | 4.8 | 0.71 | 5.3 |
| 1h4xA | 110 | a/b | 0.47 | 7.4 | 0.47 | 7.3 | 0.52 | 6.8 |
| 1h98A | 77 | a+b | 0.37 | 10.1 | 0.34 | 11.3 | 0.35 | 8.6 |
| 1hdoA | 205 | a/b | 0.70 | 5.9 | 0.71 | 7.2 | 0.69 | 6.7 |
| 1hfcA | 157 | a+b | 0.34 | 11.9 | 0.32 | 12.8 | 0.33 | 12.4 |
| 1hh8A | 192 | a | 0.28 | 22.6 | 0.27 | 26.1 | 0.28 | 23.8 |
| 1htwA | 158 | a/b | 0.48 | 8.4 | 0.50 | 7.8 | 0.54 | 6.8 |
| 1hxnA | 210 | b | 0.24 | 35.0 | 0.24 | 35.0 | 0.26 | 29.3 |
| 1i1jA | 106 | b | 0.21 | 13.0 | 0.21 | 14.6 | 0.21 | 13.2 |
| 1i1nA | 224 | a/b | 0.61 | 6.3 | 0.61 | 6.4 | 0.59 | 6.2 |
| 1i4jA | 110 | a+b | 0.30 | 13.4 | 0.34 | 12.3 | 0.33 | 12.9 |
| 1i58A | 189 | a+b | 0.44 | 10.9 | 0.45 | 11.0 | 0.45 | 10.9 |
| 1i5gA | 144 | a/b | 0.44 | 9.9 | 0.47 | 8.4 | 0.48 | 8.5 |
| 1i71A | 83 | b | 0.31 | 9.6 | 0.31 | 8.9 | 0.36 | 7.8 |
| 1ihzA | 136 | b | 0.53 | 6.7 | 0.53 | 5.9 | 0.55 | 7.1 |
| 1iibA | 103 | a/b | 0.48 | 5.6 | 0.49 | 5.9 | 0.52 | 6.0 |
| 1im5A | 179 | a/b | 0.49 | 7.4 | 0.46 | 7.7 | 0.50 | 7.1 |
| 1iwdA | 215 | a+b | 0.61 | 6.2 | 0.65 | 5.9 | 0.55 | 7.8 |
| 1j3aA | 129 | a/b | 0.37 | 10.6 | 0.39 | 10.1 | 0.37 | 10.7 |
| 1jbeA | 126 | a/b | 0.75 | 3.3 | 0.74 | 3.3 | 0.74 | 3.2 |
| 1jbkA | 189 | a/b | 0.31 | 13.3 | 0.29 | 13.1 | 0.29 | 14.8 |
| 1jfuA | 176 | a/b | 0.52 | 14.2 | 0.55 | 12.8 | 0.57 | 14.4 |
| 1jfxA | 217 | a/b | 0.56 | 6.4 | 0.56 | 7.1 | 0.58 | 5.9 |
| 1jkxA | 209 | a/b | 0.60 | 8.8 | 0.58 | 8.8 | 0.58 | 8.4 |
| 1jl1A | 152 | a/b | 0.59 | 7.9 | 0.61 | 8.5 | 0.60 | 7.8 |
| 1jo0A | 97 | a+b | 0.38 | 13.1 | 0.37 | 12.5 | 0.37 | 12.7 |
| 1jo8A | 58 | b | 0.48 | 4.2 | 0.45 | 4.1 | 0.43 | 4.3 |
| 1josA | 100 | a+b | 0.43 | 6.6 | 0.42 | 7.5 | 0.47 | 6.6 |
| 1jvwA | 160 | a+b | 0.44 | 9.7 | 0.46 | 10.9 | 0.51 | 11.0 |
| 1jwqA | 179 | a/b | 0.74 | 3.5 | 0.75 | 3.4 | 0.74 | 3.5 |
| 1jyhA | 155 | a+b | 0.27 | 13.7 | 0.28 | 11.7 | 0.27 | 14.4 |
| 1k6kA | 142 | a | 0.34 | 9.9 | 0.34 | 10.0 | 0.37 | 8.4 |
| 1k7cA | 233 | a/b | 0.40 | 13.5 | 0.44 | 12.2 | 0.47 | 12.2 |
| 1k7jA | 206 | a+b | 0.42 | 12.4 | 0.43 | 11.3 | 0.46 | 10.7 |
| 1kidA | 193 | a/b | 0.32 | 11.7 | 0.30 | 11.7 | 0.31 | 10.9 |
| 1kq6A | 140 | a+b | 0.26 | 15.7 | 0.24 | 16.7 | 0.31 | 18.6 |
| 1kqrA | 160 | b | 0.22 | 14.0 | 0.21 | 14.4 | 0.21 | 14.8 |
| 1ktgA | 137 | a+b | 0.51 | 11.0 | 0.48 | 11.2 | 0.49 | 10.9 |
| 1ku3A | 61 | a | 0.51 | 3.7 | 0.54 | 4.4 | 0.57 | 3.8 |
| 1kw4A | 70 | a | 0.36 | 7.8 | 0.34 | 7.6 | 0.40 | 6.6 |
| 1lm4A | 189 | a+b | 0.55 | 9.2 | 0.56 | 9.0 | 0.56 | 8.7 |
| 1lo7A | 140 | a+b | 0.42 | 9.0 | 0.43 | 9.6 | 0.43 | 9.8 |
| 1lpyA | 162 | a+b /a | 0.37 | 9.8 | 0.38 | 10.0 | 0.43 | 9.2 |
| 1m4jA | 133 | a+b | 0.25 | 10.5 | 0.25 | 10.2 | 0.30 | 9.4 |
| 1m8aA | 61 | a+b /b | 0.35 | 8.3 | 0.34 | 7.5 | 0.34 | 7.9 |
| 1mk0A | 97 | a+b | 0.28 | 11.5 | 0.29 | 11.2 | 0.29 | 10.1 |
| 1mugA | 165 | a/b | 0.31 | 12.5 | 0.31 | 12.7 | 0.30 | 12.9 |
| 1nb9A | 147 | b | 0.49 | 13.1 | 0.49 | 13.5 | 0.51 | 12.7 |
| 1ne2A | 176 | a/b | 0.33 | 15.6 | 0.37 | 15.2 | 0.35 | 14.7 |
| 1npsA | 88 | b | 0.35 | 7.3 | 0.36 | 7.1 | 0.38 | 7.3 |
| 1nrvA | 100 | a+b | 0.42 | 6.7 | 0.41 | 6.9 | 0.44 | 5.9 |
| 1ny1A | 235 | a/b | 0.47 | 23.5 | 0.47 | 19.6 | 0.52 | 20.2 |
| 1o1zA | 226 | a/b | 0.51 | 10.1 | 0.54 | 9.1 | 0.55 | 9.6 |
| 1p90A | 123 | a/b | 0.40 | 8.6 | 0.42 | 7.2 | 0.45 | 8.0 |
| 1pchA | 88 | a+b | 0.58 | 4.2 | 0.60 | 4.2 | 0.56 | 4.3 |
| 1pkoA | 124 | b | 0.39 | 10.6 | 0.38 | 11.5 | 0.38 | 11.2 |
| 1qf9A | 194 | a/b | 0.57 | 6.5 | 0.58 | 6.7 | 0.60 | 6.9 |
| 1qjpA | 137 | b | 0.33 | 11.2 | 0.37 | 11.2 | 0.36 | 10.6 |
| 1ql0A | 241 | a+b | 0.29 | 16.2 | 0.32 | 15.2 | 0.34 | 14.9 |
| 1r26A | 113 | a/b | 0.63 | 7.2 | 0.64 | 5.0 | 0.68 | 7.5 |
| 1roaA | 111 | a+b | 0.28 | 11.4 | 0.30 | 11.3 | 0.25 | 11.3 |
| 1rw1A | 114 | a/b | 0.36 | 8.8 | 0.38 | 8.7 | 0.40 | 7.7 |
| 1rw7A | 235 | a/b | 0.46 | 10.1 | 0.43 | 10.9 | 0.45 | 10.0 |
| 1rybA | 186 | a/b | 0.57 | 6.0 | 0.58 | 6.6 | 0.63 | 5.5 |
| 1smxA | 87 | b | 0.26 | 10.2 | 0.30 | 10.8 | 0.25 | 11.3 |
| 1svyA | 101 | a+b | 0.31 | 9.2 | 0.35 | 9.8 | 0.36 | 8.9 |
| 1t8kA | 77 | a | 0.59 | 4.0 | 0.58 | 4.2 | 0.62 | 3.8 |
| 1tifA | 76 | a+b | 0.37 | 7.9 | 0.36 | 7.6 | 0.37 | 7.3 |
| 1tqgA | 105 | a | 0.56 | 5.7 | 0.55 | 6.0 | 0.58 | 5.6 |
| 1tqhA | 242 | a/b | 0.63 | 13.9 | 0.64 | 12.7 | 0.63 | 12.0 |
| 1tzvA | 141 | a | 0.60 | 5.0 | 0.58 | 5.5 | 0.68 | 4.5 |
| 1vfyA | 67 | a+b | 0.31 | 7.6 | 0.31 | 7.6 | 0.29 | 8.7 |
| 1vhuA | 192 | a/b | 0.54 | 10.3 | 0.59 | 8.9 | 0.58 | 8.4 |
| 1vjkA | 87 | a+b | 0.46 | 5.8 | 0.45 | 5.7 | 0.50 | 6.0 |
| 1vmbA | 107 | a+b | 0.32 | 9.1 | 0.33 | 8.4 | 0.34 | 8.1 |
| 1vp6A | 133 | b | 0.48 | 7.2 | 0.48 | 7.2 | 0.49 | 7.4 |
| 1w0hA | 200 | a/b | 0.65 | 9.4 | 0.67 | 9.0 | 0.68 | 8.4 |
| 1whiA | 122 | b | 0.34 | 10.7 | 0.33 | 11.0 | 0.31 | 10.6 |
| 1wjxA | 112 | b | 0.40 | 7.1 | 0.44 | 6.4 | 0.45 | 6.5 |
| 1wkcA | 168 | a/b | 0.56 | 5.9 | 0.59 | 5.6 | 0.59 | 5.6 |
| 1xdzA | 238 | a/b | 0.44 | 9.7 | 0.43 | 9.8 | 0.49 | 8.9 |
| 1xffA | 238 | a+b | 0.64 | 6.4 | 0.60 | 6.7 | 0.59 | 7.0 |
| 1xkrA | 205 | a+b | 0.29 | 12.4 | 0.31 | 11.8 | 0.30 | 11.9 |
| 2arcA | 161 | b | 0.24 | 19.0 | 0.26 | 17.4 | 0.24 | 16.8 |
| 2cuaA | 122 | b | 0.41 | 17.8 | 0.34 | 19.4 | 0.43 | 13.4 |
| 2hs1A | 99 | b | 0.31 | 11.1 | 0.31 | 11.0 | 0.36 | 9.9 |
| 2mhrA | 118 | a | 0.35 | 10.2 | 0.36 | 10.8 | 0.41 | 10.5 |
| 2phyA | 125 | a+b | 0.33 | 20.6 | 0.37 | 20.1 | 0.39 | 18.7 |
| 2tpsA | 226 | a/b | 0.67 | 6.3 | 0.69 | 6.8 | 0.70 | 5.9 |
| 2vxnA | 249 | a+b | 0.61 | 6.7 | 0.61 | 6.8 | 0.65 | 6.2 |
| 3borA | 194 | a+b | 0.62 | 5.2 | 0.63 | 5.1 | 0.65 | 5.0 |
| 3dqgA | 148 | b | 0.32 | 12.1 | 0.35 | 12.1 | 0.35 | 12.1 |
| 5ptpA | 222 | b | 0.77 | 3.5 | 0.77 | 3.5 | 0.78 | 3.5 |
| **Average** | | | **0.43** | **9.8** | **0.44** | **9.8** | **0.46** | **9.5** |
