# Supplementary material for: Improved protein structure reconstruction using secondary structures, contacts at higher distance thresholds, and non-contacts
Source: BMC Bioinformatics. 2017 Aug 29;18:380. doi: 10.1186/s12859-017-1807-5 (PMC5576353; doi:10.1186/s12859-017-1807-5)
Supplement: Supplementary file 3 — Table S3. Comparison of reconstruction using true contacts and secondary structures vs predicted contacts and secondary structures for the 496 CASP structural domains. The columns L, H, E, and Nc refer to the length of the domain, number of helical residues in the native structure, number of strand residues in the native structure, and the number of contacts in the native structure, respectively. TM-score and RMSD for the best of 20 model reconstructed using true contacts and best of 200 models predicted using predicted contacts are reported. Precision of top L/5, L/2, L, and 2 L contacts are reported when all contacts are evaluated and when only long-range contacts are evaluated. (DOCX 99 kb) [file 12859_2017_1807_MOESM3_ESM.docx]

**Table S3**. Comparison of reconstruction using true contacts and secondary structures vs predicted contacts and secondary structures for the 496 CASP structural domains. The columns L, H, E, and N_c_ refer to the length of the domain, number of helical residues in the native structure, number of strand residues in the native structure, and the number of contacts in the native structure, respectively. TM-score and RMSD for the best of 20 model reconstructed using true contacts and best of 200 models predicted using predicted contacts are reported. Precision of top L/5, L/2, L, and 2L contacts are reported when all contacts are evaluated and when only long-range contacts are evaluated.

| **CASP** | **Domain** | **L** | **Type** | **Native** | | | **Using True**  **Contacts and SS** | | **Using Predicted**  **Contacts and SS** | | **Precision of Predicted Contacts**  **(Long-range)** | | | | **Precision of Predicted All**  **Contacts (Short-, Medium & Long)** | | | |
| --- | --- | --- | --- | --- | --- | --- | --- | --- | --- | --- | --- | --- | --- | --- | --- | --- | --- | --- |
|  |  |  |  |  |  |  |  |  |  |  |  |  |  |  |  |  |  |  |
|  |  |  |  | **H** | **E** | **N_c_** | **TM-score** | **RMSD** | **TM-score** | **RMSD** | **Top-L/5** | **Top-L/2** | **Top-L** | **Top-2L** | **Top-L/5** | **Top-L/2** | **Top-L** | **Top-2L** |
| 8 | T0388-D1 | 164 | TBM-HA | 54 | 29 | 351 | 0.94 | 1.3 | 0.69 | 3.8 | 100.0 | 97.0 | 87.8 | 70.1 | 100.0 | 100.0 | 91.5 | 78.7 |
| 8 | T0389-D1 | 134 | TBM | 49 | 23 | 254 | 0.92 | 1.3 | 0.60 | 7.1 | 100.0 | 88.9 | 83.6 | 61.9 | 100.0 | 88.9 | 83.6 | 71.6 |
| 8 | T0390-D1 | 124 | TBM-HA | 6 | 44 | 271 | 0.86 | 2.0 | 0.33 | 12.7 | 41.7 | 44.0 | 30.7 | 21.8 | 66.7 | 44.0 | 40.3 | 39.5 |
| 8 | T0391-D1 | 128 | TBM | 7 | 63 | 316 | 0.84 | 2.1 | 0.61 | 6.7 | 76.9 | 80.8 | 75.0 | 52.3 | 84.6 | 84.6 | 87.5 | 82.0 |
| 8 | T0392-D1 | 82 | TBM-HA | 13 | 27 | 173 | 0.85 | 1.6 | 0.53 | 5.2 | 100.0 | 100.0 | 80.5 | 59.8 | 75.0 | 75.0 | 61.0 | 57.3 |
|  | T0393-D1 | 159 | TBM | 46 | 35 | 378 | 0.94 | 1.3 | 0.75 | 4.1 | 87.5 | 90.6 | 77.5 | 54.1 | 93.8 | 90.6 | 83.8 | 68.6 |
| 8 | T0393-D2 | 99 | TBM | 74 | 0 | 50 | 0.72 | 2.9 | 0.44 | 14.4 | 0.0 | 10.0 | 6.0 | 4.0 | 40.0 | 25.0 | 24.0 | 22.2 |
| 8 | T0394-D1 | 238 | TBM | 89 | 39 | 560 | 0.91 | 2.0 | 0.67 | 7.6 | 95.8 | 93.8 | 88.2 | 73.5 | 95.8 | 93.8 | 87.4 | 79.0 |
| 8 | T0395-D1 | 218 | TBM | 88 | 23 | 408 | 0.90 | 2.2 | 0.62 | 8.7 | 95.5 | 81.8 | 77.1 | 60.1 | 95.5 | 81.8 | 77.1 | 66.1 |
| 8 | T0396-D1 | 102 | TBM-HA | 71 | 0 | 101 | 0.91 | 1.4 | 0.62 | 5.1 | 70.0 | 70.0 | 47.1 | 34.3 | 80.0 | 60.0 | 56.9 | 41.2 |
| 8 | T0397-D1 | 82 | FM | 0 | 32 | 151 | 0.76 | 2.5 | 0.29 | 11.6 | 0.0 | 0.0 | 14.6 | 20.7 | 100.0 | 87.5 | 63.4 | 40.2 |
| 8 | T0397-D2 | 68 | TBM | 0 | 43 | 170 | 0.81 | 1.8 | 0.44 | 5.2 | 85.7 | 64.3 | 55.9 | 44.1 | 100.0 | 85.7 | 88.2 | 83.8 |
| 8 | T0398-D1 | 143 | TBM-HA | 43 | 25 | 338 | 0.91 | 1.8 | 0.40 | 10.0 | 78.6 | 55.2 | 45.8 | 31.5 | 78.6 | 86.2 | 63.9 | 53.9 |
| 8 | T0398-D2 | 147 | TBM-HA | 63 | 27 | 341 | 0.94 | 1.2 | 0.36 | 10.5 | 80.0 | 62.1 | 40.5 | 26.5 | 53.3 | 31.0 | 27.0 | 28.6 |
| 8 | T0399-D1 | 162 | TBM | 29 | 61 | 360 | 0.88 | 2.0 | 0.45 | 9.1 | 50.0 | 31.3 | 21.0 | 16.7 | 100.0 | 96.9 | 85.2 | 67.9 |
| 8 | T0400-D1 | 155 | TBM-HA | 40 | 43 | 300 | 0.86 | 2.1 | 0.38 | 10.5 | 43.8 | 22.6 | 12.8 | 6.5 | 100.0 | 80.7 | 52.6 | 40.0 |
| 8 | T0401-D1 | 127 | TBM | 43 | 36 | 247 | 0.90 | 1.5 | 0.70 | 4.4 | 61.5 | 56.0 | 46.9 | 36.2 | 100.0 | 96.0 | 87.5 | 69.3 |
| 8 | T0402-D1 | 114 | TBM-HA | 30 | 56 | 254 | 0.88 | 1.7 | 0.65 | 4.1 | 100.0 | 100.0 | 84.2 | 70.2 | 100.0 | 100.0 | 100.0 | 88.6 |
| 8 | T0404-D1 | 79 | TBM-HA | 26 | 32 | 154 | 0.88 | 1.5 | 0.76 | 2.4 | 100.0 | 100.0 | 90.0 | 81.0 | 100.0 | 100.0 | 97.5 | 86.1 |
| 8 | T0405-D1 | 72 | FM | 58 | 0 | 67 | 0.82 | 1.9 | 0.49 | 4.5 | 57.1 | 28.6 | 13.9 | 15.3 | 28.6 | 50.0 | 25.0 | 22.2 |
| 8 | T0405-D2 | 208 | FM | 77 | 50 | 396 | 0.90 | 1.9 | 0.32 | 12.5 | 23.8 | 19.1 | 16.4 | 12.0 | 81.0 | 64.3 | 37.5 | 26.4 |
| 8 | T0406-D1 | 147 | TBM | 84 | 8 | 233 | 0.92 | 1.6 | 0.74 | 4.2 | 86.7 | 82.8 | 63.5 | 46.9 | 86.7 | 86.2 | 74.3 | 53.7 |
| 8 | T0407-D1 | 231 | TBM | 63 | 54 | 588 | 0.94 | 1.4 | 0.59 | 7.4 | 95.7 | 93.5 | 81.9 | 64.5 | 95.7 | 95.7 | 89.7 | 80.5 |
| 8 | T0407-D2 | 97 | TBM | 8 | 57 | 211 | 0.85 | 1.7 | 0.36 | 9.6 | 40.0 | 31.6 | 24.5 | 21.7 | 40.0 | 36.8 | 28.6 | 29.9 |
| 8 | T0408-D1 | 98 | TBM | 79 | 0 | 90 | 0.83 | 2.1 | 0.60 | 15.0 | 90.0 | 65.0 | 44.9 | 28.6 | 90.0 | 85.0 | 71.4 | 50.0 |
| 8 | T0409-D1 | 62 | TBM | 0 | 29 | 136 | 0.80 | 1.7 | 0.72 | 2.6 | 100.0 | 100.0 | 77.4 | 67.7 | 100.0 | 100.0 | 87.1 | 80.7 |
| 8 | T0411-D1 | 118 | TBM | 42 | 17 | 231 | 0.91 | 1.3 | 0.32 | 12.5 | 16.7 | 8.3 | 17.0 | 11.9 | 50.0 | 33.3 | 22.0 | 17.8 |
| 8 | T0412-D1 | 165 | TBM | 73 | 43 | 326 | 0.93 | 1.4 | 0.78 | 3.4 | 88.2 | 87.9 | 71.1 | 57.6 | 100.0 | 93.9 | 85.5 | 77.0 |
| 8 | T0413-D1 | 282 | TBM | 101 | 46 | 684 | 0.95 | 1.5 | 0.39 | 17.4 | 85.7 | 69.6 | 53.9 | 30.1 | 75.0 | 62.5 | 43.3 | 34.0 |
| 8 | T0414-D1 | 127 | TBM | 14 | 63 | 295 | 0.88 | 1.8 | 0.47 | 9.7 | 92.3 | 88.0 | 78.1 | 64.6 | 100.0 | 84.0 | 82.8 | 68.5 |
| 8 | T0415-D1 | 107 | TBM | 15 | 50 | 215 | 0.86 | 1.7 | 0.60 | 9.7 | 63.6 | 52.4 | 48.2 | 33.6 | 81.8 | 85.7 | 87.0 | 79.4 |
| 8 | T0416-D1 | 232 | TBM-HA | 110 | 30 | 429 | 0.94 | 1.5 | 0.48 | 12.5 | 87.0 | 73.9 | 65.5 | 50.0 | 87.0 | 73.9 | 69.0 | 53.5 |
| 8 | T0417-D1 | 153 | TBM | 42 | 46 | 308 | 0.90 | 2.1 | 0.34 | 12.2 | 46.7 | 22.6 | 9.1 | 5.2 | 80.0 | 61.3 | 36.4 | 30.1 |
| 8 | T0418-D1 | 141 | TBM-HA | 39 | 25 | 311 | 0.91 | 1.5 | 0.31 | 15.6 | 92.9 | 57.1 | 28.2 | 15.6 | 42.9 | 50.0 | 32.4 | 23.4 |
| 8 | T0418-D2 | 69 | TBM-HA | 46 | 0 | 80 | 0.87 | 1.2 | 0.71 | 2.8 | 71.4 | 35.7 | 42.9 | 30.4 | 71.4 | 64.3 | 42.9 | 40.6 |
| 8 | T0419-D1 | 224 | TBM | 121 | 30 | 395 | 0.92 | 1.8 | 0.59 | 8.6 | 77.3 | 77.8 | 67.9 | 50.5 | 77.3 | 77.8 | 67.9 | 53.6 |
| 8 | T0419-D2 | 241 | TBM | 117 | 31 | 419 | 0.91 | 2.0 | 0.53 | 10.3 | 87.5 | 83.3 | 64.5 | 49.0 | 87.5 | 83.3 | 66.9 | 52.7 |
| 8 | T0420-D1 | 168 | TBM | 45 | 35 | 399 | 0.93 | 1.4 | 0.68 | 4.0 | 100.0 | 82.4 | 71.4 | 54.8 | 100.0 | 91.2 | 76.2 | 69.1 |
| 8 | T0421-D1 | 221 | TBM | 116 | 31 | 355 | 0.89 | 2.3 | 0.56 | 7.5 | 81.8 | 77.3 | 69.4 | 47.5 | 81.8 | 77.3 | 69.4 | 52.0 |
| 8 | T0422-D1 | 200 | TBM | 84 | 28 | 389 | 0.91 | 1.9 | 0.24 | 17.8 | 55.0 | 40.0 | 16.0 | 10.5 | 25.0 | 25.0 | 19.0 | 16.5 |
| 8 | T0422-D2 | 80 | TBM-HA | 51 | 0 | 107 | 0.89 | 1.6 | 0.39 | 11.2 | 0.0 | 0.0 | 10.0 | 10.0 | 0.0 | 6.3 | 12.5 | 10.0 |
| 8 | T0423-D1 | 147 | TBM-HA | 48 | 43 | 376 | 0.92 | 1.6 | 0.59 | 9.7 | 100.0 | 89.7 | 77.0 | 70.1 | 100.0 | 89.7 | 79.7 | 70.1 |
| 8 | T0424-D1 | 175 | TBM | 0 | 100 | 425 | 0.68 | 4.4 | 0.29 | 16.1 | 72.2 | 57.1 | 34.1 | 24.6 | 77.8 | 68.6 | 60.2 | 41.1 |
| 8 | T0424-D2 | 84 | TBM | 26 | 9 | 173 | 0.92 | 1.0 | 0.58 | 4.8 | 50.0 | 52.9 | 50.0 | 45.2 | 87.5 | 88.2 | 69.1 | 57.1 |
| 8 | T0425-D1 | 179 | TBM | 75 | 28 | 420 | 0.93 | 1.4 | 0.76 | 4.2 | 94.4 | 91.7 | 83.3 | 69.3 | 94.4 | 91.7 | 83.3 | 72.6 |
| 8 | T0426-D1 | 257 | TBM-HA | 24 | 76 | 684 | 0.93 | 1.7 | 0.48 | 11.5 | 100.0 | 94.1 | 79.1 | 61.5 | 96.2 | 92.2 | 80.6 | 70.4 |
| 8 | T0427-D1 | 218 | TBM | 99 | 55 | 427 | 0.93 | 1.7 | 0.74 | 4.1 | 100.0 | 100.0 | 88.1 | 76.2 | 100.0 | 95.5 | 88.1 | 77.5 |
| 8 | T0427-D2 | 184 | TBM | 83 | 42 | 334 | 0.90 | 1.9 | 0.71 | 8.8 | 94.4 | 94.6 | 84.8 | 64.7 | 94.4 | 97.3 | 91.3 | 75.0 |
| 8 | T0428-D1 | 229 | TBM-HA | 82 | 30 | 508 | 0.94 | 1.6 | 0.61 | 7.4 | 91.3 | 89.1 | 80.0 | 68.1 | 91.3 | 89.1 | 85.2 | 73.4 |
| 8 | T0429-D2 | 75 | TBM | 0 | 33 | 163 | 0.81 | 2.1 | 0.27 | 10.6 | 12.5 | 6.7 | 21.1 | 20.0 | 87.5 | 66.7 | 55.3 | 48.0 |
| 8 | T0430-D1 | 138 | TBM | 38 | 40 | 276 | 0.86 | 2.2 | 0.28 | 13.8 | 21.4 | 21.4 | 10.1 | 7.3 | 35.7 | 50.0 | 43.5 | 31.9 |
| 8 | T0430-D2 | 189 | TBM | 74 | 28 | 349 | 0.91 | 1.7 | 0.33 | 14.5 | 79.0 | 60.5 | 25.3 | 15.9 | 57.9 | 47.4 | 33.7 | 25.4 |
| 8 | T0431-D1 | 101 | TBM | 16 | 34 | 187 | 0.76 | 3.2 | 0.33 | 10.8 | 50.0 | 50.0 | 27.5 | 16.8 | 100.0 | 90.0 | 62.8 | 39.6 |
| 8 | T0431-D2 | 357 | TBM | 196 | 27 | 626 | 0.93 | 2.0 | 0.55 | 12.4 | 88.9 | 83.1 | 62.0 | 44.3 | 88.9 | 87.3 | 67.6 | 49.6 |
| 8 | T0432-D1 | 130 | TBM-HA | 90 | 0 | 154 | 0.92 | 1.8 | 0.76 | 3.6 | 84.6 | 84.6 | 61.5 | 43.9 | 92.3 | 88.5 | 76.9 | 58.5 |
| 8 | T0433-D1 | 199 | TBM | 72 | 40 | 476 | 0.94 | 1.4 | 0.38 | 13.2 | 55.0 | 47.5 | 28.0 | 21.1 | 40.0 | 37.5 | 31.0 | 24.1 |
| 8 | T0434-D1 | 151 | TBM | 17 | 48 | 349 | 0.91 | 1.6 | 0.45 | 14.5 | 93.3 | 90.0 | 77.6 | 55.6 | 100.0 | 96.7 | 90.8 | 76.8 |
| 8 | T0435-D1 | 118 | TBM-HA | 11 | 45 | 263 | 0.86 | 1.9 | 0.47 | 10.3 | 91.7 | 91.7 | 83.1 | 65.3 | 100.0 | 95.8 | 91.5 | 83.9 |
| 8 | T0436-D1 | 405 | TBM | 162 | 56 | 957 | 0.96 | 1.5 | 0.34 | 20.2 | 82.9 | 69.1 | 45.3 | 28.6 | 80.5 | 67.9 | 51.7 | 40.0 |
| 8 | T0437-D1 | 68 | TBM-HA | 31 | 24 | 121 | 0.84 | 1.4 | 0.50 | 4.6 | 71.4 | 78.6 | 44.1 | 32.4 | 71.4 | 85.7 | 70.6 | 64.7 |
| 8 | T0438-D1 | 164 | TBM-HA | 36 | 37 | 415 | 0.93 | 1.7 | 0.68 | 6.0 | 100.0 | 100.0 | 93.9 | 75.0 | 100.0 | 100.0 | 93.9 | 76.8 |
| 8 | T0438-D2 | 223 | TBM-HA | 74 | 39 | 547 | 0.85 | 2.6 | 0.54 | 8.6 | 95.5 | 84.4 | 78.6 | 57.0 | 95.5 | 93.3 | 80.4 | 71.3 |
| 8 | T0440-D1 | 275 | TBM | 104 | 45 | 636 | 0.95 | 1.4 | 0.62 | 7.1 | 92.9 | 92.7 | 84.8 | 64.7 | 92.9 | 92.7 | 86.2 | 68.4 |
| 8 | T0441-D1 | 124 | TBM | 54 | 21 | 241 | 0.91 | 1.5 | 0.58 | 9.0 | 91.7 | 96.0 | 72.6 | 54.0 | 83.3 | 92.0 | 82.3 | 73.4 |
| 8 | T0441-D2 | 146 | TBM-HA | 62 | 24 | 361 | 0.94 | 1.2 | 0.78 | 3.1 | 100.0 | 96.6 | 89.0 | 78.8 | 100.0 | 100.0 | 89.0 | 82.2 |
| 8 | T0442-D1 | 157 | TBM-HA | 33 | 34 | 379 | 0.85 | 2.6 | 0.43 | 16.8 | 87.5 | 71.0 | 51.9 | 35.0 | 100.0 | 87.1 | 67.1 | 51.0 |
| 8 | T0442-D2 | 73 | TBM-HA | 8 | 19 | 123 | 0.72 | 3.2 | 0.48 | 5.1 | 85.7 | 53.3 | 46.0 | 31.5 | 85.7 | 66.7 | 54.1 | 46.6 |
| 8 | T0443-D1 | 66 | TBM-HA | 41 | 0 | 42 | 0.81 | 2.0 | 0.66 | 2.8 | 57.1 | 38.5 | 33.3 | 24.6 | 57.1 | 50.0 | 46.9 | 34.9 |
| 8 | T0443-D2 | 60 | FM | 18 | 22 | 106 | 0.84 | 1.4 | 0.29 | 10.4 | 0.0 | 0.0 | 0.0 | 0.0 | 0.0 | 25.0 | 26.7 | 23.3 |
| 8 | T0443-D3 | 66 | TBM | 35 | 6 | 67 | 0.73 | 2.7 | 0.38 | 10.1 | 28.6 | 30.8 | 15.2 | 13.6 | 42.9 | 30.8 | 30.3 | 19.7 |
| 8 | T0444-D1 | 276 | TBM-HA | 184 | 0 | 419 | 0.94 | 1.5 | 0.64 | 10.5 | 67.9 | 61.8 | 54.4 | 38.4 | 75.0 | 69.1 | 55.8 | 44.6 |
| 8 | T0445-D1 | 155 | TBM-HA | 50 | 33 | 369 | 0.93 | 1.4 | 0.29 | 12.1 | 56.3 | 35.5 | 15.4 | 7.7 | 75.0 | 58.1 | 41.0 | 28.4 |
| 8 | T0445-D2 | 107 | TBM | 32 | 28 | 206 | 0.89 | 1.6 | 0.62 | 4.1 | 100.0 | 81.0 | 59.3 | 43.9 | 100.0 | 85.7 | 81.5 | 68.2 |
| 8 | T0447-D1 | 542 | TBM-HA | 183 | 109 | 1439 | 0.96 | 1.7 | 0.67 | 9.1 | 94.4 | 88.0 | 70.5 | 56.3 | 98.2 | 90.7 | 77.5 | 67.7 |
| 8 | T0448-D1 | 207 | TBM | 77 | 22 | 490 | 0.94 | 1.3 | 0.52 | 7.1 | 66.7 | 56.1 | 53.9 | 45.4 | 47.6 | 39.0 | 36.5 | 30.9 |
| 8 | T0449-D1 | 296 | TBM | 6 | 133 | 844 | 0.94 | 1.7 | 0.62 | 6.4 | 93.3 | 91.5 | 83.8 | 66.6 | 100.0 | 100.0 | 92.6 | 80.4 |
| 8 | T0450-D1 | 491 | TBM-HA | 186 | 116 | 1219 | 0.96 | 1.8 | 0.41 | 21.2 | 95.9 | 92.9 | 65.9 | 49.9 | 89.8 | 88.8 | 81.7 | 63.1 |
| 8 | T0451-D1 | 127 | TBM | 36 | 59 | 277 | 0.91 | 1.4 | 0.30 | 25.5 | 69.2 | 56.0 | 32.8 | 18.1 | 76.9 | 88.0 | 82.8 | 70.1 |
| 8 | T0452-D1 | 156 | TBM-HA | 79 | 26 | 359 | 0.92 | 1.6 | 0.46 | 11.2 | 50.0 | 58.1 | 56.4 | 38.5 | 56.3 | 51.6 | 46.2 | 42.3 |
| 8 | T0452-D2 | 163 | TBM-HA | 53 | 40 | 368 | 0.92 | 1.5 | 0.68 | 4.0 | 93.8 | 97.0 | 81.7 | 65.6 | 100.0 | 93.9 | 92.7 | 80.4 |
| 8 | T0453-D1 | 86 | TBM-HA | 16 | 29 | 159 | 0.86 | 1.6 | 0.69 | 2.9 | 100.0 | 94.1 | 81.4 | 57.0 | 100.0 | 100.0 | 92.7 | 80.5 |
| 8 | T0454-D2 | 140 | TBM | 94 | 0 | 141 | 0.88 | 2.1 | 0.58 | 7.6 | 64.3 | 64.3 | 42.9 | 28.6 | 78.6 | 71.4 | 55.7 | 37.9 |
| 8 | T0455-D1 | 139 | TBM-HA | 29 | 63 | 305 | 0.87 | 3.3 | 0.57 | 5.6 | 78.6 | 82.1 | 78.6 | 57.6 | 100.0 | 92.9 | 92.9 | 87.8 |
| 8 | T0456-D1 | 87 | TBM | 18 | 36 | 152 | 0.82 | 1.9 | 0.29 | 13.7 | 0.0 | 0.0 | 0.0 | 2.3 | 88.9 | 88.2 | 61.4 | 52.9 |
| 8 | T0456-D2 | 175 | TBM-HA | 89 | 4 | 299 | 0.93 | 1.5 | 0.39 | 15.3 | 72.2 | 45.7 | 27.3 | 16.0 | 66.7 | 42.9 | 34.1 | 25.1 |
| 8 | T0457-D1 | 194 | TBM | 72 | 23 | 431 | 0.94 | 1.4 | 0.73 | 4.2 | 89.5 | 87.2 | 77.3 | 56.7 | 89.5 | 84.6 | 82.5 | 66.0 |
| 8 | T0457-D2 | 118 | TBM | 39 | 29 | 240 | 0.90 | 1.6 | 0.62 | 4.6 | 100.0 | 87.5 | 67.8 | 44.1 | 91.7 | 95.8 | 88.1 | 74.6 |
| 8 | T0458-D1 | 77 | TBM-HA | 27 | 27 | 141 | 0.88 | 1.3 | 0.55 | 5.2 | 100.0 | 100.0 | 87.2 | 66.2 | 75.0 | 66.7 | 66.7 | 61.0 |
| 8 | T0459-D1 | 91 | TBM-HA | 54 | 10 | 117 | 0.87 | 1.8 | 0.38 | 10.1 | 22.2 | 11.1 | 8.7 | 4.4 | 22.2 | 22.2 | 15.2 | 15.4 |
| 8 | T0460-D1 | 80 | TBM-HA | 32 | 22 | 116 | 0.82 | 1.8 | 0.32 | 10.5 | 12.5 | 6.3 | 5.0 | 2.5 | 25.0 | 18.8 | 20.0 | 17.5 |
| 8 | T0461-D1 | 154 | TBM-HA | 64 | 29 | 351 | 0.92 | 1.5 | 0.79 | 3.1 | 100.0 | 90.3 | 77.9 | 63.0 | 86.7 | 87.1 | 87.0 | 80.5 |
| 8 | T0462-D1 | 70 | TBM | 11 | 29 | 149 | 0.84 | 1.5 | 0.70 | 2.7 | 100.0 | 92.9 | 91.4 | 71.4 | 100.0 | 100.0 | 97.1 | 85.7 |
| 8 | T0462-D2 | 63 | TBM | 10 | 21 | 113 | 0.83 | 1.8 | 0.43 | 6.6 | 100.0 | 92.3 | 75.0 | 69.8 | 100.0 | 92.3 | 68.8 | 65.1 |
| 8 | T0463-D1 | 211 | TBM | 61 | 49 | 493 | 0.93 | 1.8 | 0.56 | 8.8 | 95.2 | 85.7 | 73.6 | 52.1 | 71.4 | 59.5 | 44.3 | 40.3 |
| 8 | T0464-D1 | 69 | TBM | 13 | 18 | 126 | 0.85 | 1.4 | 0.31 | 12.0 | 0.0 | 7.1 | 14.3 | 13.0 | 85.7 | 78.6 | 48.6 | 31.9 |
| 8 | T0465-D1 | 96 | FM | 51 | 9 | 129 | 0.85 | 2.0 | 0.52 | 6.3 | 90.0 | 73.7 | 47.9 | 32.3 | 100.0 | 94.7 | 70.8 | 59.4 |
| 8 | T0466-D1 | 72 | TBM | 0 | 47 | 155 | 0.74 | 2.1 | 0.25 | 11.7 | 42.9 | 42.9 | 33.3 | 18.1 | 71.4 | 57.1 | 52.8 | 41.7 |
| 8 | T0468-D1 | 61 | TBM | 8 | 24 | 112 | 0.67 | 4.6 | 0.29 | 8.5 | 50.0 | 66.7 | 48.4 | 31.2 | 100.0 | 91.7 | 67.7 | 50.8 |
| 8 | T0469-D1 | 63 | TBM | 33 | 0 | 94 | 0.88 | 1.3 | 0.70 | 2.6 | 83.3 | 84.6 | 53.1 | 38.1 | 100.0 | 84.6 | 75.0 | 54.0 |
| 8 | T0470-D1 | 111 | TBM-HA | 73 | 0 | 157 | 0.86 | 1.9 | 0.43 | 11.0 | 45.5 | 27.3 | 12.5 | 7.2 | 27.3 | 27.3 | 25.0 | 20.7 |
| 8 | T0470-D2 | 77 | TBM-HA | 45 | 0 | 71 | 0.78 | 2.2 | 0.69 | 4.4 | 62.5 | 66.7 | 35.9 | 23.4 | 75.0 | 73.3 | 59.0 | 48.1 |
| 8 | T0471-D1 | 88 | TBM | 30 | 29 | 191 | 0.80 | 2.1 | 0.31 | 13.2 | 55.6 | 33.3 | 25.0 | 14.8 | 55.6 | 61.1 | 59.1 | 42.1 |
| 8 | T0473-D1 | 60 | TBM | 35 | 0 | 84 | 0.87 | 1.1 | 0.77 | 1.8 | 83.3 | 91.7 | 63.3 | 45.0 | 100.0 | 91.7 | 80.0 | 68.3 |
| 8 | T0475-D1 | 118 | TBM | 33 | 57 | 255 | 0.91 | 1.3 | 0.32 | 23.6 | 41.7 | 50.0 | 27.1 | 22.0 | 91.7 | 91.7 | 81.4 | 66.1 |
| 8 | T0476-D1 | 87 | TBM-HA | 30 | 6 | 97 | 0.80 | 2.1 | 0.39 | 8.5 | 33.3 | 29.4 | 22.7 | 13.8 | 22.2 | 35.3 | 27.3 | 23.0 |
| 8 | T0477-D1 | 240 | TBM | 107 | 33 | 515 | 0.94 | 1.5 | 0.31 | 18.3 | 58.3 | 41.7 | 20.8 | 11.7 | 33.3 | 29.2 | 22.5 | 20.4 |
| 8 | T0478-D1 | 126 | TBM | 99 | 0 | 174 | 0.95 | 1.0 | 0.81 | 2.9 | 76.9 | 84.0 | 60.3 | 43.7 | 76.9 | 80.0 | 66.7 | 54.8 |
| 8 | T0478-D2 | 130 | TBM | 106 | 0 | 171 | 0.94 | 1.2 | 0.78 | 2.6 | 76.9 | 61.5 | 60.0 | 43.1 | 92.3 | 73.1 | 64.6 | 52.3 |
| 8 | T0479-D1 | 122 | TBM-HA | 20 | 53 | 311 | 0.91 | 1.4 | 0.34 | 8.9 | 58.3 | 58.3 | 24.6 | 19.7 | 100.0 | 87.5 | 77.1 | 51.6 |
| 8 | T0481-D1 | 135 | TBM | 85 | 4 | 194 | 0.89 | 2.0 | 0.68 | 4.4 | 92.9 | 81.5 | 69.1 | 55.6 | 85.7 | 85.2 | 69.1 | 60.0 |
| 8 | T0482-D1 | 67 | FM | 17 | 32 | 119 | 0.53 | 4.7 | 0.33 | 12.5 | 14.3 | 7.7 | 5.9 | 4.5 | 71.4 | 76.9 | 73.5 | 47.8 |
| 8 | T0483-D1 | 273 | TBM | 83 | 50 | 536 | 0.93 | 1.8 | 0.29 | 19.1 | 40.7 | 25.5 | 10.2 | 5.5 | 77.8 | 72.7 | 46.0 | 30.0 |
| 8 | T0485-D1 | 207 | TBM | 94 | 50 | 438 | 0.94 | 1.5 | 0.39 | 13.8 | 76.2 | 56.1 | 33.0 | 22.9 | 57.1 | 48.8 | 44.7 | 31.2 |
| 8 | T0486-D1 | 214 | TBM-HA | 91 | 42 | 482 | 0.95 | 1.4 | 0.48 | 11.1 | 90.5 | 90.7 | 67.3 | 51.4 | 71.4 | 67.4 | 51.4 | 44.4 |
| 8 | T0487-D1 | 194 | TBM | 44 | 59 | 451 | 0.90 | 1.9 | 0.54 | 13.3 | 100.0 | 92.3 | 70.1 | 48.7 | 100.0 | 97.4 | 83.5 | 68.4 |
| 8 | T0487-D2 | 134 | TBM | 47 | 42 | 240 | 0.83 | 2.2 | 0.32 | 16.3 | 7.7 | 22.2 | 16.4 | 14.3 | 92.3 | 70.4 | 55.2 | 42.1 |
| 8 | T0487-D3 | 71 | TBM | 11 | 34 | 151 | 0.87 | 1.3 | 0.32 | 8.8 | 57.1 | 42.9 | 38.9 | 23.9 | 71.4 | 64.3 | 52.8 | 45.1 |
| 8 | T0487-D4 | 87 | TBM | 13 | 20 | 184 | 0.85 | 1.6 | 0.38 | 6.9 | 11.1 | 11.8 | 13.6 | 11.5 | 55.6 | 58.8 | 56.8 | 41.4 |
| 8 | T0487-D5 | 147 | TBM | 57 | 29 | 283 | 0.94 | 1.3 | 0.32 | 12.9 | 33.3 | 20.7 | 18.9 | 13.6 | 46.7 | 37.9 | 25.7 | 22.5 |
| 8 | T0488-D1 | 86 | TBM-HA | 15 | 32 | 180 | 0.87 | 1.5 | 0.31 | 11.5 | 11.1 | 5.9 | 16.3 | 11.6 | 22.2 | 23.5 | 23.3 | 29.1 |
| 8 | T0489-D1 | 210 | TBM | 104 | 13 | 371 | 0.92 | 1.9 | 0.57 | 7.5 | 57.1 | 57.1 | 41.9 | 32.4 | 66.7 | 57.1 | 55.2 | 45.7 |
| 8 | T0490-D1 | 361 | TBM | 99 | 94 | 952 | 0.95 | 1.6 | 0.60 | 9.5 | 88.9 | 90.3 | 83.4 | 70.6 | 88.9 | 93.1 | 89.5 | 82.3 |
| 8 | T0491-D1 | 96 | TBM-HA | 6 | 59 | 223 | 0.83 | 2.6 | 0.74 | 2.9 | 100.0 | 100.0 | 93.8 | 79.2 | 100.0 | 100.0 | 95.8 | 94.8 |
| 8 | T0492-D1 | 69 | TBM | 13 | 26 | 148 | 0.85 | 1.4 | 0.71 | 2.2 | 100.0 | 100.0 | 82.9 | 75.4 | 100.0 | 100.0 | 97.1 | 84.1 |
| 8 | T0493-D1 | 149 | TBM | 45 | 37 | 335 | 0.87 | 2.3 | 0.42 | 10.1 | 93.3 | 70.0 | 57.3 | 36.9 | 46.7 | 40.0 | 38.7 | 26.9 |
| 8 | T0494-D1 | 345 | TBM | 107 | 58 | 706 | 0.93 | 2.1 | 0.32 | 19.7 | 31.4 | 18.8 | 9.8 | 7.0 | 80.0 | 78.3 | 57.2 | 38.8 |
| 8 | T0495-D1 | 139 | TBM | 44 | 34 | 283 | 0.91 | 1.5 | 0.49 | 8.7 | 85.7 | 64.3 | 44.3 | 27.3 | 85.7 | 85.7 | 84.3 | 66.2 |
| 8 | T0496-D1 | 120 | FM | 42 | 22 | 225 | 0.92 | 1.3 | 0.41 | 9.0 | 75.0 | 62.5 | 50.0 | 35.0 | 91.7 | 83.3 | 61.7 | 54.2 |
| 8 | T0497-D1 | 124 | TBM | 31 | 49 | 264 | 0.87 | 1.7 | 0.58 | 5.4 | 100.0 | 100.0 | 95.2 | 79.0 | 100.0 | 100.0 | 95.2 | 86.3 |
| 8 | T0501-D1 | 213 | TBM | 90 | 29 | 477 | 0.94 | 1.4 | 0.77 | 4.3 | 100.0 | 93.0 | 82.2 | 64.8 | 100.0 | 93.0 | 85.1 | 69.5 |
| 8 | T0501-D2 | 126 | TBM | 44 | 31 | 264 | 0.90 | 1.5 | 0.52 | 6.4 | 84.6 | 84.0 | 73.0 | 47.6 | 100.0 | 96.0 | 92.1 | 78.6 |
| 8 | T0502-D1 | 93 | TBM | 0 | 60 | 227 | 0.85 | 1.7 | 0.49 | 7.8 | 88.9 | 94.7 | 83.0 | 62.4 | 100.0 | 94.7 | 93.6 | 88.2 |
| 8 | T0503-D1 | 144 | TBM | 56 | 43 | 278 | 0.92 | 1.5 | 0.38 | 8.1 | 85.7 | 82.8 | 69.4 | 43.1 | 85.7 | 75.9 | 52.8 | 43.8 |
| 8 | T0504-D1 | 62 | TBM | 0 | 38 | 129 | 0.83 | 1.5 | 0.40 | 6.5 | 0.0 | 16.7 | 19.4 | 16.4 | 66.7 | 75.0 | 77.4 | 70.5 |
| 8 | T0504-D2 | 90 | TBM | 13 | 29 | 167 | 0.85 | 1.6 | 0.35 | 6.8 | 44.4 | 38.9 | 35.6 | 28.9 | 100.0 | 94.4 | 77.8 | 65.6 |
| 8 | T0505-D1 | 159 | TBM-HA | 60 | 25 | 363 | 0.94 | 1.2 | 0.31 | 15.6 | 87.5 | 50.0 | 20.0 | 11.3 | 62.5 | 43.8 | 31.3 | 27.0 |
| 8 | T0505-D2 | 104 | TBM | 30 | 29 | 214 | 0.82 | 3.1 | 0.38 | 11.3 | 30.0 | 33.3 | 30.8 | 28.9 | 100.0 | 85.7 | 53.9 | 41.4 |
| 8 | T0506-D1 | 137 | TBM-HA | 38 | 50 | 286 | 0.89 | 1.9 | 0.68 | 7.8 | 100.0 | 96.3 | 87.0 | 63.5 | 100.0 | 96.3 | 94.2 | 81.8 |
| 8 | T0506-D2 | 78 | TBM | 34 | 16 | 135 | 0.88 | 1.3 | 0.60 | 3.2 | 62.5 | 37.5 | 30.8 | 21.8 | 87.5 | 93.8 | 71.8 | 51.3 |
| 8 | T0507-D1 | 124 | TBM | 51 | 24 | 241 | 0.93 | 1.2 | 0.66 | 3.6 | 91.7 | 80.0 | 64.5 | 54.0 | 91.7 | 84.0 | 67.7 | 62.1 |
| 8 | T0508-D1 | 174 | TBM-HA | 62 | 49 | 409 | 0.94 | 1.3 | 0.32 | 14.1 | 52.9 | 40.0 | 20.7 | 14.9 | 29.4 | 40.0 | 31.0 | 26.4 |
| 8 | T0509-D1 | 209 | TBM | 85 | 41 | 461 | 0.95 | 1.3 | 0.73 | 4.6 | 100.0 | 97.6 | 81.9 | 70.8 | 100.0 | 100.0 | 87.6 | 76.6 |
| 8 | T0510-D1 | 151 | TBM | 21 | 65 | 347 | 0.85 | 2.4 | 0.51 | 8.8 | 60.0 | 53.3 | 39.5 | 28.5 | 100.0 | 100.0 | 97.4 | 84.1 |
| 8 | T0510-D2 | 68 | TBM | 33 | 0 | 81 | 0.86 | 1.5 | 0.67 | 2.9 | 42.9 | 28.6 | 17.7 | 16.2 | 100.0 | 71.4 | 61.8 | 47.1 |
| 8 | T0511-D1 | 249 | TBM | 108 | 34 | 611 | 0.95 | 1.5 | 0.34 | 16.9 | 88.0 | 74.0 | 52.8 | 32.1 | 68.0 | 52.0 | 33.6 | 29.3 |
| 8 | T0512-D1 | 320 | TBM | 6 | 157 | 988 | 0.94 | 1.7 | 0.27 | 20.2 | 43.8 | 28.1 | 13.8 | 9.1 | 93.8 | 87.5 | 83.1 | 70.6 |
| 8 | T0513-D1 | 208 | TBM | 51 | 65 | 472 | 0.88 | 2.7 | 0.60 | 9.3 | 100.0 | 88.1 | 75.0 | 54.3 | 95.2 | 90.5 | 78.9 | 73.6 |
| 8 | T0513-D2 | 69 | FM | 24 | 20 | 93 | 0.83 | 1.6 | 0.45 | 5.9 | 14.3 | 21.4 | 17.1 | 17.4 | 100.0 | 100.0 | 80.0 | 55.1 |
| 8 | T0514-D1 | 144 | TBM | 21 | 70 | 327 | 0.90 | 1.7 | 0.57 | 5.6 | 85.7 | 79.3 | 62.5 | 50.0 | 78.6 | 79.3 | 81.9 | 75.7 |
| 9 | T0515-D1 | 348 | TBM | 111 | 81 | 852 | 0.94 | 1.9 | 0.58 | 9.5 | 88.6 | 85.7 | 73.6 | 54.6 | 94.3 | 87.1 | 78.7 | 65.8 |
| 9 | T0516-D1 | 227 | TBM | 167 | 0 | 345 | 0.95 | 1.5 | 0.76 | 4.4 | 73.9 | 66.7 | 57.0 | 44.5 | 78.3 | 68.9 | 65.8 | 50.7 |
| 9 | T0517-D1 | 159 | TBM | 65 | 24 | 287 | 0.92 | 1.5 | 0.51 | 10.8 | 93.8 | 96.9 | 78.8 | 59.1 | 93.8 | 93.8 | 80.0 | 65.4 |
| 9 | T0518-D1 | 256 | TBM | 16 | 97 | 681 | 0.92 | 1.9 | 0.52 | 13.9 | 100.0 | 98.0 | 89.1 | 69.9 | 100.0 | 98.0 | 90.6 | 78.5 |
| 9 | T0520-D1 | 173 | TBM | 57 | 60 | 371 | 0.90 | 1.9 | 0.44 | 13.1 | 47.1 | 51.4 | 43.7 | 38.7 | 82.4 | 71.4 | 48.3 | 37.6 |
| 9 | T0521-D1 | 98 | TBM | 64 | 0 | 98 | 0.78 | 2.9 | 0.30 | 14.0 | 10.0 | 10.0 | 8.2 | 4.1 | 20.0 | 30.0 | 16.3 | 14.3 |
| 9 | T0521-D2 | 70 | TBM | 41 | 4 | 68 | 0.76 | 2.3 | 0.34 | 17.0 | 0.0 | 0.0 | 5.7 | 8.6 | 42.9 | 42.9 | 20.0 | 11.4 |
| 9 | T0522-D1 | 131 | TBM | 42 | 29 | 261 | 0.93 | 1.3 | 0.66 | 5.2 | 84.6 | 76.9 | 53.0 | 35.9 | 92.3 | 92.3 | 84.9 | 71.8 |
| 9 | T0523-D1 | 111 | TBM | 35 | 38 | 218 | 0.88 | 1.6 | 0.31 | 9.7 | 9.1 | 4.6 | 1.8 | 0.9 | 81.8 | 72.7 | 60.7 | 44.1 |
| 9 | T0524-D1 | 321 | TBM | 26 | 154 | 923 | 0.94 | 1.8 | 0.67 | 7.5 | 96.9 | 98.4 | 85.7 | 66.7 | 96.9 | 96.9 | 94.4 | 85.1 |
| 9 | T0525-D1 | 205 | TBM | 77 | 35 | 420 | 0.91 | 1.8 | 0.43 | 10.8 | 66.7 | 65.9 | 38.8 | 28.8 | 95.2 | 87.8 | 67.0 | 46.3 |
| 9 | T0526-D1 | 290 | TBM | 0 | 141 | 844 | 0.93 | 2.0 | 0.64 | 6.4 | 100.0 | 93.1 | 84.8 | 69.7 | 100.0 | 100.0 | 92.4 | 79.7 |
| 9 | T0527-D1 | 99 | TBM | 20 | 39 | 185 | 0.79 | 2.4 | 0.35 | 10.9 | 60.0 | 65.0 | 38.0 | 25.3 | 100.0 | 95.0 | 80.0 | 63.6 |
| 9 | T0528-D1 | 204 | TBM | 87 | 39 | 514 | 0.95 | 1.3 | 0.82 | 3.1 | 95.0 | 95.1 | 92.2 | 74.5 | 95.0 | 95.1 | 94.1 | 86.8 |
| 9 | T0528-D2 | 160 | TBM | 44 | 45 | 374 | 0.92 | 1.6 | 0.60 | 9.4 | 93.8 | 96.9 | 90.0 | 69.4 | 93.8 | 96.9 | 90.0 | 70.0 |
| 9 | T0529-D1 | 322 | FM | 170 | 14 | 629 | 0.95 | 1.9 | 0.22 | 29.8 | 12.5 | 10.9 | 9.9 | 7.5 | 34.4 | 26.6 | 20.5 | 14.6 |
| 9 | T0529-D2 | 193 | TBM | 66 | 33 | 413 | 0.93 | 1.5 | 0.26 | 19.4 | 5.3 | 5.1 | 5.2 | 4.2 | 89.5 | 66.7 | 53.6 | 33.7 |
| 9 | T0530-D1 | 80 | TBM | 7 | 42 | 174 | 0.85 | 1.8 | 0.43 | 6.2 | 100.0 | 93.8 | 77.5 | 53.8 | 100.0 | 100.0 | 95.0 | 91.3 |
| 9 | T0532-D1 | 470 | TBM | 222 | 13 | 965 | 0.96 | 1.6 | 0.29 | 29.7 | 70.2 | 46.8 | 23.4 | 13.0 | 53.2 | 42.6 | 28.5 | 23.6 |
| 9 | T0533-D1 | 199 | TBM | 45 | 63 | 474 | 0.91 | 1.8 | 0.45 | 10.5 | 100.0 | 95.0 | 81.0 | 66.8 | 95.0 | 85.0 | 79.0 | 71.9 |
| 9 | T0533-D2 | 91 | TBM | 24 | 20 | 197 | 0.90 | 1.3 | 0.70 | 3.5 | 100.0 | 94.4 | 82.6 | 73.6 | 88.9 | 88.9 | 84.8 | 74.7 |
| 9 | T0534-D1 | 178 | FM | 107 | 4 | 266 | 0.90 | 1.8 | 0.43 | 24.2 | 83.3 | 63.9 | 43.8 | 28.7 | 94.4 | 80.6 | 53.9 | 36.0 |
| 9 | T0534-D2 | 176 | FM | 118 | 0 | 299 | 0.94 | 1.3 | 0.60 | 9.5 | 100.0 | 91.4 | 65.9 | 44.3 | 100.0 | 97.1 | 80.7 | 63.1 |
| 9 | T0536-D1 | 93 | TBM | 23 | 36 | 173 | 0.78 | 3.2 | 0.27 | 11.3 | 66.7 | 31.6 | 14.9 | 11.8 | 100.0 | 94.7 | 80.9 | 55.9 |
| 9 | T0537-D1 | 286 | FM | 8 | 133 | 1068 | 0.94 | 1.7 | 0.54 | 8.0 | 86.2 | 86.0 | 83.2 | 72.0 | 86.2 | 86.0 | 80.4 | 69.9 |
| 9 | T0539-D1 | 68 | TBM | 8 | 16 | 109 | 0.78 | 2.0 | 0.28 | 10.7 | 14.3 | 7.1 | 2.9 | 2.9 | 14.3 | 14.3 | 11.8 | 10.3 |
| 9 | T0540-D1 | 90 | TBM | 0 | 59 | 206 | 0.90 | 1.4 | 0.41 | 5.9 | 0.0 | 5.6 | 13.3 | 12.2 | 88.9 | 88.9 | 86.7 | 73.3 |
| 9 | T0541-D1 | 102 | TBM | 0 | 56 | 279 | 0.86 | 1.8 | 0.61 | 6.0 | 100.0 | 100.0 | 96.1 | 81.4 | 100.0 | 100.0 | 94.1 | 80.4 |
| 9 | T0542-D1 | 301 | TBM | 60 | 92 | 842 | 0.95 | 1.5 | 0.61 | 13.5 | 100.0 | 95.0 | 91.4 | 77.1 | 100.0 | 93.3 | 94.7 | 84.4 |
| 9 | T0542-D2 | 265 | TBM | 138 | 15 | 533 | 0.83 | 3.1 | 0.76 | 10.2 | 92.6 | 94.3 | 82.0 | 65.3 | 96.3 | 92.5 | 87.2 | 71.7 |
| 9 | T0543-D3 | 386 | TBM | 84 | 70 | 933 | 0.94 | 2.0 | 0.64 | 10.2 | 89.7 | 84.4 | 70.0 | 56.2 | 89.7 | 87.0 | 75.1 | 61.9 |
| 9 | T0543-D4 | 312 | TBM | 63 | 59 | 725 | 0.93 | 1.9 | 0.41 | 22.6 | 96.8 | 85.5 | 68.6 | 46.8 | 96.8 | 88.7 | 80.1 | 62.8 |
| 9 | T0544-D1 | 135 | FM | 75 | 0 | 176 | 0.88 | 1.7 | 0.66 | 5.8 | 85.7 | 77.8 | 50.0 | 31.9 | 85.7 | 77.8 | 63.2 | 47.4 |
| 9 | T0545-D1 | 131 | TBM | 44 | 25 | 259 | 0.83 | 2.2 | 0.58 | 6.4 | 92.3 | 84.6 | 69.7 | 47.3 | 92.3 | 88.5 | 84.9 | 64.1 |
| 9 | T0547-D1 | 183 | TBM | 15 | 47 | 436 | 0.92 | 1.6 | 0.33 | 14.7 | 77.8 | 62.2 | 43.5 | 27.3 | 83.3 | 83.8 | 66.3 | 48.1 |
| 9 | T0547-D2 | 265 | TBM | 117 | 39 | 602 | 0.96 | 1.3 | 0.75 | 4.7 | 96.3 | 90.6 | 79.7 | 64.9 | 96.3 | 92.5 | 82.7 | 71.3 |
| 9 | T0547-D3 | 79 | FM | 52 | 0 | 106 | 0.88 | 1.6 | 0.68 | 3.4 | 75.0 | 81.3 | 57.5 | 45.6 | 87.5 | 81.3 | 75.0 | 50.6 |
| 9 | T0548-D2 | 60 | TBM | 43 | 0 | 45 | 0.78 | 2.1 | 0.61 | 5.5 | 100.0 | 75.0 | 43.3 | 30.0 | 66.7 | 83.3 | 56.7 | 38.3 |
| 9 | T0550-D1 | 143 | TBM-HA | 12 | 61 | 305 | 0.90 | 1.7 | 0.59 | 7.4 | 100.0 | 96.6 | 81.9 | 60.8 | 100.0 | 93.1 | 75.0 | 61.5 |
| 9 | T0550-D2 | 162 | FM | 4 | 84 | 374 | 0.83 | 3.5 | 0.38 | 13.4 | 50.0 | 50.0 | 49.4 | 39.5 | 87.5 | 78.1 | 65.4 | 58.0 |
| 9 | T0551-D1 | 63 | TBM | 10 | 33 | 111 | 0.77 | 1.6 | 0.43 | 6.7 | 16.7 | 7.7 | 12.5 | 7.9 | 83.3 | 76.9 | 71.9 | 73.0 |
| 9 | T0552-D1 | 73 | TBM | 0 | 43 | 158 | 0.74 | 2.8 | 0.27 | 8.8 | 42.9 | 33.3 | 29.7 | 23.3 | 42.9 | 33.3 | 18.9 | 21.9 |
| 9 | T0553-D1 | 63 | FM | 41 | 0 | 59 | 0.84 | 1.5 | 0.54 | 8.0 | 100.0 | 53.9 | 40.6 | 25.4 | 100.0 | 76.9 | 50.0 | 38.1 |
| 9 | T0553-D2 | 71 | FM | 46 | 0 | 59 | 0.79 | 2.3 | 0.54 | 4.3 | 57.1 | 57.1 | 44.4 | 23.9 | 71.4 | 57.1 | 52.8 | 42.3 |
| 9 | T0555-D1 | 134 | FM | 80 | 0 | 154 | 0.86 | 2.0 | 0.64 | 5.2 | 92.3 | 81.5 | 56.7 | 35.1 | 100.0 | 85.2 | 65.7 | 48.5 |
| 9 | T0557-D1 | 120 | TBM | 36 | 34 | 249 | 0.88 | 1.9 | 0.55 | 6.6 | 91.7 | 91.7 | 75.0 | 57.5 | 91.7 | 95.8 | 90.0 | 72.5 |
| 9 | T0558-D1 | 272 | TBM | 0 | 127 | 785 | 0.94 | 1.8 | 0.32 | 15.3 | 63.0 | 44.4 | 28.7 | 18.0 | 96.3 | 92.6 | 89.7 | 81.3 |
| 9 | T0559-D1 | 67 | TBM | 37 | 14 | 105 | 0.90 | 1.0 | 0.61 | 6.1 | 42.9 | 46.2 | 35.3 | 29.9 | 85.7 | 92.3 | 73.5 | 52.2 |
| 9 | T0560-D1 | 64 | TBM | 36 | 12 | 98 | 0.86 | 1.3 | 0.68 | 3.0 | 33.3 | 38.5 | 40.6 | 31.3 | 83.3 | 76.9 | 62.5 | 56.3 |
| 9 | T0561-D1 | 151 | FM | 103 | 0 | 161 | 0.84 | 3.2 | 0.39 | 17.6 | 13.3 | 10.0 | 10.5 | 11.3 | 13.3 | 13.3 | 10.5 | 10.6 |
| 9 | T0562-D1 | 123 | TBM | 28 | 26 | 258 | 0.87 | 1.7 | 0.42 | 10.4 | 66.7 | 52.0 | 45.2 | 29.3 | 75.0 | 80.0 | 79.0 | 60.2 |
| 9 | T0563-D1 | 228 | TBM | 66 | 51 | 488 | 0.93 | 1.6 | 0.68 | 5.4 | 95.7 | 95.7 | 85.1 | 67.1 | 87.0 | 93.5 | 82.5 | 67.5 |
| 9 | T0564-D1 | 61 | TBM | 4 | 33 | 120 | 0.72 | 2.0 | 0.31 | 8.5 | 100.0 | 66.7 | 61.3 | 45.9 | 100.0 | 75.0 | 77.4 | 62.3 |
| 9 | T0565-D1 | 304 | TBM | 41 | 115 | 782 | 0.93 | 1.9 | 0.26 | 27.6 | 43.3 | 34.4 | 24.3 | 19.4 | 93.3 | 93.4 | 74.3 | 50.3 |
| 9 | T0566-D1 | 143 | TBM | 52 | 59 | 269 | 0.84 | 2.1 | 0.54 | 6.2 | 71.4 | 62.1 | 51.4 | 43.4 | 100.0 | 93.1 | 77.8 | 71.3 |
| 9 | T0567-D1 | 135 | TBM | 57 | 20 | 251 | 0.90 | 1.6 | 0.27 | 14.2 | 14.3 | 7.4 | 5.9 | 7.4 | 7.1 | 3.7 | 10.3 | 7.4 |
| 9 | T0568-D1 | 120 | TBM | 0 | 65 | 285 | 0.86 | 1.9 | 0.37 | 13.2 | 58.3 | 41.7 | 43.3 | 34.2 | 83.3 | 75.0 | 53.3 | 39.2 |
| 9 | T0569-D1 | 78 | TBM | 0 | 46 | 187 | 0.83 | 1.7 | 0.46 | 6.4 | 87.5 | 81.3 | 76.9 | 60.3 | 87.5 | 81.3 | 84.6 | 66.7 |
| 9 | T0570-D1 | 233 | TBM | 89 | 47 | 555 | 0.95 | 1.3 | 0.77 | 3.6 | 100.0 | 97.9 | 80.3 | 66.5 | 100.0 | 95.7 | 88.0 | 79.0 |
| 9 | T0571-D1 | 165 | TBM-HA | 9 | 68 | 385 | 0.89 | 2.0 | 0.37 | 26.8 | 100.0 | 100.0 | 67.5 | 52.1 | 100.0 | 100.0 | 72.3 | 61.2 |
| 9 | T0571-D2 | 135 | FM | 0 | 90 | 363 | 0.86 | 2.0 | 0.40 | 10.4 | 78.6 | 59.3 | 32.4 | 26.7 | 85.7 | 92.6 | 80.9 | 72.6 |
| 9 | T0572-D1 | 86 | TBM | 6 | 47 | 174 | 0.86 | 1.6 | 0.73 | 2.4 | 100.0 | 88.2 | 90.7 | 83.7 | 100.0 | 88.2 | 93.0 | 89.5 |
| 9 | T0573-D1 | 254 | TBM | 75 | 40 | 574 | 0.87 | 2.5 | 0.65 | 6.0 | 92.0 | 94.1 | 79.5 | 64.6 | 92.0 | 94.1 | 86.6 | 70.1 |
| 9 | T0574-D1 | 102 | TBM | 0 | 61 | 245 | 0.86 | 1.9 | 0.33 | 8.6 | 80.0 | 65.0 | 49.0 | 40.2 | 60.0 | 60.0 | 54.9 | 44.1 |
| 9 | T0575-D1 | 63 | TBM | 36 | 0 | 61 | 0.87 | 1.3 | 0.39 | 14.4 | 0.0 | 0.0 | 6.3 | 9.5 | 33.3 | 30.8 | 25.0 | 19.1 |
| 9 | T0575-D2 | 127 | TBM | 100 | 0 | 128 | 0.78 | 3.2 | 0.67 | 12.5 | 53.9 | 60.0 | 42.2 | 25.2 | 76.9 | 68.0 | 56.3 | 39.4 |
| 9 | T0576-D1 | 133 | TBM | 24 | 57 | 257 | 0.89 | 1.7 | 0.24 | 14.7 | 7.7 | 3.7 | 1.5 | 3.0 | 76.9 | 59.3 | 44.8 | 33.8 |
| 9 | T0578-D1 | 148 | FM | 48 | 36 | 256 | 0.88 | 2.0 | 0.39 | 14.4 | 20.0 | 10.0 | 12.2 | 12.2 | 73.3 | 63.3 | 37.8 | 26.4 |
| 9 | T0579-D1 | 60 | TBM | 0 | 33 | 149 | 0.86 | 1.2 | 0.38 | 7.8 | 16.7 | 25.0 | 13.3 | 15.0 | 100.0 | 91.7 | 73.3 | 63.3 |
| 9 | T0579-D2 | 64 | TBM | 0 | 25 | 141 | 0.76 | 1.7 | 0.48 | 4.2 | 50.0 | 69.2 | 62.5 | 46.9 | 66.7 | 76.9 | 84.4 | 75.0 |
| 9 | T0580-D1 | 104 | TBM | 42 | 22 | 215 | 0.92 | 1.2 | 0.63 | 3.5 | 100.0 | 100.0 | 80.8 | 64.4 | 100.0 | 100.0 | 84.6 | 73.1 |
| 9 | T0581-D1 | 105 | FM | 45 | 34 | 190 | 0.90 | 1.5 | 0.40 | 8.5 | 18.2 | 33.3 | 28.3 | 21.0 | 63.6 | 76.2 | 52.8 | 46.7 |
| 9 | T0582-D1 | 121 | TBM | 0 | 57 | 283 | 0.82 | 2.3 | 0.34 | 12.8 | 91.7 | 79.2 | 60.7 | 38.8 | 83.3 | 75.0 | 72.1 | 61.2 |
| 9 | T0582-D2 | 99 | TBM | 0 | 54 | 254 | 0.85 | 2.1 | 0.56 | 8.3 | 100.0 | 95.0 | 86.0 | 78.8 | 100.0 | 95.0 | 92.0 | 87.9 |
| 9 | T0584-D1 | 338 | TBM | 245 | 4 | 637 | 0.96 | 1.5 | 0.63 | 8.6 | 91.2 | 89.7 | 75.7 | 53.6 | 88.2 | 88.2 | 82.8 | 64.5 |
| 9 | T0585-D1 | 212 | TBM | 87 | 38 | 494 | 0.91 | 2.4 | 0.76 | 9.7 | 100.0 | 100.0 | 88.7 | 84.4 | 100.0 | 100.0 | 88.7 | 84.9 |
| 9 | T0586-D1 | 80 | TBM | 35 | 8 | 109 | 0.85 | 1.5 | 0.39 | 6.7 | 0.0 | 12.5 | 10.0 | 7.5 | 50.0 | 56.3 | 37.5 | 32.5 |
| 9 | T0588-D1 | 381 | TBM | 191 | 11 | 760 | 0.95 | 1.7 | 0.52 | 17.8 | 73.7 | 68.4 | 48.7 | 34.4 | 92.1 | 77.6 | 56.5 | 43.6 |
| 9 | T0589-D1 | 228 | TBM | 76 | 56 | 495 | 0.92 | 1.9 | 0.72 | 6.0 | 100.0 | 97.8 | 79.0 | 60.5 | 100.0 | 95.7 | 87.7 | 76.3 |
| 9 | T0589-D2 | 82 | TBM | 58 | 0 | 74 | 0.81 | 1.9 | 0.50 | 7.2 | 75.0 | 50.0 | 29.3 | 15.9 | 87.5 | 75.0 | 61.0 | 43.9 |
| 9 | T0589-D3 | 94 | TBM | 36 | 18 | 161 | 0.88 | 1.4 | 0.79 | 3.2 | 88.9 | 88.2 | 80.5 | 69.1 | 88.9 | 88.9 | 90.7 | 83.7 |
| 9 | T0590-D1 | 72 | TBM | 0 | 26 | 162 | 0.74 | 2.7 | 0.41 | 7.8 | 100.0 | 92.9 | 75.0 | 58.3 | 100.0 | 92.9 | 91.7 | 77.8 |
| 9 | T0591-D1 | 380 | TBM | 141 | 48 | 966 | 0.96 | 1.4 | 0.34 | 18.2 | 92.1 | 80.3 | 56.3 | 34.7 | 84.2 | 75.0 | 60.5 | 48.7 |
| 9 | T0592-D1 | 137 | TBM | 45 | 21 | 232 | 0.86 | 2.4 | 0.45 | 8.6 | 85.7 | 70.4 | 56.5 | 44.5 | 64.3 | 48.2 | 37.7 | 33.6 |
| 9 | T0593-D1 | 195 | TBM | 70 | 35 | 412 | 0.94 | 1.4 | 0.73 | 3.9 | 100.0 | 84.6 | 75.5 | 58.5 | 95.0 | 87.2 | 78.6 | 68.2 |
| 9 | T0594-D1 | 140 | TBM | 34 | 50 | 304 | 0.88 | 1.7 | 0.71 | 4.5 | 100.0 | 89.3 | 62.9 | 44.3 | 100.0 | 100.0 | 95.7 | 82.9 |
| 9 | T0596-D2 | 121 | TBM | 90 | 0 | 116 | 0.89 | 1.7 | 0.70 | 3.4 | 66.7 | 66.7 | 50.8 | 32.2 | 75.0 | 66.7 | 62.3 | 44.6 |
| 9 | T0597-D1 | 359 | TBM | 120 | 47 | 709 | 0.89 | 2.8 | 0.34 | 21.5 | 63.9 | 50.0 | 28.9 | 14.8 | 88.9 | 86.1 | 60.6 | 39.3 |
| 9 | T0598-D1 | 127 | TBM | 64 | 11 | 141 | 0.71 | 4.0 | 0.36 | 13.4 | 61.5 | 44.0 | 25.0 | 19.7 | 53.9 | 56.0 | 29.7 | 21.3 |
| 9 | T0599-D1 | 367 | TBM | 113 | 127 | 962 | 0.93 | 2.3 | 0.62 | 11.9 | 97.3 | 90.4 | 75.5 | 60.8 | 97.3 | 95.9 | 87.5 | 72.2 |
| 9 | T0601-D1 | 442 | TBM | 108 | 160 | 1248 | 0.95 | 1.8 | 0.56 | 14.6 | 97.7 | 96.6 | 86.9 | 71.5 | 100.0 | 98.9 | 94.6 | 81.2 |
| 9 | T0603-D1 | 260 | TBM | 129 | 35 | 497 | 0.93 | 1.7 | 0.43 | 13.3 | 69.2 | 65.4 | 50.8 | 36.2 | 84.6 | 71.2 | 66.9 | 50.0 |
| 9 | T0604-D1 | 80 | FM | 16 | 33 | 144 | 0.86 | 1.5 | 0.63 | 3.4 | 100.0 | 100.0 | 75.0 | 52.5 | 100.0 | 100.0 | 92.5 | 77.5 |
| 9 | T0604-D2 | 249 | TBM | 90 | 30 | 579 | 0.94 | 1.6 | 0.28 | 17.9 | 52.0 | 36.0 | 19.2 | 11.2 | 88.0 | 78.0 | 48.8 | 32.9 |
| 9 | T0604-D3 | 205 | FM | 45 | 56 | 558 | 0.94 | 1.3 | 0.65 | 5.0 | 90.5 | 90.2 | 74.8 | 60.5 | 81.0 | 85.4 | 71.8 | 61.5 |
| 9 | T0606-D1 | 120 | TBM | 42 | 31 | 240 | 0.93 | 1.2 | 0.29 | 14.3 | 41.7 | 33.3 | 26.7 | 20.0 | 50.0 | 41.7 | 31.7 | 22.5 |
| 9 | T0607-D1 | 469 | TBM | 165 | 95 | 1145 | 0.91 | 3.1 | 0.55 | 12.8 | 97.9 | 88.3 | 78.7 | 63.5 | 97.9 | 91.5 | 79.6 | 67.4 |
| 9 | T0608-D1 | 89 | FM | 52 | 0 | 100 | 0.91 | 1.2 | 0.37 | 12.8 | 0.0 | 0.0 | 4.4 | 10.1 | 33.3 | 27.8 | 13.3 | 14.6 |
| 9 | T0608-D2 | 161 | TBM | 11 | 60 | 443 | 0.89 | 1.9 | 0.42 | 9.2 | 75.0 | 65.6 | 50.6 | 34.2 | 93.8 | 96.9 | 74.1 | 55.9 |
| 9 | T0609-D1 | 335 | TBM | 10 | 162 | 946 | 0.94 | 1.8 | 0.65 | 8.3 | 100.0 | 94.0 | 80.4 | 63.6 | 100.0 | 97.0 | 89.9 | 79.4 |
| 9 | T0610-D1 | 176 | TBM | 40 | 43 | 368 | 0.91 | 1.7 | 0.68 | 4.9 | 83.3 | 77.1 | 69.3 | 49.4 | 88.9 | 94.3 | 84.1 | 76.7 |
| 9 | T0611-D2 | 149 | TBM | 111 | 0 | 165 | 0.90 | 1.7 | 0.57 | 6.2 | 13.3 | 30.0 | 24.0 | 19.5 | 46.7 | 36.7 | 30.7 | 22.8 |
| 9 | T0612-D1 | 106 | TBM | 0 | 58 | 245 | 0.85 | 1.8 | 0.33 | 9.1 | 63.6 | 66.7 | 43.4 | 33.0 | 72.7 | 71.4 | 71.7 | 53.8 |
| 9 | T0613-D1 | 275 | TBM | 89 | 88 | 589 | 0.88 | 2.7 | 0.48 | 11.6 | 71.4 | 70.9 | 66.7 | 49.1 | 75.0 | 74.6 | 71.0 | 62.2 |
| 9 | T0614-D1 | 71 | TBM | 17 | 26 | 135 | 0.88 | 1.2 | 0.36 | 10.2 | 0.0 | 0.0 | 13.9 | 21.1 | 71.4 | 57.1 | 38.9 | 28.2 |
| 9 | T0615-D1 | 174 | TBM | 116 | 0 | 265 | 0.92 | 1.6 | 0.76 | 3.6 | 100.0 | 91.4 | 69.0 | 48.9 | 100.0 | 97.1 | 81.6 | 64.9 |
| 9 | T0616-D1 | 97 | TBM-HA | 41 | 0 | 84 | 0.63 | 6.4 | 0.52 | 12.0 | 70.0 | 47.4 | 26.5 | 22.7 | 90.0 | 79.0 | 61.2 | 40.2 |
| 9 | T0617-D1 | 136 | TBM | 96 | 8 | 143 | 0.71 | 3.6 | 0.39 | 13.8 | 42.9 | 29.6 | 11.8 | 9.6 | 64.3 | 66.7 | 51.5 | 35.3 |
| 9 | T0618-D1 | 158 | FM | 107 | 6 | 221 | 0.91 | 2.0 | 0.59 | 6.2 | 43.8 | 37.5 | 32.9 | 26.0 | 56.3 | 50.0 | 39.2 | 32.9 |
| 9 | T0619-D1 | 101 | TBM | 64 | 0 | 116 | 0.89 | 1.4 | 0.37 | 9.4 | 0.0 | 0.0 | 3.9 | 5.9 | 30.0 | 15.0 | 15.7 | 12.9 |
| 9 | T0620-D1 | 281 | TBM | 54 | 90 | 756 | 0.94 | 1.6 | 0.61 | 10.2 | 96.4 | 94.6 | 85.8 | 67.3 | 100.0 | 98.2 | 90.8 | 79.4 |
| 9 | T0621-D1 | 169 | FM | 18 | 74 | 394 | 0.88 | 2.1 | 0.27 | 14.0 | 29.4 | 20.6 | 14.1 | 16.0 | 23.5 | 38.2 | 27.1 | 22.5 |
| 9 | T0622-D1 | 122 | TBM | 42 | 29 | 247 | 0.91 | 1.4 | 0.61 | 10.1 | 75.0 | 87.5 | 77.1 | 64.8 | 75.0 | 87.5 | 88.5 | 71.3 |
| 9 | T0623-D1 | 187 | TBM | 96 | 21 | 322 | 0.93 | 1.5 | 0.55 | 9.8 | 73.7 | 54.1 | 35.1 | 25.7 | 84.2 | 59.5 | 48.9 | 39.6 |
| 9 | T0624-D1 | 69 | FM | 0 | 38 | 134 | 0.78 | 2.0 | 0.40 | 8.6 | 42.9 | 57.1 | 34.3 | 20.3 | 100.0 | 92.9 | 80.0 | 68.1 |
| 9 | T0625-D1 | 221 | TBM | 24 | 59 | 610 | 0.94 | 1.5 | 0.59 | 12.2 | 95.5 | 81.8 | 74.8 | 70.1 | 95.5 | 88.6 | 78.4 | 72.0 |
| 9 | T0626-D1 | 282 | TBM | 91 | 82 | 613 | 0.92 | 2.1 | 0.50 | 9.8 | 75.0 | 75.0 | 60.3 | 47.5 | 78.6 | 83.9 | 70.2 | 61.4 |
| 9 | T0627-D1 | 244 | TBM | 175 | 0 | 388 | 0.94 | 1.6 | 0.65 | 8.7 | 87.5 | 69.4 | 55.7 | 42.2 | 95.8 | 81.6 | 62.3 | 46.7 |
| 9 | T0628-D1 | 144 | TBM | 39 | 46 | 318 | 0.90 | 1.7 | 0.54 | 7.9 | 85.7 | 75.9 | 44.4 | 36.1 | 92.9 | 89.7 | 77.8 | 58.3 |
| 9 | T0628-D2 | 146 | TBM | 64 | 27 | 291 | 0.92 | 1.5 | 0.64 | 4.7 | 73.3 | 62.1 | 52.1 | 39.7 | 93.3 | 82.8 | 72.6 | 58.2 |
| 9 | T0629-D2 | 159 | FM | 0 | 4 | 31 | 0.16 | 21.4 | 0.12 | 53.1 | 6.3 | 3.1 | 1.3 | 1.3 | 6.3 | 3.1 | 1.3 | 2.5 |
| 9 | T0630-D1 | 99 | TBM | 28 | 31 | 197 | 0.86 | 1.7 | 0.44 | 10.1 | 80.0 | 60.0 | 54.0 | 32.3 | 100.0 | 90.0 | 78.0 | 56.6 |
| 9 | T0632-D1 | 114 | TBM | 24 | 45 | 251 | 0.87 | 1.8 | 0.34 | 10.8 | 45.5 | 56.5 | 29.8 | 17.5 | 90.9 | 82.6 | 61.4 | 46.5 |
| 9 | T0634-D1 | 107 | TBM | 45 | 22 | 213 | 0.89 | 1.5 | 0.32 | 9.8 | 27.3 | 14.3 | 5.6 | 2.8 | 27.3 | 38.1 | 16.7 | 13.1 |
| 9 | T0635-D1 | 161 | TBM | 64 | 27 | 384 | 0.95 | 1.2 | 0.36 | 10.5 | 87.5 | 71.9 | 30.9 | 18.0 | 62.5 | 53.1 | 34.6 | 23.0 |
| 9 | T0636-D1 | 318 | TBM | 123 | 51 | 658 | 0.95 | 1.6 | 0.36 | 14.8 | 84.4 | 75.0 | 55.4 | 33.0 | 90.6 | 75.0 | 59.1 | 46.9 |
| 9 | T0637-D1 | 135 | FM | 109 | 0 | 75 | 0.53 | 6.7 | 0.44 | 14.0 | 57.1 | 55.6 | 41.2 | 25.9 | 57.1 | 59.3 | 47.1 | 31.9 |
| 9 | T0638-D1 | 212 | TBM | 74 | 43 | 419 | 0.89 | 2.1 | 0.71 | 4.4 | 85.7 | 88.1 | 73.6 | 54.7 | 95.2 | 88.1 | 84.0 | 75.5 |
| 9 | T0639-D1 | 124 | FM | 76 | 4 | 133 | 0.85 | 2.3 | 0.43 | 16.5 | 33.3 | 32.0 | 12.9 | 8.9 | 83.3 | 64.0 | 43.6 | 27.4 |
| 9 | T0640-D1 | 207 | TBM | 102 | 35 | 439 | 0.90 | 2.4 | 0.51 | 11.5 | 71.4 | 65.9 | 51.0 | 35.8 | 71.4 | 53.7 | 37.5 | 26.1 |
| 9 | T0641-D1 | 295 | TBM | 125 | 64 | 706 | 0.96 | 1.5 | 0.80 | 3.6 | 90.0 | 89.8 | 85.1 | 73.9 | 93.3 | 86.4 | 83.8 | 74.9 |
| 9 | T0643-D1 | 73 | TBM | 41 | 0 | 84 | 0.83 | 1.7 | 0.52 | 6.9 | 57.1 | 46.7 | 32.4 | 23.3 | 57.1 | 33.3 | 27.0 | 20.6 |
| 10 | T0644-D1 | 141 | TBM | 19 | 60 | 294 | 0.86 | 2.2 | 0.40 | 11.2 | 14.3 | 28.6 | 22.5 | 17.7 | 100.0 | 100.0 | 98.6 | 84.4 |
| 10 | T0645-D1 | 498 | TBM | 220 | 11 | 1169 | 0.97 | 1.6 | 0.29 | 20.9 | 74.0 | 48.0 | 24.9 | 14.5 | 58.0 | 49.0 | 30.5 | 23.9 |
| 10 | T0648-D1 | 86 | TBM | 0 | 35 | 197 | 0.84 | 1.9 | 0.48 | 6.1 | 77.8 | 82.4 | 65.1 | 50.0 | 100.0 | 88.2 | 81.4 | 72.1 |
| 10 | T0649-D1 | 184 | TBM-HA | 36 | 47 | 414 | 0.92 | 1.6 | 0.47 | 15.1 | 44.4 | 54.1 | 59.8 | 45.1 | 44.4 | 54.1 | 69.6 | 54.4 |
| 10 | T0650-D1 | 339 | TBM | 24 | 45 | 969 | 0.76 | 4.4 | 0.34 | 16.1 | 23.5 | 22.1 | 12.9 | 7.4 | 8.8 | 38.2 | 45.3 | 35.7 |
| 10 | T0651-D1 | 95 | TBM | 26 | 34 | 207 | 0.91 | 1.1 | 0.65 | 3.6 | 60.0 | 52.6 | 62.5 | 51.6 | 60.0 | 57.9 | 60.4 | 59.0 |
| 10 | T0651-D2 | 111 | TBM | 42 | 18 | 211 | 0.90 | 1.4 | 0.32 | 13.0 | 27.3 | 13.6 | 5.4 | 4.5 | 45.5 | 31.8 | 25.0 | 16.2 |
| 10 | T0652-D1 | 138 | TBM | 43 | 24 | 220 | 0.84 | 2.2 | 0.29 | 12.9 | 35.7 | 17.9 | 11.6 | 8.0 | 57.1 | 39.3 | 33.3 | 24.6 |
| 10 | T0652-D2 | 83 | TBM | 17 | 26 | 170 | 0.87 | 1.4 | 0.73 | 2.4 | 100.0 | 94.1 | 85.7 | 51.8 | 100.0 | 100.0 | 92.9 | 85.5 |
| 10 | T0653-D1 | 383 | TBM-HA | 0 | 100 | 1188 | 0.82 | 3.7 | 0.35 | 13.6 | 34.2 | 28.6 | 25.5 | 14.9 | 65.8 | 44.2 | 41.7 | 37.9 |
| 10 | T0654-D1 | 134 | TBM | 38 | 25 | 261 | 0.91 | 1.4 | 0.34 | 12.8 | 46.2 | 22.2 | 11.9 | 17.9 | 69.2 | 59.3 | 29.9 | 23.1 |
| 10 | T0655-D1 | 150 | TBM | 52 | 60 | 335 | 0.85 | 2.8 | 0.47 | 11.5 | 80.0 | 70.0 | 52.1 | 30.8 | 100.0 | 93.3 | 81.3 | 63.8 |
| 10 | T0657-D1 | 133 | TBM | 15 | 47 | 295 | 0.88 | 2.1 | 0.38 | 19.1 | 61.5 | 42.3 | 35.4 | 24.6 | 84.6 | 76.9 | 72.3 | 57.7 |
| 10 | T0658-D1 | 166 | FM | 0 | 70 | 435 | 0.90 | 1.8 | 0.38 | 14.4 | 94.1 | 93.9 | 71.1 | 52.4 | 88.2 | 87.9 | 65.1 | 50.6 |
| 10 | T0658-D2 | 355 | TBM | 12 | 158 | 1140 | 0.95 | 1.5 | 0.66 | 6.7 | 88.9 | 76.1 | 49.4 | 36.6 | 97.2 | 95.8 | 91.0 | 82.8 |
| 10 | T0659-D1 | 74 | TBM | 4 | 35 | 175 | 0.88 | 1.2 | 0.69 | 2.8 | 85.7 | 80.0 | 75.7 | 47.3 | 100.0 | 100.0 | 97.3 | 85.1 |
| 10 | T0661-D1 | 185 | TBM | 100 | 33 | 270 | 0.91 | 1.8 | 0.65 | 4.9 | 63.2 | 51.4 | 32.3 | 26.5 | 100.0 | 97.3 | 85.0 | 62.7 |
| 10 | T0662-D1 | 76 | TBM | 45 | 0 | 105 | 0.90 | 1.1 | 0.31 | 11.9 | 0.0 | 0.0 | 0.0 | 1.3 | 37.5 | 20.0 | 26.3 | 19.7 |
| 10 | T0663-D1 | 86 | TBM | 11 | 35 | 172 | 0.85 | 1.7 | 0.54 | 8.9 | 77.8 | 76.5 | 58.1 | 34.9 | 88.9 | 94.1 | 90.7 | 87.2 |
| 10 | T0663-D2 | 66 | TBM | 11 | 34 | 137 | 0.87 | 1.2 | 0.65 | 3.9 | 85.7 | 84.6 | 66.7 | 39.4 | 85.7 | 84.6 | 93.9 | 92.4 |
| 10 | T0664-D1 | 498 | TBM | 225 | 17 | 1138 | 0.97 | 1.5 | 0.29 | 26.9 | 68.0 | 46.0 | 26.5 | 17.5 | 58.0 | 45.0 | 30.9 | 23.5 |
| 10 | T0666-D1 | 180 | FM | 128 | 0 | 255 | 0.90 | 2.2 | 0.37 | 8.1 | 38.9 | 30.6 | 20.0 | 12.2 | 27.8 | 41.7 | 26.7 | 18.9 |
| 10 | T0667-D1 | 192 | TBM | 67 | 37 | 423 | 0.95 | 1.2 | 0.70 | 4.9 | 84.2 | 81.6 | 72.9 | 54.7 | 89.5 | 84.2 | 84.4 | 71.4 |
| 10 | T0668-D1 | 78 | TBM-HA | 33 | 4 | 84 | 0.79 | 2.4 | 0.28 | 15.2 | 12.5 | 6.7 | 7.9 | 5.5 | 16.7 | 7.7 | 6.1 | 8.8 |
| 10 | T0669-D1 | 97 | TBM | 31 | 26 | 194 | 0.89 | 1.3 | 0.50 | 6.1 | 60.0 | 57.9 | 38.8 | 35.1 | 80.0 | 84.2 | 61.2 | 50.5 |
| 10 | T0671-D1 | 88 | TBM | 0 | 34 | 173 | 0.73 | 2.5 | 0.47 | 8.9 | 100.0 | 83.3 | 68.2 | 48.9 | 100.0 | 94.4 | 86.4 | 72.7 |
| 10 | T0671-D2 | 303 | TBM-HA | 6 | 73 | 945 | 0.93 | 1.9 | 0.26 | 16.0 | 26.7 | 16.4 | 9.2 | 6.9 | 26.7 | 16.4 | 19.7 | 18.8 |
| 10 | T0672-D1 | 312 | TBM | 116 | 52 | 738 | 0.96 | 1.4 | 0.34 | 21.3 | 90.3 | 83.9 | 57.1 | 34.6 | 87.1 | 72.6 | 50.6 | 37.5 |
| 10 | T0673-D1 | 62 | TBM | 0 | 23 | 113 | 0.69 | 2.2 | 0.37 | 10.4 | 50.0 | 50.0 | 41.9 | 27.9 | 100.0 | 100.0 | 90.3 | 70.5 |
| 10 | T0674-D1 | 161 | TBM | 85 | 6 | 300 | 0.93 | 1.4 | 0.55 | 12.2 | 93.8 | 65.6 | 42.0 | 26.1 | 93.8 | 90.6 | 70.4 | 50.9 |
| 10 | T0674-D2 | 134 | TBM | 35 | 32 | 287 | 0.90 | 1.5 | 0.69 | 6.6 | 100.0 | 96.3 | 76.1 | 56.0 | 100.0 | 85.2 | 88.1 | 80.6 |
| 10 | T0676-D1 | 173 | TBM-HA | 50 | 62 | 326 | 0.86 | 2.4 | 0.48 | 9.7 | 94.1 | 77.1 | 54.0 | 32.4 | 100.0 | 100.0 | 90.8 | 75.7 |
| 10 | T0677-D2 | 72 | TBM | 51 | 0 | 53 | 0.83 | 1.6 | 0.60 | 4.6 | 42.9 | 35.7 | 28.6 | 16.2 | 71.4 | 50.0 | 37.1 | 29.4 |
| 10 | T0678-D1 | 154 | TBM-HA | 105 | 0 | 183 | 0.86 | 2.3 | 0.45 | 6.6 | 46.7 | 29.0 | 18.2 | 16.2 | 40.0 | 41.9 | 36.4 | 27.3 |
| 10 | T0679-D1 | 199 | TBM | 85 | 33 | 425 | 0.94 | 1.4 | 0.54 | 9.9 | 90.0 | 82.5 | 61.0 | 47.7 | 75.0 | 70.0 | 49.0 | 38.7 |
| 10 | T0680-D1 | 96 | TBM | 79 | 0 | 108 | 0.85 | 1.9 | 0.68 | 3.5 | 70.0 | 73.7 | 58.3 | 38.5 | 100.0 | 89.5 | 70.8 | 55.2 |
| 10 | T0681-D1 | 197 | TBM | 0 | 101 | 560 | 0.94 | 1.4 | 0.34 | 12.6 | 90.0 | 76.9 | 45.5 | 30.5 | 85.0 | 79.5 | 63.6 | 52.8 |
| 10 | T0682-D1 | 203 | TBM | 171 | 0 | 313 | 0.89 | 2.3 | 0.52 | 6.3 | 70.0 | 51.2 | 37.3 | 26.6 | 70.0 | 53.7 | 42.2 | 32.5 |
| 10 | T0683-D1 | 371 | TBM | 142 | 69 | 938 | 0.97 | 1.3 | 0.33 | 21.7 | 94.6 | 83.8 | 62.2 | 46.0 | 94.6 | 93.2 | 65.8 | 47.4 |
| 10 | T0684-D1 | 73 | TBM | 13 | 24 | 132 | 0.81 | 2.1 | 0.34 | 8.5 | 0.0 | 13.3 | 24.3 | 13.7 | 14.3 | 33.3 | 35.1 | 31.5 |
| 10 | T0684-D2 | 168 | FM | 76 | 31 | 299 | 0.91 | 1.6 | 0.29 | 15.6 | 41.2 | 41.2 | 34.5 | 31.0 | 35.3 | 35.3 | 36.9 | 33.3 |
| 10 | T0685-D1 | 72 | TBM | 54 | 0 | 42 | 0.77 | 2.8 | 0.60 | 6.6 | 85.7 | 57.1 | 27.8 | 19.4 | 85.7 | 78.6 | 44.4 | 38.9 |
| 10 | T0685-D2 | 137 | TBM | 51 | 42 | 320 | 0.93 | 1.3 | 0.42 | 13.7 | 78.6 | 55.6 | 36.2 | 20.4 | 92.9 | 74.1 | 60.9 | 51.1 |
| 10 | T0686-D1 | 185 | TBM | 80 | 37 | 425 | 0.94 | 1.3 | 0.42 | 11.4 | 89.5 | 78.4 | 48.4 | 29.2 | 42.1 | 35.1 | 34.4 | 27.6 |
| 10 | T0687-D1 | 200 | TBM | 75 | 39 | 466 | 0.95 | 1.3 | 0.45 | 13.8 | 90.0 | 82.5 | 64.0 | 43.0 | 65.0 | 57.5 | 42.0 | 35.5 |
| 10 | T0688-D1 | 185 | TBM | 20 | 27 | 470 | 0.91 | 1.6 | 0.30 | 14.8 | 47.4 | 32.4 | 16.1 | 8.7 | 21.1 | 21.6 | 29.0 | 28.1 |
| 10 | T0689-D1 | 211 | TBM | 4 | 129 | 569 | 0.90 | 2.0 | 0.40 | 13.3 | 66.7 | 47.6 | 30.5 | 23.3 | 95.2 | 92.9 | 89.5 | 80.5 |
| 10 | T0690-D1 | 220 | TBM-HA | 0 | 63 | 641 | 0.88 | 2.5 | 0.25 | 15.3 | 27.3 | 15.9 | 6.4 | 5.0 | 72.7 | 68.2 | 40.0 | 29.6 |
| 10 | T0690-D2 | 157 | TBM | 5 | 39 | 423 | 0.89 | 1.8 | 0.23 | 15.4 | 6.3 | 6.5 | 3.8 | 2.6 | 43.8 | 35.5 | 20.3 | 12.7 |
| 10 | T0691-D1 | 106 | TBM | 0 | 72 | 232 | 0.82 | 2.2 | 0.52 | 5.0 | 45.5 | 47.6 | 22.6 | 12.3 | 90.9 | 95.2 | 88.7 | 78.3 |
| 10 | T0692-D1 | 470 | TBM | 181 | 101 | 1147 | 0.94 | 3.1 | 0.64 | 11.7 | 95.7 | 91.5 | 80.9 | 66.4 | 95.7 | 95.7 | 85.1 | 75.7 |
| 10 | T0693-D1 | 100 | FM | 47 | 12 | 101 | 0.44 | 12.0 | 0.39 | 10.5 | 60.0 | 55.0 | 30.0 | 20.0 | 70.0 | 70.0 | 56.0 | 40.0 |
| 10 | T0693-D2 | 219 | TBM | 50 | 62 | 516 | 0.93 | 1.6 | 0.38 | 11.9 | 86.4 | 70.5 | 48.2 | 28.3 | 81.8 | 63.6 | 51.8 | 45.2 |
| 10 | T0694-D1 | 312 | TBM | 103 | 80 | 801 | 0.94 | 1.7 | 0.74 | 5.2 | 100.0 | 93.6 | 87.8 | 72.1 | 100.0 | 98.4 | 93.6 | 84.3 |
| 10 | T0696-D1 | 100 | TBM | 15 | 40 | 209 | 0.72 | 2.9 | 0.26 | 11.9 | 50.0 | 35.0 | 22.0 | 17.0 | 90.0 | 80.0 | 66.0 | 55.0 |
| 10 | T0697-D1 | 458 | TBM | 188 | 56 | 1000 | 0.87 | 5.3 | 0.31 | 18.1 | 87.0 | 70.7 | 49.8 | 33.2 | 87.0 | 77.2 | 52.8 | 39.7 |
| 10 | T0698-D1 | 117 | TBM | 71 | 0 | 158 | 0.93 | 1.2 | 0.60 | 6.1 | 58.3 | 39.1 | 39.0 | 27.4 | 83.3 | 78.3 | 62.7 | 50.4 |
| 10 | T0699-D1 | 225 | TBM | 70 | 54 | 475 | 0.89 | 4.2 | 0.71 | 7.0 | 100.0 | 88.9 | 73.5 | 60.4 | 100.0 | 91.1 | 78.8 | 65.3 |
| 10 | T0701-D1 | 316 | TBM | 134 | 48 | 712 | 0.94 | 2.4 | 0.71 | 8.1 | 100.0 | 93.7 | 89.9 | 71.5 | 100.0 | 96.8 | 91.1 | 76.6 |
| 10 | T0702-D1 | 268 | TBM | 146 | 35 | 454 | 0.76 | 10.6 | 0.58 | 11.9 | 96.3 | 85.2 | 61.2 | 44.4 | 96.3 | 90.7 | 72.4 | 57.5 |
| 10 | T0703-D1 | 272 | TBM | 115 | 52 | 654 | 0.95 | 1.5 | 0.35 | 15.0 | 63.0 | 48.2 | 22.8 | 12.1 | 77.8 | 77.8 | 53.7 | 37.1 |
| 10 | T0704-D1 | 231 | TBM | 78 | 55 | 515 | 0.94 | 1.5 | 0.59 | 8.8 | 100.0 | 95.7 | 84.4 | 63.9 | 100.0 | 95.7 | 89.6 | 78.3 |
| 10 | T0705-D1 | 96 | TBM | 0 | 48 | 229 | 0.85 | 1.8 | 0.45 | 6.3 | 30.0 | 31.6 | 29.2 | 27.1 | 80.0 | 79.0 | 62.5 | 52.1 |
| 10 | T0705-D2 | 344 | TBM-HA | 15 | 150 | 1016 | 0.95 | 1.8 | 0.38 | 16.0 | 23.5 | 20.3 | 14.0 | 13.4 | 91.2 | 88.4 | 82.0 | 71.8 |
| 10 | T0706-D9 | 193 | TBM | 72 | 45 | 500 | 0.95 | 1.3 | 0.42 | 13.1 | 100.0 | 94.9 | 68.0 | 42.0 | 73.7 | 51.3 | 48.5 | 40.4 |
| 10 | T0707-D1 | 321 | TBM | 124 | 42 | 737 | 0.94 | 1.7 | 0.63 | 9.8 | 90.6 | 87.5 | 73.1 | 62.2 | 93.8 | 89.1 | 74.4 | 66.9 |
| 10 | T0708-D1 | 196 | TBM | 56 | 43 | 431 | 0.91 | 1.8 | 0.65 | 5.9 | 90.0 | 89.7 | 87.8 | 75.5 | 90.0 | 89.7 | 87.8 | 77.6 |
| 10 | T0710-D1 | 194 | TBM | 101 | 0 | 375 | 0.90 | 1.8 | 0.31 | 14.5 | 73.7 | 43.6 | 18.6 | 10.3 | 89.5 | 66.7 | 38.1 | 27.3 |
| 10 | T0712-D1 | 186 | TBM | 7 | 91 | 442 | 0.84 | 3.0 | 0.39 | 12.3 | 79.0 | 56.8 | 41.9 | 26.3 | 94.7 | 91.9 | 77.4 | 58.6 |
| 10 | T0713-D1 | 175 | TBM | 0 | 54 | 507 | 0.90 | 1.8 | 0.24 | 14.6 | 5.6 | 5.7 | 2.3 | 1.7 | 55.6 | 42.9 | 27.3 | 24.6 |
| 10 | T0713-D2 | 199 | TBM | 0 | 45 | 585 | 0.87 | 2.3 | 0.26 | 15.1 | 10.0 | 5.0 | 4.0 | 3.0 | 50.0 | 35.0 | 25.0 | 15.6 |
| 10 | T0714-D1 | 88 | TBM | 0 | 37 | 189 | 0.88 | 1.4 | 0.28 | 11.6 | 44.4 | 29.4 | 14.0 | 7.1 | 55.6 | 52.9 | 23.3 | 25.6 |
| 10 | T0715-D1 | 435 | TBM | 177 | 90 | 1164 | 0.95 | 1.7 | 0.52 | 14.2 | 95.5 | 90.8 | 83.9 | 65.1 | 97.7 | 93.1 | 89.5 | 76.6 |
| 10 | T0717-D1 | 135 | TBM | 59 | 28 | 258 | 0.89 | 2.2 | 0.63 | 11.5 | 92.9 | 81.5 | 72.1 | 55.6 | 85.7 | 85.2 | 82.4 | 70.4 |
| 10 | T0717-D2 | 166 | TBM-HA | 25 | 61 | 367 | 0.88 | 1.9 | 0.39 | 9.4 | 17.7 | 15.2 | 10.8 | 12.1 | 88.2 | 93.9 | 88.0 | 67.5 |
| 10 | T0719-D1 | 95 | TBM | 0 | 56 | 228 | 0.87 | 1.5 | 0.22 | 15.0 | 10.0 | 10.5 | 6.3 | 3.2 | 90.0 | 79.0 | 39.6 | 22.1 |
| 10 | T0719-D2 | 61 | TBM | 0 | 35 | 119 | 0.71 | 2.4 | 0.29 | 8.8 | 100.0 | 83.3 | 61.3 | 34.4 | 50.0 | 41.7 | 41.9 | 31.2 |
| 10 | T0719-D3 | 67 | TBM | 0 | 18 | 128 | 0.63 | 4.4 | 0.24 | 9.2 | 42.9 | 30.8 | 29.4 | 19.4 | 71.4 | 69.2 | 52.9 | 35.8 |
| 10 | T0719-D4 | 92 | TBM | 0 | 40 | 218 | 0.88 | 1.4 | 0.29 | 14.5 | 44.4 | 38.9 | 23.9 | 19.6 | 88.9 | 77.8 | 60.9 | 42.4 |
| 10 | T0719-D5 | 115 | TBM | 0 | 45 | 281 | 0.87 | 1.8 | 0.26 | 13.1 | 8.3 | 8.7 | 6.9 | 3.5 | 91.7 | 73.9 | 39.7 | 29.6 |
| 10 | T0719-D6 | 163 | FM | 9 | 46 | 401 | 0.90 | 1.7 | 0.32 | 13.1 | 56.3 | 39.4 | 26.8 | 21.5 | 75.0 | 51.5 | 34.2 | 27.6 |
| 10 | T0720-D1 | 198 | TBM | 77 | 51 | 349 | 0.92 | 1.7 | 0.47 | 10.0 | 90.0 | 72.5 | 48.5 | 36.4 | 95.0 | 87.5 | 70.7 | 51.0 |
| 10 | T0721-D1 | 299 | TBM | 82 | 72 | 717 | 0.94 | 1.8 | 0.32 | 18.7 | 73.3 | 55.0 | 25.3 | 13.4 | 73.3 | 65.0 | 55.3 | 43.1 |
| 10 | T0724-D1 | 119 | TBM | 38 | 40 | 133 | 0.54 | 8.1 | 0.33 | 12.7 | 50.0 | 33.3 | 36.7 | 22.7 | 91.7 | 75.0 | 56.7 | 50.4 |
| 10 | T0724-D2 | 115 | TBM | 0 | 55 | 308 | 0.90 | 1.5 | 0.45 | 9.2 | 83.3 | 78.3 | 62.1 | 48.7 | 83.3 | 82.6 | 74.1 | 60.0 |
| 10 | T0726-D1 | 447 | TBM-HA | 132 | 112 | 1081 | 0.96 | 1.7 | 0.60 | 9.3 | 80.0 | 75.3 | 52.7 | 38.9 | 82.2 | 78.7 | 64.7 | 50.3 |
| 10 | T0726-D2 | 81 | TBM | 10 | 36 | 188 | 0.85 | 1.5 | 0.32 | 14.2 | 0.0 | 0.0 | 0.0 | 0.0 | 62.5 | 68.8 | 63.4 | 49.4 |
| 10 | T0732-D1 | 257 | TBM | 49 | 58 | 671 | 0.94 | 1.6 | 0.57 | 9.0 | 96.2 | 94.1 | 86.1 | 68.1 | 96.2 | 96.1 | 89.2 | 75.9 |
| 10 | T0732-D2 | 91 | TBM-HA | 48 | 0 | 91 | 0.83 | 2.0 | 0.37 | 8.2 | 0.0 | 0.0 | 2.2 | 6.6 | 0.0 | 0.0 | 6.5 | 5.5 |
| 10 | T0733-D1 | 376 | TBM | 124 | 97 | 980 | 0.95 | 1.8 | 0.77 | 5.8 | 97.4 | 94.7 | 86.7 | 72.3 | 97.4 | 98.7 | 92.0 | 81.1 |
| 10 | T0734-D1 | 212 | FM | 101 | 41 | 423 | 0.93 | 1.7 | 0.28 | 15.8 | 14.3 | 14.3 | 11.3 | 10.4 | 71.4 | 50.0 | 28.3 | 22.6 |
| 10 | T0735-D1 | 233 | TBM-HA | 0 | 107 | 652 | 0.93 | 1.7 | 0.34 | 15.7 | 65.2 | 68.1 | 51.3 | 38.6 | 95.7 | 68.1 | 47.9 | 42.9 |
| 10 | T0735-D2 | 88 | FM | 54 | 0 | 94 | 0.87 | 1.4 | 0.50 | 8.4 | 55.6 | 55.6 | 34.1 | 18.2 | 55.6 | 61.1 | 52.3 | 38.6 |
| 10 | T0736-D1 | 166 | TBM | 50 | 66 | 302 | 0.84 | 2.4 | 0.63 | 6.9 | 94.1 | 90.9 | 77.1 | 57.8 | 100.0 | 100.0 | 91.6 | 81.9 |
| 10 | T0737-D1 | 117 | FM | 67 | 0 | 151 | 0.90 | 1.5 | 0.34 | 9.2 | 8.3 | 21.7 | 18.6 | 14.5 | 25.0 | 17.4 | 13.6 | 14.5 |
| 10 | T0738-D1 | 249 | TBM | 112 | 35 | 591 | 0.94 | 1.5 | 0.45 | 11.7 | 84.0 | 70.0 | 52.8 | 40.6 | 48.0 | 52.0 | 41.6 | 34.1 |
| 10 | T0740-D1 | 155 | FM | 100 | 0 | 200 | 0.92 | 1.4 | 0.37 | 13.0 | 18.8 | 19.4 | 16.7 | 12.3 | 31.3 | 29.0 | 18.0 | 16.8 |
| 10 | T0741-D1 | 125 | FM | 0 | 73 | 218 | 0.39 | 13.1 | 0.20 | 15.3 | 0.0 | 0.0 | 7.9 | 5.6 | 23.1 | 20.0 | 22.2 | 20.0 |
| 10 | T0742-D1 | 235 | TBM | 5 | 123 | 567 | 0.89 | 2.3 | 0.29 | 33.7 | 4.4 | 4.3 | 3.5 | 4.7 | 91.3 | 93.6 | 80.3 | 69.2 |
| 10 | T0743-D1 | 114 | TBM | 37 | 43 | 213 | 0.89 | 1.5 | 0.61 | 8.3 | 100.0 | 73.9 | 59.7 | 39.5 | 90.9 | 95.7 | 82.5 | 75.4 |
| 10 | T0744-D1 | 325 | TBM | 110 | 56 | 751 | 0.96 | 1.5 | 0.30 | 19.8 | 66.7 | 50.8 | 31.3 | 19.1 | 78.8 | 61.5 | 40.5 | 29.5 |
| 10 | T0746-D1 | 328 | TBM | 10 | 139 | 941 | 0.93 | 1.9 | 0.30 | 26.2 | 97.0 | 90.9 | 77.4 | 54.6 | 81.8 | 89.4 | 78.7 | 65.9 |
| 10 | T0747-D9 | 90 | TBM | 0 | 52 | 218 | 0.85 | 1.8 | 0.34 | 9.9 | 11.1 | 5.6 | 17.8 | 17.8 | 55.6 | 55.6 | 44.4 | 37.8 |
| 10 | T0749-D1 | 412 | TBM | 166 | 35 | 1040 | 0.96 | 1.6 | 0.72 | 5.9 | 87.8 | 84.2 | 66.0 | 46.6 | 85.4 | 79.3 | 72.3 | 60.4 |
| 10 | T0750-D1 | 182 | TBM | 96 | 26 | 412 | 0.95 | 1.3 | 0.54 | 8.0 | 88.9 | 77.8 | 69.2 | 49.5 | 44.4 | 50.0 | 40.7 | 39.0 |
| 10 | T0752-D1 | 148 | TBM | 49 | 68 | 309 | 0.89 | 1.8 | 0.67 | 5.7 | 86.7 | 90.0 | 85.1 | 64.9 | 93.3 | 90.0 | 91.9 | 87.2 |
| 10 | T0753-D1 | 108 | TBM | 27 | 33 | 220 | 0.89 | 1.7 | 0.62 | 4.6 | 100.0 | 81.8 | 68.5 | 53.7 | 90.9 | 86.4 | 75.9 | 63.0 |
| 10 | T0755-D1 | 258 | TBM | 83 | 68 | 596 | 0.93 | 1.8 | 0.21 | 31.7 | 61.5 | 44.2 | 38.0 | 28.7 | 65.4 | 57.7 | 46.5 | 38.0 |
| 10 | T0756-D1 | 91 | TBM | 36 | 19 | 141 | 0.82 | 2.1 | 0.40 | 6.4 | 44.4 | 22.2 | 8.7 | 7.7 | 77.8 | 77.8 | 65.2 | 40.7 |
| 10 | T0756-D2 | 86 | FM | 45 | 0 | 15 | 0.38 | 15.4 | 0.37 | 12.9 | 0.0 | 0.0 | 0.0 | 2.3 | 11.1 | 5.9 | 4.7 | 3.5 |
| 10 | T0757-D1 | 247 | TBM | 43 | 94 | 558 | 0.91 | 1.9 | 0.65 | 7.6 | 100.0 | 93.9 | 86.3 | 68.8 | 100.0 | 98.0 | 92.7 | 82.2 |
| 10 | T0758-D1 | 366 | TBM | 200 | 50 | 717 | 0.95 | 1.7 | 0.77 | 4.8 | 100.0 | 97.3 | 83.6 | 66.1 | 100.0 | 98.6 | 91.8 | 78.7 |
| 11 | T0759-D2 | 62 | TBM | 22 | 18 | 78 | 0.76 | 2.1 | 0.42 | 4.3 | 0.0 | 0.0 | 9.7 | 6.5 | 100.0 | 91.7 | 71.0 | 61.3 |
| 11 | T0760-D1 | 201 | TBM | 8 | 95 | 502 | 0.88 | 2.2 | 0.35 | 13.0 | 90.0 | 70.0 | 55.5 | 40.8 | 95.0 | 85.0 | 78.2 | 61.7 |
| 11 | T0761-D1 | 88 | FM | 18 | 33 | 156 | 0.87 | 1.4 | 0.25 | 12.3 | 0.0 | 0.0 | 4.6 | 2.3 | 55.6 | 61.1 | 38.6 | 26.1 |
| 11 | T0761-D2 | 113 | FM | 51 | 27 | 171 | 0.88 | 1.7 | 0.39 | 10.5 | 9.1 | 8.7 | 7.0 | 4.4 | 81.8 | 82.6 | 64.9 | 53.1 |
| 11 | T0762-D1 | 257 | TBM | 75 | 67 | 591 | 0.95 | 1.5 | 0.30 | 14.8 | 84.6 | 70.6 | 54.3 | 42.8 | 76.9 | 60.8 | 48.8 | 38.5 |
| 11 | T0763-D1 | 130 | FM | 22 | 54 | 280 | 0.89 | 1.8 | 0.28 | 16.0 | 0.0 | 7.7 | 9.2 | 7.7 | 76.9 | 84.6 | 49.2 | 36.2 |
| 11 | T0764-D1 | 321 | TBM | 85 | 69 | 766 | 0.94 | 1.8 | 0.39 | 19.6 | 71.9 | 67.2 | 50.3 | 39.9 | 78.1 | 62.5 | 52.2 | 38.3 |
| 11 | T0765-D1 | 76 | TBM | 24 | 24 | 151 | 0.90 | 1.2 | 0.48 | 5.0 | 87.5 | 80.0 | 68.4 | 54.0 | 87.5 | 80.0 | 71.1 | 56.6 |
| 11 | T0766-D1 | 108 | TBM | 27 | 59 | 244 | 0.92 | 1.3 | 0.38 | 8.3 | 9.1 | 9.1 | 9.3 | 13.9 | 100.0 | 90.9 | 88.9 | 67.6 |
| 11 | T0767-D1 | 76 | TBM | 25 | 28 | 152 | 0.89 | 1.2 | 0.42 | 8.3 | 50.0 | 40.0 | 23.7 | 17.1 | 75.0 | 66.7 | 52.6 | 50.0 |
| 11 | T0767-D2 | 180 | FM | 76 | 26 | 302 | 0.89 | 2.0 | 0.33 | 11.8 | 66.7 | 61.1 | 45.6 | 33.3 | 61.1 | 55.6 | 38.9 | 31.1 |
| 11 | T0768-D1 | 143 | TBM | 0 | 52 | 391 | 0.86 | 2.0 | 0.27 | 13.2 | 42.9 | 24.1 | 9.7 | 8.4 | 71.4 | 65.5 | 55.6 | 42.7 |
| 11 | T0769-D1 | 97 | TBM | 41 | 35 | 200 | 0.91 | 1.3 | 0.44 | 6.5 | 40.0 | 52.6 | 61.2 | 46.4 | 80.0 | 84.2 | 61.2 | 61.9 |
| 11 | T0770-D1 | 456 | TBM | 211 | 11 | 973 | 0.96 | 1.6 | 0.44 | 13.6 | 87.0 | 65.9 | 44.7 | 25.0 | 69.6 | 56.0 | 42.1 | 32.0 |
| 11 | T0771-D1 | 151 | FM | 34 | 42 | 322 | 0.90 | 1.7 | 0.30 | 14.0 | 26.7 | 30.0 | 26.3 | 17.2 | 73.3 | 66.7 | 51.3 | 40.4 |
| 11 | T0772-D1 | 214 | TBM | 0 | 73 | 595 | 0.92 | 1.7 | 0.63 | 7.3 | 95.2 | 95.4 | 86.0 | 71.0 | 100.0 | 95.4 | 88.8 | 79.4 |
| 11 | T0773-D1 | 67 | TBM | 27 | 21 | 127 | 0.92 | 0.9 | 0.59 | 3.6 | 28.6 | 23.1 | 35.3 | 38.8 | 85.7 | 76.9 | 52.9 | 43.3 |
| 11 | T0774-D1 | 333 | TBM-HA | 11 | 136 | 873 | 0.91 | 2.3 | 0.37 | 16.2 | 69.7 | 74.6 | 65.9 | 48.7 | 78.8 | 76.1 | 72.5 | 55.6 |
| 11 | T0776-D1 | 219 | TBM | 95 | 23 | 472 | 0.95 | 1.3 | 0.69 | 6.5 | 100.0 | 100.0 | 85.5 | 67.1 | 100.0 | 100.0 | 88.2 | 69.4 |
| 11 | T0777-D1 | 345 | FM | 179 | 8 | 613 | 0.95 | 1.7 | 0.35 | 17.3 | 25.7 | 24.6 | 13.3 | 11.0 | 57.1 | 44.9 | 31.8 | 24.1 |
| 11 | T0780-D1 | 95 | TBM | 8 | 52 | 217 | 0.84 | 1.8 | 0.60 | 4.3 | 100.0 | 100.0 | 95.8 | 82.1 | 100.0 | 100.0 | 93.8 | 91.6 |
| 11 | T0780-D2 | 96 | TBM | 5 | 46 | 224 | 0.82 | 2.1 | 0.68 | 3.3 | 90.0 | 84.2 | 85.4 | 76.0 | 90.0 | 84.2 | 89.6 | 86.5 |
| 11 | T0781-D1 | 200 | FM | 65 | 39 | 422 | 0.93 | 1.7 | 0.23 | 21.2 | 5.0 | 2.5 | 8.0 | 8.0 | 90.0 | 77.5 | 50.0 | 32.0 |
| 11 | T0781-D2 | 175 | TBM-HA | 60 | 41 | 348 | 0.90 | 1.8 | 0.27 | 14.7 | 27.8 | 25.7 | 15.9 | 9.1 | 83.3 | 77.1 | 60.2 | 48.0 |
| 11 | T0782-D1 | 110 | TBM | 8 | 67 | 228 | 0.83 | 2.0 | 0.40 | 7.8 | 27.3 | 36.4 | 27.3 | 22.7 | 100.0 | 100.0 | 80.0 | 71.8 |
| 11 | T0783-D1 | 243 | TBM | 85 | 53 | 536 | 0.88 | 3.6 | 0.67 | 7.9 | 79.2 | 77.6 | 65.6 | 51.0 | 83.3 | 81.6 | 76.2 | 61.7 |
| 11 | T0783-D2 | 156 | TBM | 70 | 37 | 328 | 0.93 | 1.6 | 0.39 | 11.3 | 62.5 | 54.8 | 53.9 | 42.3 | 62.5 | 54.8 | 55.1 | 42.3 |
| 11 | T0784-D1 | 125 | TBM | 0 | 76 | 348 | 0.90 | 1.7 | 0.30 | 12.4 | 7.7 | 4.0 | 12.7 | 14.4 | 23.1 | 28.0 | 28.6 | 22.4 |
| 11 | T0785-D1 | 112 | FM | 8 | 65 | 275 | 0.87 | 1.7 | 0.25 | 14.6 | 9.1 | 4.6 | 8.9 | 8.0 | 72.7 | 54.6 | 37.5 | 26.8 |
| 11 | T0786-D1 | 217 | TBM | 50 | 67 | 446 | 0.91 | 2.0 | 0.56 | 11.3 | 54.6 | 48.8 | 44.0 | 33.2 | 81.8 | 83.7 | 71.6 | 60.8 |
| 11 | T0789-D1 | 143 | FM | 45 | 35 | 258 | 0.86 | 2.3 | 0.43 | 10.0 | 50.0 | 51.7 | 40.3 | 30.8 | 100.0 | 89.7 | 76.4 | 62.9 |
| 11 | T0789-D2 | 126 | FM | 42 | 37 | 232 | 0.90 | 1.6 | 0.40 | 15.1 | 61.5 | 52.0 | 30.2 | 23.0 | 84.6 | 84.0 | 74.6 | 64.3 |
| 11 | T0790-D1 | 135 | FM | 44 | 35 | 246 | 0.90 | 1.8 | 0.61 | 6.8 | 64.3 | 70.4 | 50.0 | 37.0 | 100.0 | 96.3 | 86.8 | 76.3 |
| 11 | T0790-D2 | 130 | FM | 40 | 39 | 226 | 0.86 | 1.9 | 0.39 | 10.7 | 61.5 | 42.3 | 33.9 | 23.9 | 100.0 | 92.3 | 84.6 | 62.3 |
| 11 | T0791-D1 | 149 | FM | 43 | 39 | 259 | 0.86 | 2.3 | 0.39 | 10.9 | 86.7 | 70.0 | 48.0 | 36.9 | 93.3 | 93.3 | 77.3 | 55.7 |
| 11 | T0791-D2 | 138 | FM | 40 | 36 | 291 | 0.85 | 2.3 | 0.37 | 19.1 | 57.1 | 50.0 | 36.2 | 27.5 | 100.0 | 92.9 | 76.8 | 61.6 |
| 11 | T0792-D1 | 78 | TBM | 34 | 10 | 104 | 0.86 | 1.4 | 0.42 | 8.3 | 12.5 | 6.3 | 5.1 | 5.1 | 87.5 | 75.0 | 66.7 | 41.0 |
| 11 | T0794-D1 | 288 | TBM | 57 | 76 | 766 | 0.94 | 1.7 | 0.69 | 8.6 | 96.6 | 91.4 | 81.3 | 64.6 | 96.6 | 94.8 | 89.6 | 76.4 |
| 11 | T0794-D2 | 172 | FM | 4 | 80 | 495 | 0.93 | 1.4 | 0.35 | 11.8 | 52.9 | 44.1 | 34.9 | 29.1 | 58.8 | 67.7 | 57.0 | 41.9 |
| 11 | T0796-D1 | 296 | TBM | 21 | 177 | 807 | 0.76 | 4.7 | 0.24 | 22.8 | 50.0 | 47.5 | 46.6 | 35.8 | 50.0 | 45.8 | 46.0 | 38.2 |
| 11 | T0800-D1 | 212 | TBM-HA | 0 | 135 | 631 | 0.90 | 2.0 | 0.41 | 12.2 | 95.2 | 92.9 | 84.9 | 72.2 | 81.0 | 83.3 | 81.1 | 70.8 |
| 11 | T0801-D1 | 360 | TBM | 135 | 49 | 896 | 0.96 | 1.5 | 0.59 | 11.3 | 100.0 | 91.7 | 75.0 | 54.7 | 100.0 | 94.4 | 85.0 | 70.3 |
| 11 | T0803-D1 | 134 | TBM | 38 | 27 | 289 | 0.93 | 1.3 | 0.31 | 12.7 | 30.8 | 25.9 | 31.3 | 24.6 | 76.9 | 77.8 | 46.3 | 35.8 |
| 11 | T0805-D1 | 197 | TBM | 69 | 25 | 341 | 0.82 | 6.3 | 0.65 | 12.6 | 100.0 | 89.7 | 86.9 | 70.6 | 100.0 | 89.7 | 88.9 | 76.1 |
| 11 | T0806-D1 | 256 | FM | 86 | 47 | 548 | 0.94 | 1.5 | 0.71 | 4.5 | 76.9 | 76.5 | 60.9 | 53.1 | 96.2 | 80.4 | 73.4 | 62.1 |
| 11 | T0807-D1 | 283 | TBM | 99 | 44 | 660 | 0.96 | 1.4 | 0.82 | 5.6 | 100.0 | 94.7 | 87.3 | 74.2 | 100.0 | 98.3 | 89.4 | 80.9 |
| 11 | T0808-D1 | 131 | TBM | 4 | 90 | 373 | 0.92 | 1.4 | 0.45 | 8.7 | 92.3 | 96.2 | 81.8 | 68.7 | 92.3 | 92.3 | 78.8 | 60.3 |
| 11 | T0808-D2 | 269 | FM | 15 | 125 | 799 | 0.91 | 2.2 | 0.37 | 15.4 | 48.2 | 50.0 | 34.1 | 25.7 | 85.2 | 87.0 | 69.6 | 55.8 |
| 11 | T0810-D1 | 113 | FM | 77 | 0 | 122 | 0.87 | 1.8 | 0.37 | 12.4 | 27.3 | 21.7 | 17.5 | 12.4 | 36.4 | 26.1 | 22.8 | 15.0 |
| 11 | T0810-D2 | 225 | TBM | 85 | 42 | 542 | 0.94 | 1.6 | 0.61 | 14.5 | 95.7 | 97.8 | 85.8 | 72.0 | 91.3 | 95.6 | 86.7 | 77.3 |
| 11 | T0811-D1 | 251 | TBM | 104 | 38 | 599 | 0.96 | 1.2 | 0.82 | 3.8 | 76.0 | 84.0 | 76.2 | 68.5 | 80.0 | 86.0 | 78.6 | 74.5 |
| 11 | T0812-D1 | 182 | TBM-HA | 9 | 73 | 441 | 0.88 | 2.3 | 0.28 | 15.7 | 66.7 | 58.3 | 52.8 | 40.1 | 50.0 | 61.1 | 46.2 | 40.1 |
| 11 | T0813-D1 | 302 | TBM | 142 | 40 | 580 | 0.89 | 3.3 | 0.53 | 16.3 | 83.3 | 81.7 | 70.9 | 53.3 | 90.0 | 83.3 | 75.5 | 61.6 |
| 11 | T0814-D1 | 137 | FM | 4 | 63 | 323 | 0.90 | 1.6 | 0.35 | 17.7 | 85.7 | 77.8 | 50.7 | 40.2 | 85.7 | 85.2 | 62.3 | 48.2 |
| 11 | T0814-D2 | 116 | FM | 4 | 56 | 274 | 0.89 | 1.6 | 0.40 | 9.2 | 41.7 | 30.4 | 20.7 | 17.2 | 91.7 | 69.6 | 55.2 | 42.2 |
| 11 | T0814-D3 | 144 | TBM-HA | 4 | 58 | 335 | 0.89 | 1.8 | 0.35 | 17.0 | 85.7 | 82.8 | 66.7 | 50.7 | 100.0 | 89.7 | 70.8 | 56.3 |
| 11 | T0815-D1 | 106 | TBM | 31 | 49 | 212 | 0.90 | 1.4 | 0.35 | 16.0 | 63.6 | 42.9 | 28.3 | 19.8 | 90.9 | 85.7 | 92.5 | 71.7 |
| 11 | T0816-D1 | 68 | TBM | 47 | 0 | 74 | 0.92 | 0.9 | 0.51 | 8.7 | 42.9 | 21.4 | 17.7 | 14.7 | 71.4 | 57.1 | 32.4 | 26.5 |
| 11 | T0817-D1 | 265 | TBM | 43 | 74 | 662 | 0.94 | 1.7 | 0.60 | 11.0 | 100.0 | 98.1 | 82.0 | 62.3 | 100.0 | 98.1 | 95.5 | 84.9 |
| 11 | T0817-D2 | 210 | TBM | 76 | 35 | 460 | 0.93 | 1.7 | 0.73 | 4.3 | 100.0 | 97.6 | 89.5 | 69.5 | 100.0 | 97.6 | 90.5 | 77.1 |
| 11 | T0818-D1 | 134 | TBM | 38 | 50 | 271 | 0.90 | 1.6 | 0.31 | 13.4 | 38.5 | 37.0 | 32.8 | 26.9 | 76.9 | 55.6 | 59.7 | 48.5 |
| 11 | T0819-D1 | 367 | TBM | 156 | 50 | 826 | 0.93 | 2.7 | 0.43 | 15.8 | 91.9 | 78.1 | 58.7 | 40.1 | 89.2 | 76.7 | 56.0 | 42.8 |
| 11 | T0820-D1 | 90 | FM | 65 | 0 | 72 | 0.81 | 3.0 | 0.35 | 13.2 | 11.1 | 5.6 | 2.2 | 2.2 | 0.0 | 11.1 | 4.4 | 5.6 |
| 11 | T0821-D1 | 255 | TBM | 195 | 0 | 378 | 0.75 | 4.3 | 0.39 | 17.4 | 69.2 | 41.2 | 16.4 | 9.8 | 100.0 | 86.3 | 55.5 | 43.1 |
| 11 | T0822-D1 | 114 | TBM | 4 | 60 | 300 | 0.91 | 1.3 | 0.33 | 10.0 | 54.6 | 56.5 | 54.4 | 47.4 | 81.8 | 73.9 | 52.6 | 46.5 |
| 11 | T0823-D1 | 288 | TBM | 103 | 49 | 564 | 0.91 | 2.2 | 0.75 | 4.8 | 86.2 | 86.2 | 70.8 | 60.1 | 89.7 | 86.2 | 72.9 | 65.3 |
| 11 | T0824-D1 | 108 | FM | 35 | 24 | 218 | 0.89 | 1.5 | 0.56 | 5.0 | 90.9 | 72.7 | 63.0 | 51.9 | 90.9 | 77.3 | 64.8 | 55.6 |
| 11 | T0827-D1 | 193 | TBM | 128 | 0 | 293 | 0.90 | 2.0 | 0.51 | 7.2 | 73.7 | 69.2 | 39.2 | 32.1 | 79.0 | 56.4 | 56.7 | 46.1 |
| 11 | T0827-D2 | 150 | FM | 102 | 0 | 208 | 0.93 | 1.4 | 0.51 | 7.0 | 53.3 | 46.7 | 29.3 | 19.3 | 86.7 | 70.0 | 54.7 | 37.3 |
| 11 | T0829-D1 | 67 | TBM | 17 | 22 | 122 | 0.82 | 1.6 | 0.65 | 3.0 | 71.4 | 69.2 | 47.1 | 28.4 | 100.0 | 100.0 | 79.4 | 76.1 |
| 11 | T0830-D1 | 417 | TBM-HA | 294 | 4 | 743 | 0.95 | 1.9 | 0.54 | 18.9 | 92.9 | 86.8 | 67.9 | 45.6 | 90.5 | 88.0 | 77.0 | 52.8 |
| 11 | T0830-D2 | 111 | TBM | 32 | 24 | 208 | 0.87 | 1.7 | 0.36 | 7.7 | 54.6 | 40.9 | 37.5 | 27.0 | 72.7 | 72.7 | 48.2 | 46.0 |
| 11 | T0831-D1 | 155 | TBM-HA | 114 | 0 | 141 | 0.67 | 14.1 | 0.38 | 22.2 | 50.0 | 32.3 | 16.7 | 9.7 | 93.8 | 61.3 | 34.6 | 21.3 |
| 11 | T0831-D2 | 197 | FM | 151 | 0 | 241 | 0.91 | 1.9 | 0.32 | 17.4 | 20.0 | 12.8 | 7.1 | 6.1 | 50.0 | 41.0 | 24.2 | 15.2 |
| 11 | T0832-D1 | 209 | FM | 108 | 0 | 383 | 0.95 | 1.4 | 0.29 | 13.6 | 4.8 | 4.8 | 9.5 | 7.2 | 28.6 | 33.3 | 24.8 | 16.8 |
| 11 | T0833-D1 | 108 | TBM | 0 | 76 | 212 | 0.77 | 2.5 | 0.41 | 13.9 | 9.1 | 13.6 | 7.4 | 6.5 | 81.8 | 72.7 | 77.8 | 73.2 |
| 11 | T0834-D1 | 99 | FM | 42 | 20 | 121 | 0.86 | 1.7 | 0.36 | 20.3 | 0.0 | 10.0 | 4.0 | 2.0 | 100.0 | 80.0 | 54.0 | 36.4 |
| 11 | T0834-D2 | 86 | FM | 43 | 0 | 110 | 0.84 | 2.0 | 0.29 | 14.6 | 11.1 | 5.9 | 4.7 | 2.3 | 11.1 | 11.8 | 20.9 | 16.3 |
| 11 | T0835-D1 | 404 | TBM | 203 | 53 | 939 | 0.96 | 1.4 | 0.63 | 10.6 | 75.0 | 65.4 | 44.1 | 32.9 | 95.0 | 85.2 | 63.4 | 47.5 |
| 11 | T0836-D1 | 204 | FM | 157 | 0 | 198 | 0.80 | 4.0 | 0.42 | 13.1 | 35.0 | 26.8 | 19.6 | 13.2 | 35.0 | 26.8 | 20.6 | 15.2 |
| 11 | T0837-D1 | 121 | FM | 86 | 0 | 157 | 0.94 | 1.1 | 0.43 | 6.5 | 50.0 | 33.3 | 16.4 | 15.7 | 33.3 | 37.5 | 29.5 | 21.5 |
| 11 | T0838-D1 | 126 | TBM | 23 | 50 | 257 | 0.84 | 2.1 | 0.29 | 14.9 | 61.5 | 48.0 | 38.1 | 23.8 | 84.6 | 84.0 | 69.8 | 54.8 |
| 11 | T0840-D1 | 519 | other | 34 | 186 | 1476 | 0.95 | 2.1 | 0.26 | 34.4 | 48.1 | 29.8 | 17.7 | 12.1 | 94.2 | 89.4 | 81.5 | 60.5 |
| 11 | T0840-D2 | 92 | other | 0 | 42 | 222 | 0.76 | 2.5 | 0.39 | 8.6 | 100.0 | 77.8 | 60.9 | 41.3 | 100.0 | 94.4 | 56.5 | 55.4 |
| 11 | T0841-D1 | 231 | other | 16 | 51 | 621 | 0.93 | 1.6 | 0.33 | 14.6 | 39.1 | 47.8 | 41.4 | 29.0 | 82.6 | 60.9 | 51.7 | 45.0 |
| 11 | T0843-D1 | 369 | TBM | 136 | 51 | 812 | 0.95 | 1.9 | 0.30 | 26.5 | 91.9 | 64.9 | 40.0 | 22.5 | 83.8 | 70.3 | 50.3 | 39.0 |
| 11 | T0845-D1 | 97 | TBM | 0 | 40 | 206 | 0.74 | 4.7 | 0.23 | 16.2 | 20.0 | 10.5 | 6.1 | 4.1 | 70.0 | 42.1 | 26.5 | 18.6 |
| 11 | T0845-D2 | 329 | TBM | 10 | 148 | 928 | 0.95 | 1.7 | 0.63 | 7.3 | 72.7 | 68.2 | 49.1 | 38.3 | 97.0 | 92.4 | 89.1 | 79.9 |
| 11 | T0847-D1 | 169 | TBM | 48 | 50 | 357 | 0.90 | 2.0 | 0.69 | 4.9 | 100.0 | 97.1 | 83.5 | 70.4 | 100.0 | 100.0 | 90.6 | 77.5 |
| 11 | T0848-D1 | 138 | TBM | 12 | 59 | 344 | 0.91 | 1.5 | 0.47 | 9.3 | 100.0 | 100.0 | 94.2 | 73.2 | 100.0 | 100.0 | 97.1 | 87.7 |
| 11 | T0848-D2 | 183 | TBM-HA | 92 | 35 | 310 | 0.86 | 2.6 | 0.38 | 14.4 | 100.0 | 83.8 | 53.3 | 34.4 | 100.0 | 94.6 | 78.3 | 56.8 |
| 11 | T0849-D1 | 236 | TBM | 130 | 17 | 394 | 0.94 | 1.6 | 0.66 | 11.3 | 100.0 | 93.6 | 73.7 | 50.4 | 95.8 | 93.6 | 73.7 | 58.5 |
| 11 | T0851-D1 | 453 | TBM | 195 | 63 | 1079 | 0.96 | 1.8 | 0.72 | 7.3 | 97.8 | 90.1 | 79.7 | 62.7 | 97.8 | 93.4 | 84.1 | 71.5 |
| 11 | T0852-D1 | 234 | TBM | 86 | 66 | 535 | 0.95 | 1.4 | 0.74 | 3.9 | 100.0 | 91.5 | 85.5 | 72.2 | 100.0 | 93.6 | 87.2 | 76.9 |
| 11 | T0852-D2 | 126 | TBM | 86 | 0 | 184 | 0.92 | 1.4 | 0.60 | 5.5 | 53.9 | 40.0 | 33.3 | 26.2 | 76.9 | 76.0 | 60.3 | 46.0 |
| 11 | T0853-D1 | 76 | TBM | 12 | 24 | 143 | 0.83 | 1.7 | 0.32 | 12.3 | 37.5 | 40.0 | 34.2 | 27.6 | 87.5 | 80.0 | 68.4 | 43.4 |
| 11 | T0853-D2 | 72 | TBM | 13 | 26 | 137 | 0.81 | 2.1 | 0.31 | 10.7 | 0.0 | 14.3 | 16.7 | 16.7 | 57.1 | 50.0 | 33.3 | 22.2 |
| 11 | T0854-D1 | 132 | TBM | 36 | 24 | 295 | 0.92 | 1.4 | 0.64 | 5.7 | 76.9 | 76.9 | 56.1 | 44.7 | 100.0 | 92.3 | 86.4 | 74.2 |
| 11 | T0854-D2 | 70 | TBM | 42 | 0 | 66 | 0.86 | 1.4 | 0.57 | 3.8 | 42.9 | 35.7 | 28.6 | 18.6 | 85.7 | 78.6 | 45.7 | 35.7 |
| 11 | T0855-D1 | 115 | FM | 35 | 27 | 207 | 0.87 | 1.8 | 0.37 | 9.8 | 0.0 | 13.0 | 15.5 | 13.0 | 66.7 | 69.6 | 53.5 | 47.0 |
| 11 | T0856-D1 | 159 | TBM | 0 | 77 | 460 | 0.92 | 1.5 | 0.54 | 7.7 | 87.5 | 81.3 | 76.3 | 55.4 | 100.0 | 93.8 | 76.3 | 68.6 |
| 11 | T0857-D1 | 96 | TBM | 0 | 50 | 198 | 0.83 | 1.9 | 0.36 | 13.3 | 10.0 | 26.3 | 16.7 | 15.6 | 100.0 | 73.7 | 54.2 | 39.6 |
| 11 | T0858-D1 | 450 | TBM | 115 | 127 | 1254 | 0.96 | 1.6 | 0.71 | 7.0 | 95.6 | 85.6 | 78.2 | 64.9 | 95.6 | 94.4 | 81.8 | 71.3 |
| **Average** | | | | | | | **0.88** | **2.0** | **0.48** | **10.5** | **65.2** | **58.70** | **47.1** | **35.8** | **79.4** | **74.32** | **63.5** | **52.5** |
